# What Happens to Population Health When the Doctors Leave? Evidence from the Exit of Cuban Doctors in Brazil*

**DOI:** 10.1016/j.jhealeco.2025.103099

**Published:** 2026-01-05

**Authors:** Stefan Sliwa Ruiz, Malte Becker, Thomas Hone, Rudi Rocha

**Affiliations:** † Faculty of Economics & Business, University of Groningen, the Netherlands; ‡ International Development Group, IfW Kiel, Germany; § Public Health Policy Evaluation Unit, School of Public Health, Imperial College London, United Kingdom

**Keywords:** doctor supply, health outcomes, healthcare utilization, I10, I15, I18

## Abstract

This paper studies the effects of a large-scale exit of doctors on population health outcomes, health production inputs, outputs, and health system adaptation in Brazil. Identification exploits the exogenous timing of the Cuban exit from municipalities that relied more *versus* less on Cuban doctors within the More Doctors Program. We find persistent reductions in the care of chronic diseases, while service utilization for conditions requiring immediate care, such as maternal-related services and infections, quickly recovered. Reductions in utilization did not translate into changes in health outcomes. Supply-side response and demand diversion helped mitigate major adverse repercussions for population health at the market level.

## 1 Introduction

Developed and developing countries alike have large regional disparities in the provision of medical doctors, with remote and under-served areas often facing a more limited supply of personnel. Retaining doctors in these areas remains a major policy challenge (WHO, 2010). Low retention rates can negatively affect healthcare quality and health outcomes due to understaffed services and discontinuity of care (WHO, 2018). Moreover, as the global demand for healthcare professionals continues to grow, difficulties in doctor attraction and retention are projected to increase, especially in remote areas (Esu et al., 2021; Liu et al., 2017). Yet, causal evidence regarding the extent to which and how doctor supply affects population health remains limited. Most existing studies focus on the population health impacts of increases in the supply of personnel. Very little is known about how doctor exit affects population health outcomes, in particular in resource-scarce contexts, how health systems adapt, and how health production inputs and outputs respond to doctor exit at the market level, in general.

This paper studies the effects of a unique large-scale doctor withdrawal event on population health outcomes, and on health production inputs and outputs by exploiting an unexpected shock to local health systems in Brazil. To overcome a chronic lack of doctors in under-served areas, in 2013 Brazil introduced the More Doctors Program (*Programa Mais Médicos* – PMM). Over 18,000 doctors served in primary care services in eligible municipalities, roughly half of them being from Cuba. Five years after the introduction of PMM, following the election of Brazil’s former right-wing president Jair Bolsonaro in October 2018, the Cuban government unilaterally withdrew all of the Cuban doctors from the program. The sudden exit led to a major shock to the supply of healthcare where Cuban doctors had been working. Some municipalities were suddenly left without PMM doctors. Other municipalities, where no Cuban doctor had been working, remained unaffected.

We exploit the sharp and unexpected exogenous timing of the Cuban exit from PMM in municipalities that relied more *versus* less on Cuban PMM doctors at the time of the withdrawal to estimate effects of doctor exit on population health outcomes, health production inputs (such as availability of personnel and health facilities) and outputs (such as number of consultations and medical procedures by complexity level). More specifically, we use an event-study approach to compare outcome changes in affected and unaffected PMM municipalities before and after the Cuban exit, conditional upon time and municipality fixed effects, therefore controlling for baseline heterogeneity in levels and common time trends. We combine different fine-grained administrative microdata sets on healthcare utilization, hospitalization and mortality, as well as on the supply of healthcare facilities, personnel, prescription drug sales and private health insurance into a municipality-by-month panel of data between October 2017 and December 2019. The overall evidence indicates that pre-trends in outcome variables remain irrelevant, while alternative specifications, alternative treatment definitions and robustness checks lend support to our identification strategy.

We find an immediate drop in the number of primary care doctors in affected municipalities from November 2018 on. That reduction is particularly pronounced for municipalities where all PMM doctors were Cuban. From January 2019 on, affected municipalities started to fill PMM vacancies, but only by mid-2019 doctor supply stabilized, almost reaching pre-treatment levels. Next, we document meaningful reductions in primary care utilization. Effects are concentrated in physician duties, and most persist even after the arrival of new doctors. Persistent reduction in service utilization is primarily driven by conditions requiring prevention and ongoing care, such as mental health and chronic diseases, and related risk factors. For example, hypertension consultations decline on average by 20% in the most affected municipalities immediately after the Cuban exit, and remain 14% lower than base levels. Importantly, while there was a strong and persistent decrease in the care of mental health and chronic diseases, service utilization for conditions requiring immediate care, such as infections and maternal-related services, remained relatively stable. Finally, the reduction in healthcare utilization did not translate into any systematic changes in health outcomes even throughout the end of the following year after the Cuban exit. We do not observe any effects on hospital admissions or mortality, neither on aggregate levels nor from specific causes.

Adaptation of local health systems and demand diversion seem to have helped mitigate the negative shock to doctor supply without major adverse repercussions for population health outcomes. We explore different intermediate outcomes that could explain the overall findings. We first focus on adaptation within primary care services. We do not find any changes in the supply of other health professionals nor in task substitution across different professionals. Moreover, we do not observe an improvement in the experience profile of the new doctors who replaced the Cuban professionals. Yet, patients in need of prescription medication for chronic diseases were able to secure their access despite the reduction in access to other related healthcare services, such as consultations. Together with the rapid recovery of consultations for conditions requiring immediate care, the overall evidence indicates a prioritization of primary care services towards urgent and curative needs as well as other protective services.

Second, we investigate system adaptation and demand diversion beyond primary care services. While there were no significant changes in the use of hospitals and specialists, we observe a suggestive increase in ER utilization for basic ambulatory care as well as in the number of ERs offering such services, particularly in municipalities where all PMM doctors were Cuban. Patients therefore seem to partly substitute treatment at primary care centers with treatment at ER facilities, while municipalities start providing more basic services in these facilities. Importantly, this diversion persists over time. Finally, we do not observe any changes in private insurance coverage in affected municipalities nor detect any spillover effects across municipalities. Adaption therefore comes at the cost of a reduction in preventive care in primary care services and an increase in utilization of ER facilities. We conjecture that this could entail fragmentation and decreased efficiency in healthcare provision, with potential negative long-term consequences for tackling the increasing burden of chronic diseases.

This paper contributes novel evidence to the emergent literature on the impacts of doctor supply, more generally, and of doctor exit, more specifically, on population health. Existing studies document positive associations between the supply of health professionals and health outcomes, but often struggle to establish causality (Anand & Bärnighausen, 2004; Basu et al., 2019). Causally identified studies are scant and document a nuanced picture, especially in resource-scarce contexts. Experimental evidence has recently come from Okeke (2023), who shows that the marginal addition of doctors in primary care services in Nigeria significantly improves health outcomes, while lower-qualified health workers do not have any significant effects. Assessments of the introduction of PMM in Brazil provide quasi-experimental evidence and have gained academic attention both in economics and public health. Similar to other contexts, the country has long faced an unequal distribution of healthcare professionals, both in terms of quantity and quality (Costa et al., 2019). Despite increasingly dense, the literature on the effects of the introduction of PMM has yielded mixed results. For instance, Carrillo and Feres (2019) document that PMM led to the replacement of nurses by higher-qualified primary care doctors, without any effects on infant health. Fontes et al. (2018) and Mattos and Mazetto (2019) find that the introduction of PMM decreased hospitalizations, and Hone et al. (2020) document a small reduction in mortality amenable to primary care. Finally, our paper is also related to studies that quantify the value of medical personnel in producing health (Agha et al., 2019; Currie & Zhang, 2024; Fadlon & Van Parys, 2020; Ginja et al., 2025). For example, Currie and Zhang (2024) show meaningful differences in primary care provider performance within the U.S. Veterans Health Administration, while Ginja et al. (2025) document substantial heterogeneity in GP fixed effects in Norway.

We contribute novel evidence to the literature by exploiting a unique setting in which an exogenous shock to local health systems led to a large-scale exit of doctors at the market level. Importantly, although the diplomatic conflict that triggered the Cuban exit is unique, the local manifestation of the shock is similar to retention failures or abrupt staffing gaps that are common in resource-constrained health systems. Conditions such as high turnover among contract physicians, difficulty attracting doctors to remote areas, and sudden staffing gaps due to administrative or fiscal crises are recurring features of primary care provision in many low- and middle-income countries.

Unlike most studies that focus on increases in doctor supply, we assess the consequences of a sharp reduction in doctor availability. This is relevant because hirings and increases in the availability of doctors are generally followed by challenges in workforce retention, and positive and negative variations in doctor supply do not necessarily lead to symmetric effects on outcomes. Yet, while doctor retention and exit are a widespread policy challenge in all countries, evidence on the effects of doctor exit at the market level remains scarce. Existing studies tend to focus on specific disruptions in patient-provider relationships within developed countries,^1^ and often overlook effects beyond the directly affected patients and the broader market-level responses. In this paper, we not only examine the effects of a negative shock to the supply of doctors on population health outcomes at the market-level, but also investigate the response in terms of health production inputs and outputs. Our results are particularly informative for resource-scarce contexts, such as in low- and middle-income countries, where patient access to services and provider choice is typically more constrained, and therefore a negative shock to the supply of doctors may potentially lead to the most harmful health effects. To the best of our knowledge, this is also where causal empirical evidence on doctor exit is still absent.

This paper is organized as follows. In Section 2 we describe the institutional background. In Section 3 we detail the data, while in Section 4 we describe our empirical model and discuss identification. Results are presented in Section 5. In Section 6 we discuss potential pathways and explanations behind the results. Section 7 concludes.

## 2 Background

### 2.1 The Brazilian Health System and Primary Care Services

The Brazilian Unified Health System (*Sistema Único de Saúde*, SUS) is a tax-funded public healthcare system that provides healthcare services that are free-of-charge at the point of care at all levels of care. Although there exists private provision of health insurance and health services in Brazil, approximately 75% of the Brazilian population relies exclusively on SUS for healthcare (Rocha et al., 2021).

SUS has successfully expanded access to healthcare and improved health outcomes in Brazil (Castro et al., 2019). The Family Health Program (FHP), implemented by municipal governments and now covering over 60% of the population, has been key to that success (Bhalotra et al., 2019; Mrejen et al., 2021). The program provides preventive and curative primary care services through Family Health Teams (FHTs). Each team is based in a primary healthcare facility, called Basic Health Unit (*Unidade Básica de Saúde*, UBS), and typically consists of at least one doctor, nurses, community health workers and technicians. Each FHT covers around 3,500 individuals from a specific catchment area, and should respond to most of the population’ needs by providing curative care, health education, health promotion, disease prevention, less complex medical procedures, and referencing the more severe cases to specialized secondary or tertiary healthcare services when needed.

### 2.2 The More Doctors Program

Despite increasing numbers of doctors over the last decades, the supply of doctors and access to medical services and specialist care remain major bottlenecks for SUS in many regions, resulting in unmet demand, long waiting times, and delays in diagnoses (Castro et al., 2019). Brazil has long suffered from a highly unequal provision of doctors across municipalities, with urban peripheries and remote areas severely under-served. Making an effort to increase the supply of doctors in those regions, in 2013 the Brazilian government introduced the More Doctors Program (*Programa Mais Medicos*, PMM). The program was designed to pay competitive wages and provide additional benefits to doctors who agreed to work in eligible municipalities. Municipalities’ eligibility to take part in the PMM program was based on a range of different socio-demographic indicators defined by the federal government. These include poverty and extreme-poverty rates, the Human Development Index, semi-arid status, the presence of Quilombola communities, population size, and income per capita (Hone et al., 2020).

At the program’s inception, the government offered PMM positions to all Brazilian doctors. As low numbers of Brazilian doctors applied to fill the vacancies, the program was opened to doctors trained abroad. Through an international agreement between the governments of Cuba and Brazil, facilitated and managed by the Pan American Health Organization, a substantial number of Cuban doctors were sent to Brazil (Santos et al., 2017). Out of around 18,000 doctors that enrolled in the program between 2013 and 2018, roughly half came from Cuba. Whereas Brazilian doctors had priority and were able to freely choose their destination, Cubans were assigned to vacant positions and were seen as the last resort to fill PMM vacancies. Moreover, Brazilian doctors could work at multiple municipalities at the same time, whereas Cuban doctors were only allowed to work in one municipality. Given that Brazilian PMM doctors had a strong preference for urban centers, the Cuban doctors were essential for filling vacancies in poorer municipalities and remote areas (Santos et al., 2018).

In some participating municipalities, PMM doctors work alongside non-PMM primary care doctors. But the work schedules of PMM and non-PMM doctors differ in important ways. First, PMM doctors are only allowed to work in one primary healthcare unit, while non-PMM doctors are allowed to work in multiple units at the same time, also across municipalities (Santos et al., 2017). Second, PMM doctors are closely monitored and work a set of fixed 40 weekly hours, which makes them more accessible and reliable than non-PMM doctors (Comes, Trindade, Shimizu, et al., 2016). Finally, qualitative evidence suggests that PMM doctors play a crucial role in delivering primary care to Brazil’s poorest population. Cuban doctors in particular were often integrated into the local community, and most of them decided to apply for an extension of their stay in 2017 (Comes, Trindade, Pessoa, et al., 2016; Rech et al., 2018).

Cuba’s participation in PMM was highly politicized. One fierce opponent was Brazil’s former president, Jair Bolsonaro, who claimed the true goal of PMM was the installation of a foreign communist guerrilla in Brazil (Terra, 2019). Following his election in October 2018, the Cuban government unilaterally and unexpectedly decided to withdraw all of its 8,316 primary care doctors from PMM. As a consequence, Cuban doctors had merely a month to leave the country, and all consequently ceased to work before Bolsonaro took office in January 2019 (Santos et al., 2018). According to the media and policy makers, the decision came as a shock for PMM doctors and the involved institutions, given that the international contract had been extended only a year before (Sobrinho, 2018).^2^ Given the importance of the Cuban doctors for healthcare delivery, the media and academic community raised strong concerns about the potential adverse effects on health outcomes (Alves, 2018). The Brazilian government put great effort and expenses into trying to replace the missing primary care doctors, managing to fill almost all resulting PMM vacancies after a few months (Maffioli et al., 2019).

## 3 Data

### 3.1 Data Sources and Main Variables

We use different data sources to combine information on the number of doctors, healthcare utilization and inputs, subsidized prescription drug sales, and health outcomes at the municipality-by-month level. We cover the period from October 2017 to the end of December 2019. We end our analysis in December 2019, a year after the Cuban exit, just before the Covid-19 crisis. We detail below the main variables used in our analysis and their respective sources of data.

#### Data on doctors and PMM

We use data on municipalities’ PMM participation from the Brazilian Ministry of Health. The data set contains PMM eligibility and the number of PMM doctors (Cuban and others) registered in each municipality, for all Brazilian municipalities in a given month. We limit our sample to municipalities that are eligible to participate in PMM. Excluding non-eligible municipalities, which are typically Brazil’s most developed ones, makes our main sample more homogeneous.^3^ Out of all 5,570 municipalities in Brazil, 4,162 participated in PMM.^4^ Given this sample, we divide PMM municipalities into three groups, based on the relevance that Cubans played for the local PMM in October 2018, the month just before the Cuban withdrawal. Municipalities with no Cuban PMM doctors in October 2018 form our control group (*N* = 1, 320). Municipalities with Cuban PMM doctors, but also non-Cuban PMM doctors form our first treatment group (*N* = 1, 260). This group lost, on average, around 50% of their PMM doctors with the Cuban exit. Municipalities with only Cuban PMM doctors form our second treatment group (*N* = 1, 583). Having temporarily lost all their PMM doctors with the exit, we expect this treatment group to be most strongly affected by the shock.

We complement the PMM data with information on the total monthly number of primary healthcare doctors per municipality (PMM and non-PMM doctors) from the National Registry of Health Facilities (*Cadastro Nacional de Estabelecimentos de Saúde*, CNES). CNES contains information on all Brazilian healthcare facilities, healthcare workers enrolled in each facility, and their weekly working hours. A limitation of the CNES data is that it is not well suited to capture high frequency changes. Even though data is available on a monthly level, information is updated irregularly (facilities need to update their employment numbers only at least every 6 months). Hence, CNES may not fully capture monthly changes and will be used as an auxiliary source of information. Moreover, the CNES data set does not indicate whether primary care doctors are enrolled in PMM or not. To distinguish between PMM doctors and non-PMM doctors in the CNES dataset, we merge the PMM dataset with CNES through the names and municipality work location of the doctors.^5^

#### Primary healthcare production and utilization

We obtain data on primary healthcare production and utilization from the National Information System on Primary healthcare (*Sistema de Informação Atenção Básica*, SISAB). The system registers all primary care services provided in a given municipality in a given month and funded by SUS, including information on the nature of the service and the responsible healthcare professional. The system allocates healthcare services into four overarching categories: consultations, procedures, dental care, and home visits. Out of these four, consultations are predominantly done by doctors, while the others are mostly delivered by other healthcare workers. Primary care consultations, i.e., doctors’ main duties, are further divided into the type of appointment (spontaneous, scheduled one time, scheduled on a regular basis, etc.) and into specific conditions (diabetes, hypertension, tuberculosis, obesity, etc.). For the years 2022 to 2024, SISAB also records primary healthcare quality indicators that measure whether patients with hypertension or diabetes received at least one consultation and the recommended basic follow-up tests in the past semester. Tables A.1 and A.2 provide the details.

We collect data on primary care utilization related to asthma, diabetes, hypertension, cardiovascular diseases, cancers, chronic obstructive pulmonary disease (COPD), and mental health as chronic conditions. Related risk factors are malnutrition, obesity, rehabilitation, and consultations concerning tobacco, alcohol, and drug use. Maternal health is concerned with the full cycle of pre- and postpartum. Dengue, leprosy, tuberculosis, and sexually transmitted diseases cover outcomes related to infectious conditions in the data.

We also use data on ambulatory care production from the National Information System of Ambulatory Care (*Sistema de Informação Ambulatorial*, SIA). This data set contains information on ambulatory care costs and production by number and type of procedures which are funded by SUS and delivered at different providers, per municipality and month. These data allow us to distinguish between outpatient care provided at emergency rooms, hospitals or specialty centers.

#### Prescription drugs and private insurance coverage

We obtain data on prescription drug sales from Brazil’s large-scale subsidizing program of prescription drugs (*Aqui Tem Farmácia Popular*, ATFP). The program covers sales of medication for different conditions such as diabetes and hypertension, which are handed out to patients at strongly subsidized prices or for free.^6^ The data cover the amount and value of prescription drugs handed out by the 24,336 pharmacies enrolled in ATFP, for different conditions, by month and year from Brazil’s Ministry of Health. To get access to the subsidized medication, patients need to have a medical prescription, which is valid for up to 180 days (Américo & Rocha, 2020). Both the PMM and the ATFP program aim at ensuring accessible and affordable healthcare for everyone. In 2019, the ATFP program reached a substantial share of patients suffering from conditions that are covered by the program —around 50% of Brazilian patients who recently took medication for diabetes or hypertension obtained at least some of them via ATFP (Américo & Rocha, 2020). We use quarterly data on private insurance coverage at the municipality level, originally from Brazil’s National Agency for Supplementary Health (ANS).

#### Hospitalization and mortality

We obtain microdata from the National System of Information on Hospitalizations (*Sistema de Informação Hospitalar*, SIH), which includes all hospitalizations publicly funded by SUS disaggregated by primary cause of admission (based on ICD-10 code; International Classification of Diseases 10th Revision) and the patients’ municipality of residence. We also obtain microdata from the National System of Information on Mortality (*Sistema de Informação de Mortalidade*, SIM), which includes official registries of all deaths recorded in Brazil. The microdata include the municipality of residence and the ICD-10 code for the main cause of death.^7^

#### Socioeconomic characteristics

We use annual municipal socioeconomic characteristics as control variables in auxiliary exercises. We include the number of families receiving the Bolsa Familia social welfare transfers per 100,000 residents, and the municipal costs of Bolsa Familia per resident (data from the Ministry of Social Development, MDS),^8^ the municipal GDP per capita, the value added by public sector employment to the local economy, the share of the population above the age of 65, the sex ratio, the size of the municipal population, and the municipality’s population density (data from IBGE).

### 3.2 Descriptive Statistics

Figure A.1 illustrates the spatial distribution of municipalities according to their treatment status. White municipalities do not participate in PMM and were excluded from our main analysis. Light grey municipalities are our control group (municipalities without any PMM Cuban doctors), dark grey municipalities represent treatment group one (municipalities with some PMM Cuban doctors), and black municipalities form treatment group two (all PMM doctors were Cuban). There is no discernible geographical pattern with respect to the allocation of municipalities into the different groups. Table A.3 summarizes how the different treatment groups are dispersed across the Brazilian macro-regions.

Table A.4 summarizes all outcome variables investigated in our analyses, per 100,000 residents in October 2018 (the baseline month), including doctor supply, healthcare utilization and health outcome variables. The average control municipality employed 11 PMM doctors per 100,000 residents, as compared to 18 and 20 in our two treatment groups. In our first treatment group, 52% of PMM doctors were Cuban, which is equivalent to about 21% of all primary care doctors working in public primary care facilities.^9^ In municipalities in treatment group two, the Cubans, and, by extension, PMM doctors, represented approximately 31% of all primary care doctors working in primary care facilities.

Primary care doctors were responsible for 64% of the primary care consultations during the pre-treatment period, but only for 4% of the ambulatory procedures and for 0% of home visits and dental care services. Yet, while consultations in general are doctors’ main duties, some of them are carried out by other health workers, depending on the specific condition.^10^ For descriptive statistics on the auxiliary socioeconomic variables, see Table A.5.

### 3.3 Trends in PMM Coverage

**Figure 1:**
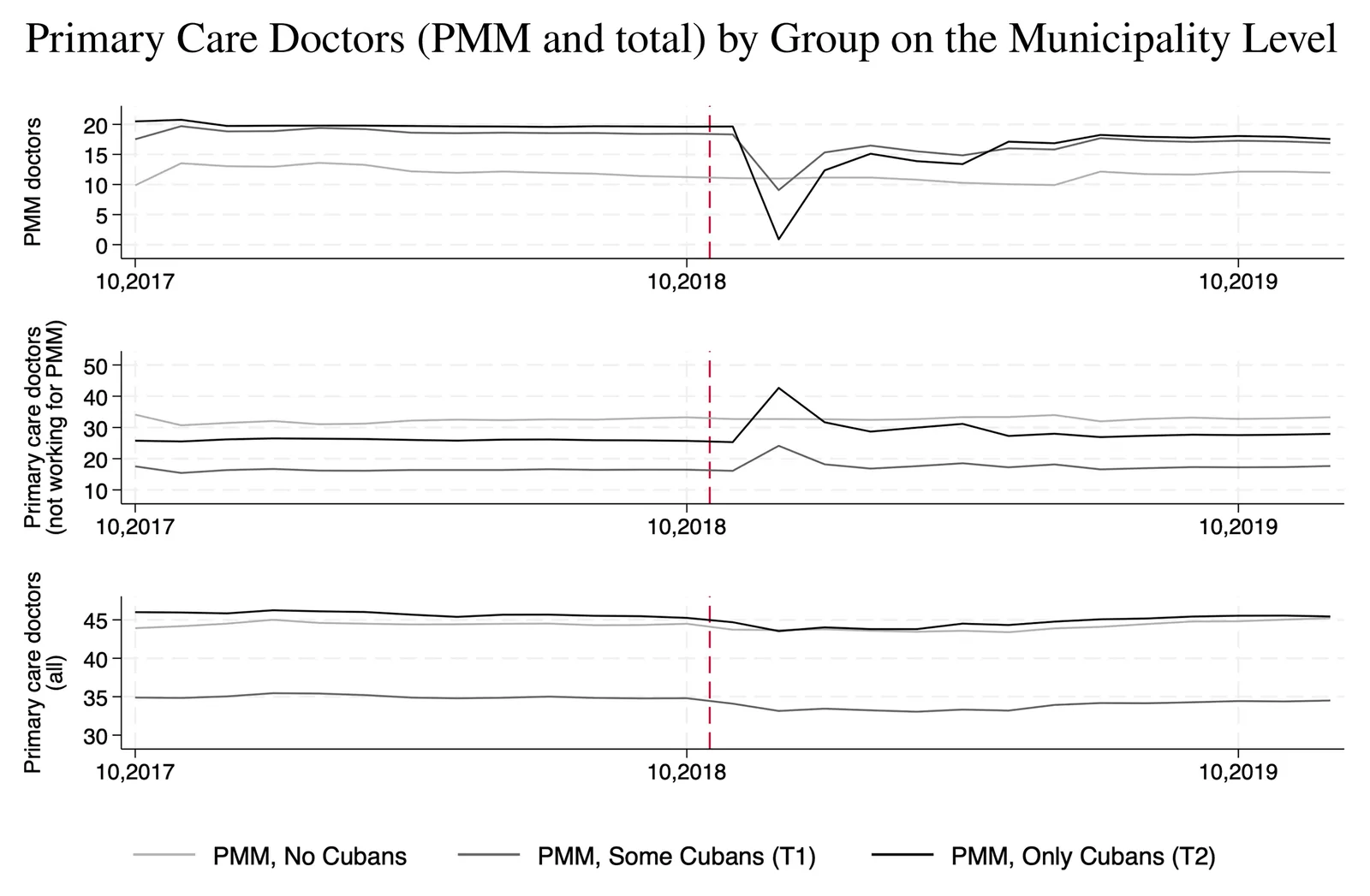
The graph shows trends in the group means of the number of PMM doctors (upper plot), non-PMM primary care doctors (middle plot), and all primary care doctors (lower plot) per 100,000 residents. The series in dark grey presents treatment group one, the series in black presents treatment group two. The dashed vertical lines present the pre-treatment month October 2018. Original data from the Brazilian Ministry of Health and CNES.

How did the Cuban exit affect the supply of primary healthcare doctors? Figure 1 shows the monthly unconditional means of the number of PMM doctors (upper plot), of the number of primary healthcare doctors not affiliated with PMM (middle plot), and of the number of all primary healthcare doctors (PMM and non-PMM, lower plot) per 100,000 residents by treatment group. In general, the unconditional means move similarly throughout the groups before the treatment. The Cuban exit clearly marks a recess in the trends for affected municipalities. When examining PMM doctors, we observe a sharp drop in coverage for treatment groups one (dark gray) and two (black), with treatment group two being more strongly affected. While in treatment group one, the number of PMM doctors is halved, the second treatment group briefly loses all PMM doctors. Importantly, there is not any clear inflection in the control group (light gray). Within a few months after the shock, PMM coverage in treatment municipalities recovers and almost returns to pre-treatment levels.

Although less pronounced, a similar pattern can be observed when looking at overall primary healthcare doctors (lower plot). The treatment groups experience a drop in coverage, followed by a recovery a few months after the shock. The drop, however, is substantially smaller than the drop in PMM doctors, which can be explained by two factors. First, there was an expansion in primary healthcare doctors not affiliated with PMM after the Cuban exit which partially cushioned the Cuban exit (middle plot). Second, as mentioned in Section 3.1, even though CNES data is available at the monthly level, records are not immediately updated as facilities need to update their employment numbers only at least every 6 months, therefore smoothing aggregated trends.

## 4 Empirical Model

We exploit the sharp and exogenous timing of the Cuban withdrawal from PMM in municipalities that relied more *versus* less on Cuban PMM doctors at the time of withdrawal to estimate the effects of doctor exit on healthcare utilization, system adaptation, and health outcomes. Our empirical approach compares changes in outcomes between affected and unaffected PMM municipalities before and after the Cuban exit, while flexibly controlling for time-invariant municipal characteristics and common time shocks.

Given that our outcomes are non-negative counts or rates (e.g., numbers of consultations, hospitalizations, or deaths per 100,000 residents), many of which include zeros, our preferred modeling choice is to estimate all specifications using the Poisson pseudo-maximum likelihood (PPML) estimator. PPML is well suited for our context because it does not impose distributional assumptions on the dependent variable beyond correct specification of the conditional mean, remains consistent under arbitrary forms of heteroskedasticity, and naturally accommodates zero values. This avoids the well-known biases associated with log or inverse hyperbolic sine transformations when outcomes include zeros (J. Chen & Roth, 2024). In addition, PPML performs reliably in high-dimensional fixed-effects settings (Correia et al., 2020), making it appropriate for our event-study design with municipality fixed effects and state-by-month fixed effects. For a PMM municipality *i* at year-month *t*, we therefore estimate a PPML event-study where the specification of the conditional mean follows:

(1) $$E\left[Y_{it}|X_{it}\right]=exp\left(\alpha_{i}+\phi_{st}+\underset{g}{\sum }\left[\sum_{t=-13}^{-2}\beta_{gt}\left(D_{i}^{g}\times T_{t}\right)+\sum_{t=0}^{14}\beta_{gt}\left(D_{i}^{g}\times T_{t}\right)\right]\right)$$

Where *Y*_*it*_ refers to outcomes of interest. The term $D_{i}^{g}$ is our categorical treatment variable and takes $D_{i}^{g=1}=1$ if the municipality had some Cuban doctors, and $D_{i}^{g=2}=1$ if it had only Cuban doctors in PMM in October 2018, the month before the Cuban exit. Municipalities are assigned to the control group otherwise.

The first summation set captures the coefficients of the interactions between treatment status and year-month dummies *T*_*t*_ before October 2018, while the second refers to coefficients after the shock. We include municipality fixed effects *α*_*i*_ to control for fixed characteristics and baseline heterogeneity across municipalities, including municipalities’ baseline socio-demographics that determine program eligibility and can be considered highly persistent, therefore fixed over our short pre-period of analysis. As we focus on a relatively high frequency data just around the shock (i.e., monthly variation within approximately a 2-year period), the term *α*_*i*_ is also expected to absorb relevant slow-moving determinants of health, such as the presence of hospitals and transport connections. Although we focus on a short period of time, we further consider interactions between time and state fixed effects *ϕ*_*st*_, therefore allowing for flexible time trends per state. This is expected to absorb the influence of state policies, particularly relevant in healthcare as states are typically responsible for secondary and tertiary health services. We cluster standard errors at the municipality level to allow for correlation of the error term within the same municipality throughout time.

We assume that the variation in the interaction between the sharp and unexpected exit of Cuban doctors (time-series variation) and the baseline variation in the presence of Cuban doctors (across municipalities) is exogenous to municipalities, conditional on fixed effects. Identification relies on the assumption that trends in outcomes in both treatment groups would not have differed from the observed trend in the control group should Cuban doctors had not left PMM. We investigate the parallel trends assumption by looking at monthly differences in outcomes between control and treatment groups throughout the year before the Cuban exit. More specifically, we visually inspect and formally test whether the coefficients *β*_−13_ (October 2017) to *β*_−2_ (September 2018) portray any systematic pattern. The overall evidence, across outcome variables, indicates that pre-trends remain irrelevant.^11^

We also present results from alternative specifications and treatment definitions. First, instead of considering treatment as categorical groups interacted with time fixed effects, we use the share of Cuban PMM doctors among all local primary care doctors at baseline interacted with time fixed effects, as follows:

(2) $$E\left[Y_{it}|X_{it}\right]=exp\left(\alpha_{i}+\phi_{st}+\sum_{t=-13}^{-2}\beta_{gt}^{*}\left(S_{i}\times T_{t}\right)+\sum_{t=0}^{14}\beta_{gt}^{*}\left(S_{i}\times T_{t}\right)\right)$$

Where *S*_*i*_ refers to the share of Cuban PMM doctors in municipality *i* in September 2018 among all local primary care doctors (PMM and non-PMM). In this case, we must rely on a more stringent assumption of parallel trends along the distribution of *S*_*i*_. This specification nevertheless comes with the benefit of estimating average effects across all PMM municipalities rather than splitting the estimates for different treatment groups. Finally, although we rely on a high frequency panel of data, in additional robustness checks, we include time-varying controls to inspect the stability of our estimates. We show that results remain stable to adjusting for municipality annual GDP per capita, population sex and age structure, and social welfare transfers.

## 5 Doctor Exit, Health Utilization and Outcomes

### 5.1 Effects on the Supply of Doctors

**Figure 2:**
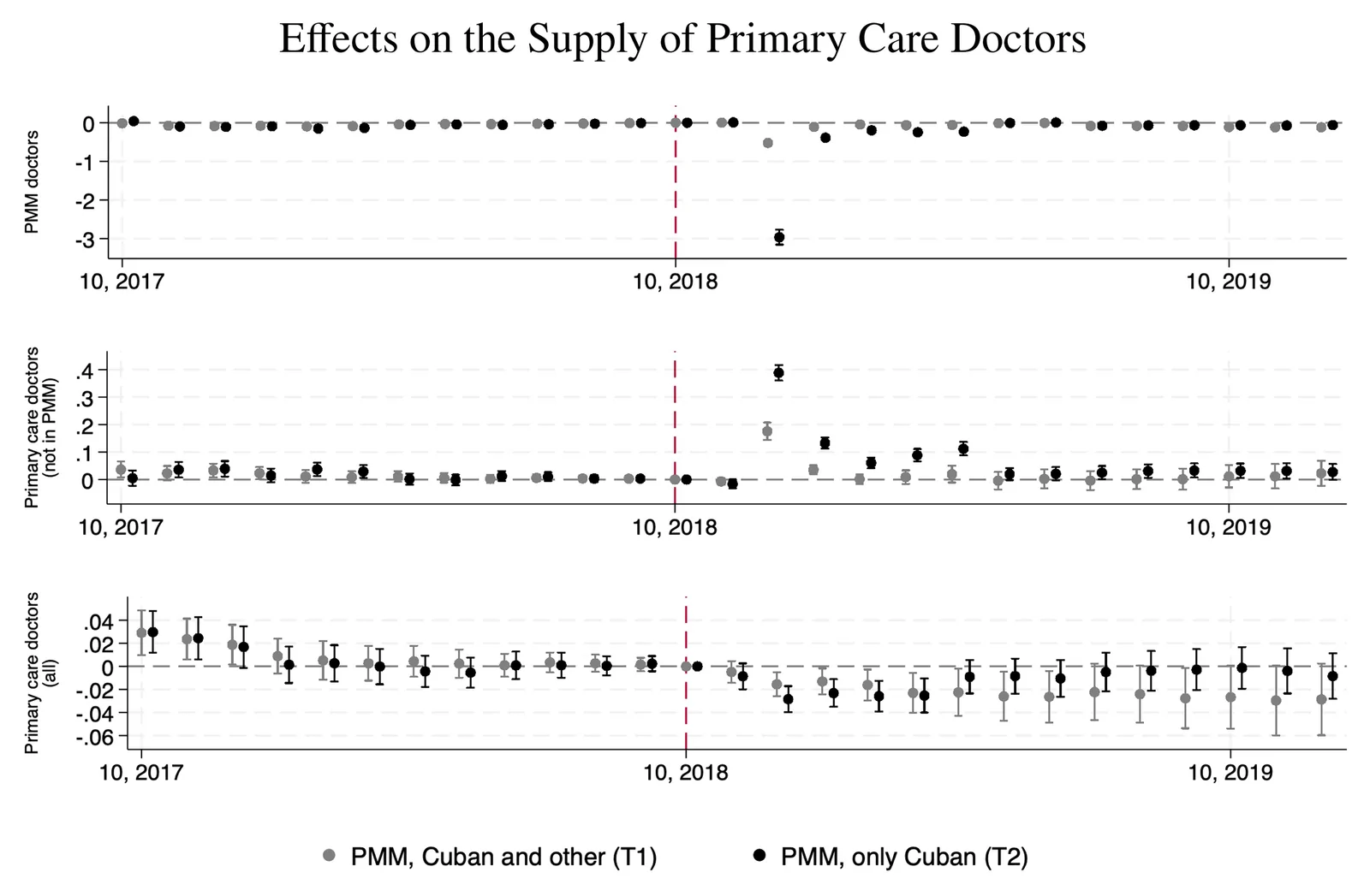
The graph shows event studies for the number of PMM doctors (upper plot), non-PMM primary care doctors (medium plot), and all primary care doctors (lower plot), following equation 1. Regressions include municipality and state-by-time (month) fixed effects. Dependent variable adjusted by population size. The series in grey presents treatment group one, the series in black presents treatment group two. Whiskers around the point estimates present 95 percent confidence intervals, where standard errors are clustered at the municipality level. The dashed vertical lines present the pre-treatment month October 2018. Joint p-value of pre-treatment coefficients in treatment group one (two): PMM doctors: 0.00 (0.00). PC doctors (not in PMM): 0.06 (0.00). PC doctors (all): 0.20 (0.00). Original data from the Ministry of Health and CNES.

We start our empirical analysis by investigating the effect of the Cuban exit on the supply of primary care doctors in affected municipalities. Figure 2 shows event studies for the number of PMM doctors, the number of non-PMM doctors (i.e., primary healthcare doctors not affiliated with PMM), and the number of all primary care doctors (PMM and non-PMM) per 100,000 residents.

We observe a steep reduction in the supply of PMM doctors, especially for treatment group two. In December 2018, at the height of the impact, PMM coverage decreased, on average, by 41% in municipalities of treatment group one, and by 95% in municipalities of treatment group two.^12^ This corresponds to 8 fewer PMM doctors per 100,000 residents in treatment group one (relative to a group average of 18 PMM doctors per 100,000 residents in October 2018) and 19 fewer PMM doctors per 100,000 residents in treatment group two (relative to a group average of 20 PMM doctors per 100,000 residents in October 2018).^13^ The supply of PMM doctors recovered for both treatment groups about 6 to 8 months after the Cuban exit, with treatment group one filling vacancies faster than treatment group two. While the reduction in PMM coverage remains significant over time, the difference is small in absolute terms and in comparison to pre-treatment levels.

Examining non-PMM primary healthcare doctors shows there is an expansion in their supply, seemingly as an immediate reaction to the Cuban exit. For treatment groups one and two, there is an increase of 19% and 47% of non-PMM primary healthcare doctors in December 2018, corresponding on average to 3 and 13 doctors per 100,000 residents, respectively. These numbers halve in 2019 as PMM vacancies are filled, but remain elevated throughout our sample period.

Finally, looking at all municipal primary care doctors (PMM and non-PMM), we see a small but significant drop during the supply shock period. Despite the expansion of non-PMM doctors, the number of all primary care doctors decreased, on average, by 1.5% in treatment group one and 2.8% in treatment group two in December 2018, each corresponding to roughly one primary care doctor per 100,000 residents. These effects can be interpreted as a lower bound as CNES records do not accurately respond to monthly changes in actual figures. We also observe a decaying tendency in the number of all primary healthcare doctors by the turn of the 2017-2018 years. This trend is nevertheless smooth and pre-trends stabilize for most of 2018, remaining systematically around zero in the months before the Cuban exit.

### 5.2 Effects on Primary Care Utilization

**Figure 3:**
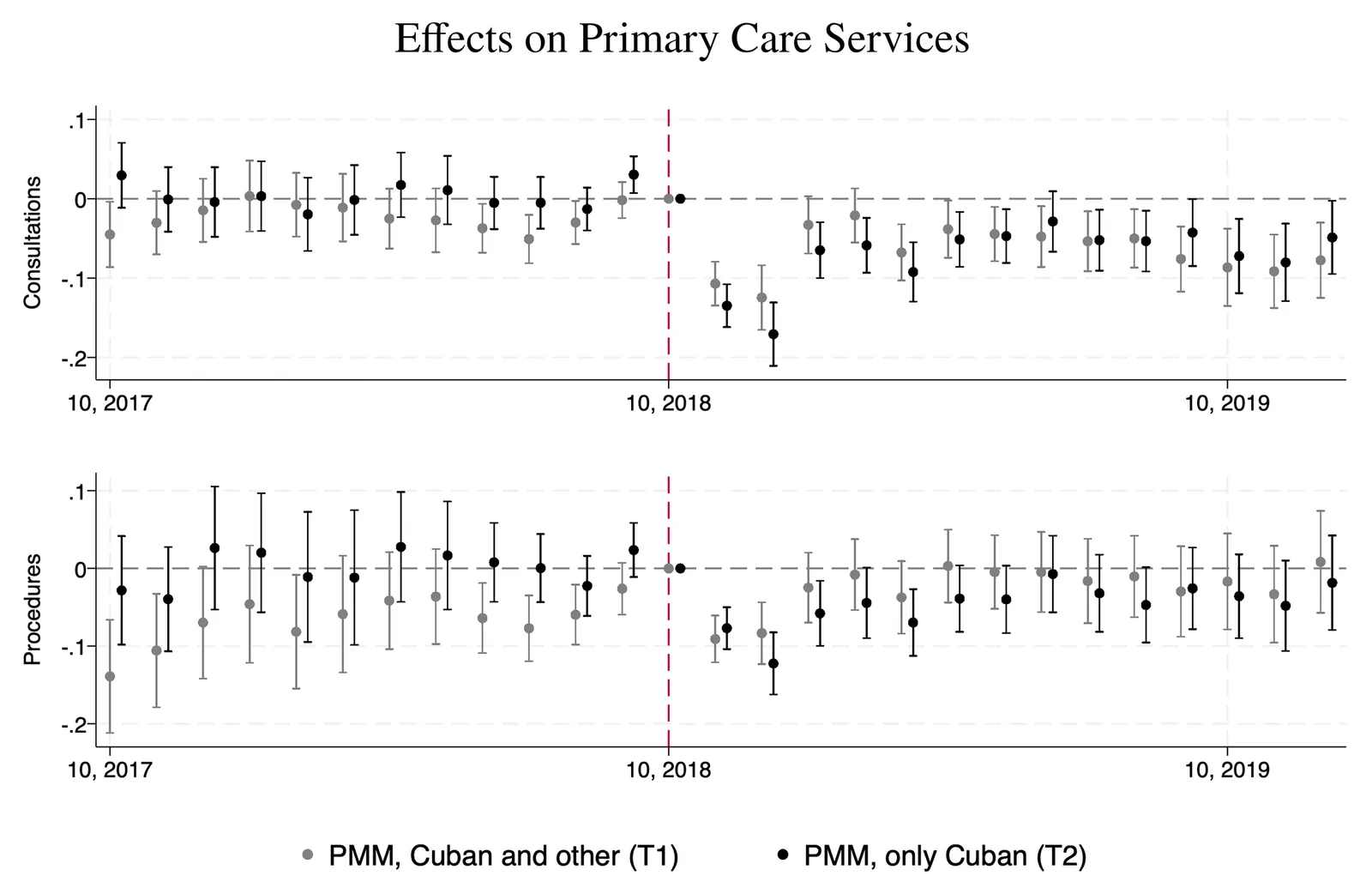
The graph shows event studies for the number of consultations (upper plot) and procedures (lower plot) performed in primary healthcare facilities, following equation 1. Regressions include municipality and state-by-time (month) fixed effects. Dependent variable measured per 100,000 inhabitants. The series in grey presents treatment group one, the series in black presents treatment group two. Whiskers around the point estimates present 95 percent confidence intervals, where standard errors are clustered at the municipality level. The dashed vertical lines present the pre-treatment month October 2018. Joint p-value of pre-treatment coefficients in treatment group one (two): Consultations: 0.03 (0.01). Procedures: 0.00 (0.03). Original data from SISAB.

Given the reduction in the supply of primary care doctors, we expect a concurrent drop in primary care utilization related to consultations, which are doctors’ main duties. Figure 3 shows a 12% reduction in consultations in treatment group one, and a 16% drop in treatment group two in December 2018. The decline corresponds, on average, to 1,366 and 2,540 fewer monthly consultations per 100,000 residents in treatment groups one and two, respectively. The negative effect largely recovers as vacancies are filled with new PMM doctors. Recovery is quicker in treatment group one, but a negative effect of 7% and 5% persists in treatment groups one and two until the end of 2019, corresponding to roughly 817 and 809 fewer consultations per 100,000 residents. Figure 3 also shows that effects are greater and more persistent for primary care consultations, which are doctors’ main duties. We observe only transitory effects for tasks that are mostly performed by nurses and other healthcare workers in primary healthcare centers, such as minor procedures.

**Figure 4:**
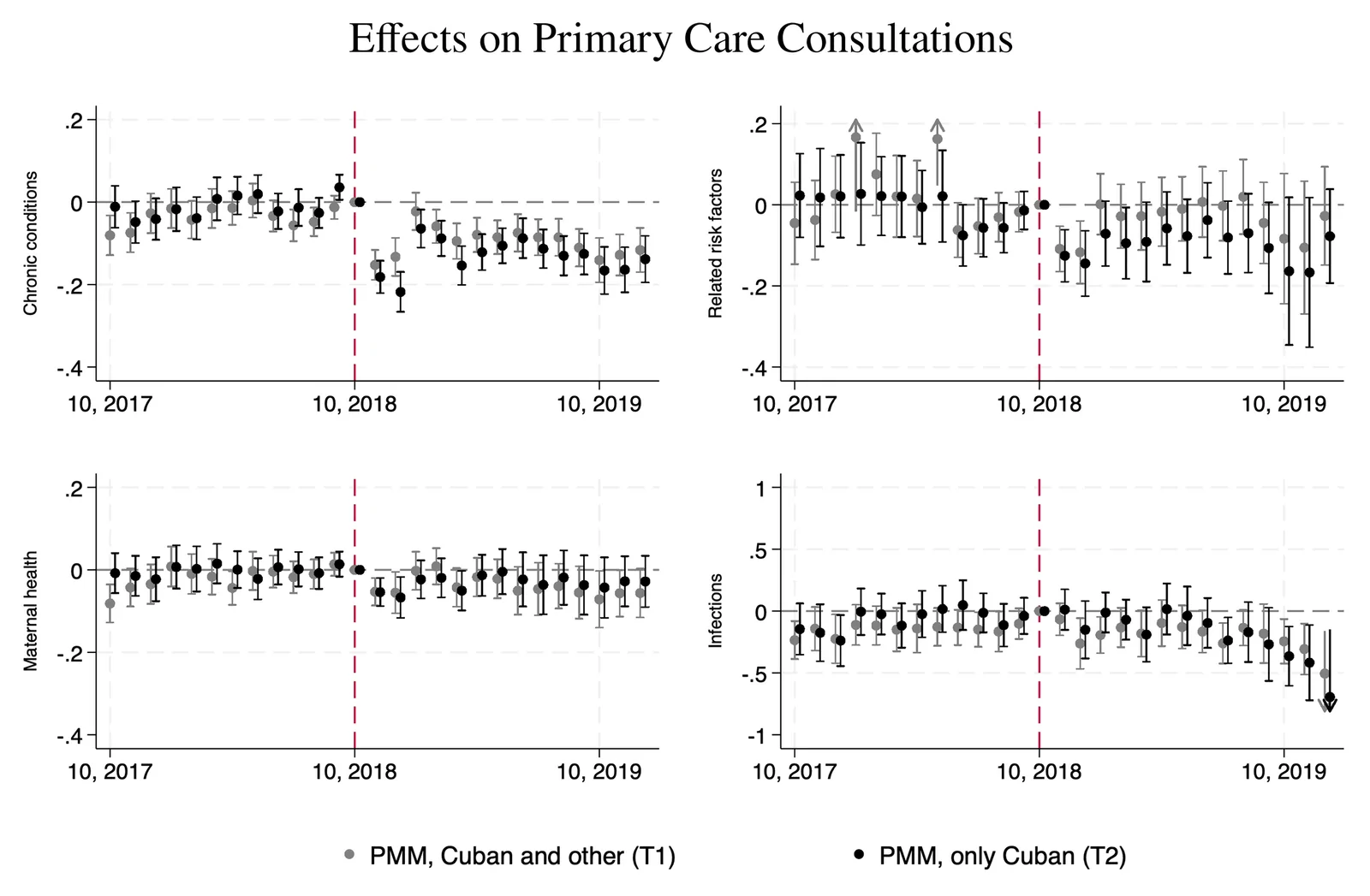
The graph shows event studies for the number of consultations performed in primary healthcare facilities in four different primary care categories, following equation 1. Chronic conditions include asthma, diabetes, hypertension, cardiovascular diseases, cancers, COPD and mental health. Related risk factors include malnutrition, obesity, rehabilitation and the use of drugs (tobacco, alcohol and other drugs). Maternal health includes the full cycle of pre- and postpartum. Infectious diseases include dengue, leprosy, tuberculosis and STDs. Regressions include municipality and state-by-time (month) fixed effects. Dependent variable measured per 100,000 inhabitants. The series in grey presents treatment group one, the series in black presents treatment group two. Whiskers around the point estimates present 95 percent confidence intervals, where standard errors are clustered at the municipality level. The dashed vertical lines present the pre-treatment month October 2018. Joint p-value of pre-treatment coefficients in treatment group one (two): Chronic conditions: 0.00 (0.00). Related risk factors: 0.00 (0.50). Maternal health: 0.00 (0.81). Infections: 0.32 (0.28). Original data from SISAB.

When unpacking consultations further, we see that not all primary care needs are affected in the same way. In Figure 4, we distinguish between primary care consultations on chronic conditions, related risk factors to chronic conditions, maternal health, and infections. The effects on chronic conditions and related risk factors are strong and persistent, especially for treatment group two. This group experiences a drop of roughly 20% for chronic care (around 890 per 100,000) and 13% for related risk factors (around 225 per 100,000) in December 2018, and this decrease persists in magnitude until the end of our sample period. Consultations on maternal health are only temporarily affected and fully recover over time. For example, there were on average around 6% (around 189 per 100,000) fewer maternal health consultations for treatment group two in December 2018, but the trends were indistinguishable from zero for most of 2019. Consultations on infectious diseases experience a short-term reduction, followed by recovery, with some significant effects towards the end of our study period.

Figures B.1, B.2 and B.3 show results on specific diseases and confirm the overall picture. We observe in Figures B.1 and B.2 that consultations on chronic conditions mostly experience a strong and persistent decrease. On the other hand, Figure B.3 shows that infectious diseases are generally less and only temporarily affected, even when doctors are more involved, such as for consultations on STDs.

In sum, there was a sharp decrease in primary care utilization immediately after the Cuban doctor exit. However, while primary care conditions requiring immediate care, such as maternal health and infections, recovered quickly, there was a strong and persistent decrease in the management and care of chronic conditions, especially in the most affected municipalities, where PMM relied entirely on Cuban doctors.

### 5.3 Hospitalizations and Mortality

**Figure 5:**
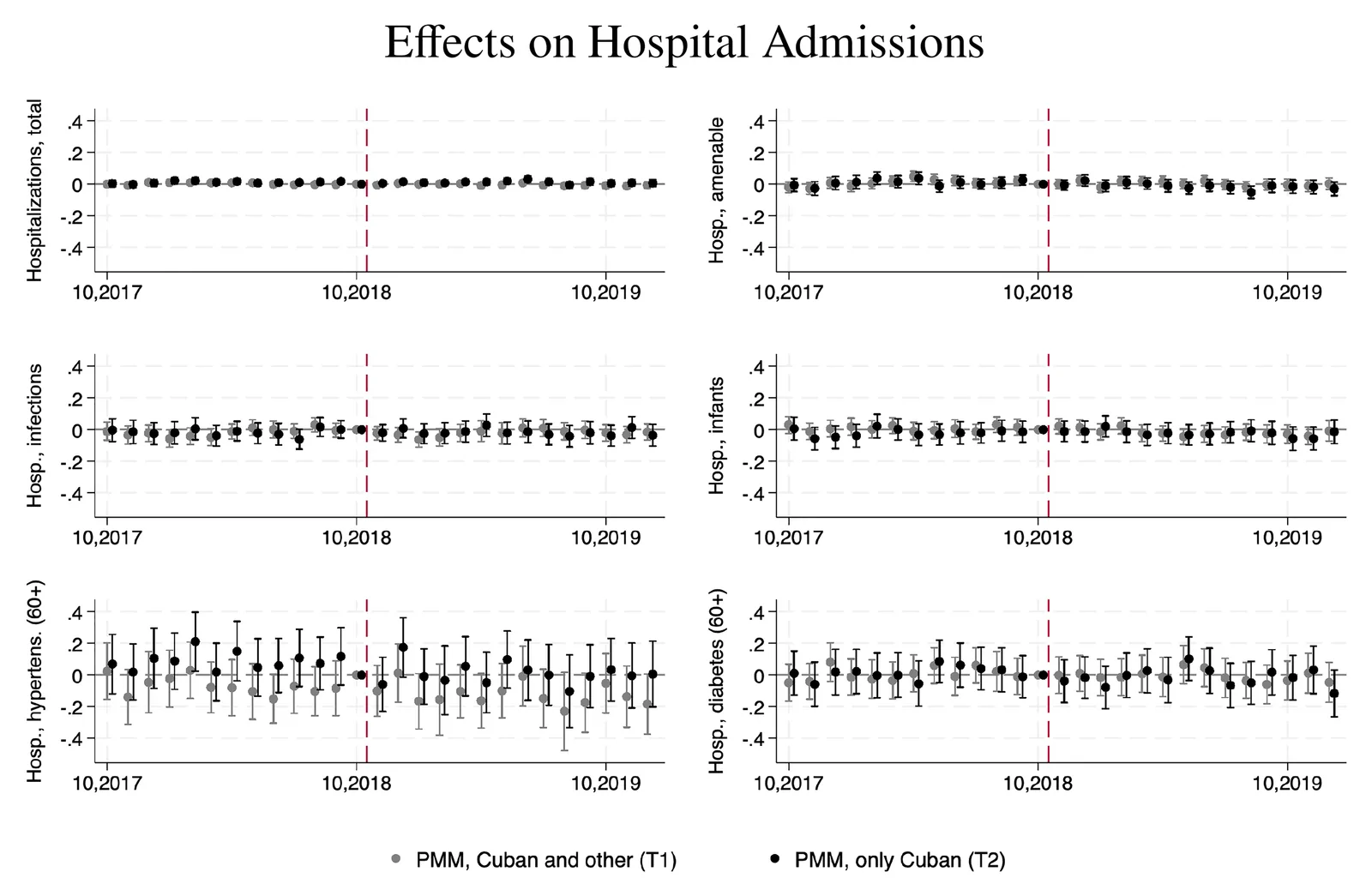
The graph shows event studies for the number of hospitalizations in six different categories, following equation 1. Where indicated, the number of hospitalizations only includes individuals aged 60 and above. Regressions include municipality and state-by-time (month) fixed effects. Dependent variable adjusted by population size. The series in grey presents treatment group one, the series in black presents treatment group two. Whiskers around the point estimates present 95 percent confidence intervals, where standard errors are clustered at the municipality level. The dashed vertical lines present the pre-treatment month October 2018. Joint p-value of pre-treatment coefficients in treatment group one (two): Total hospitalizations: 0.28 (0.18). Amenable: 0.01 (0.05). Infections: 0.27 (0.42). Infants: 0.56 (0.63). Hypertension (60+): 0.19 (0.63). Diabetes (60+): 0.36 (0.75). Original data from SIH/Datasus.

**Figure 6:**
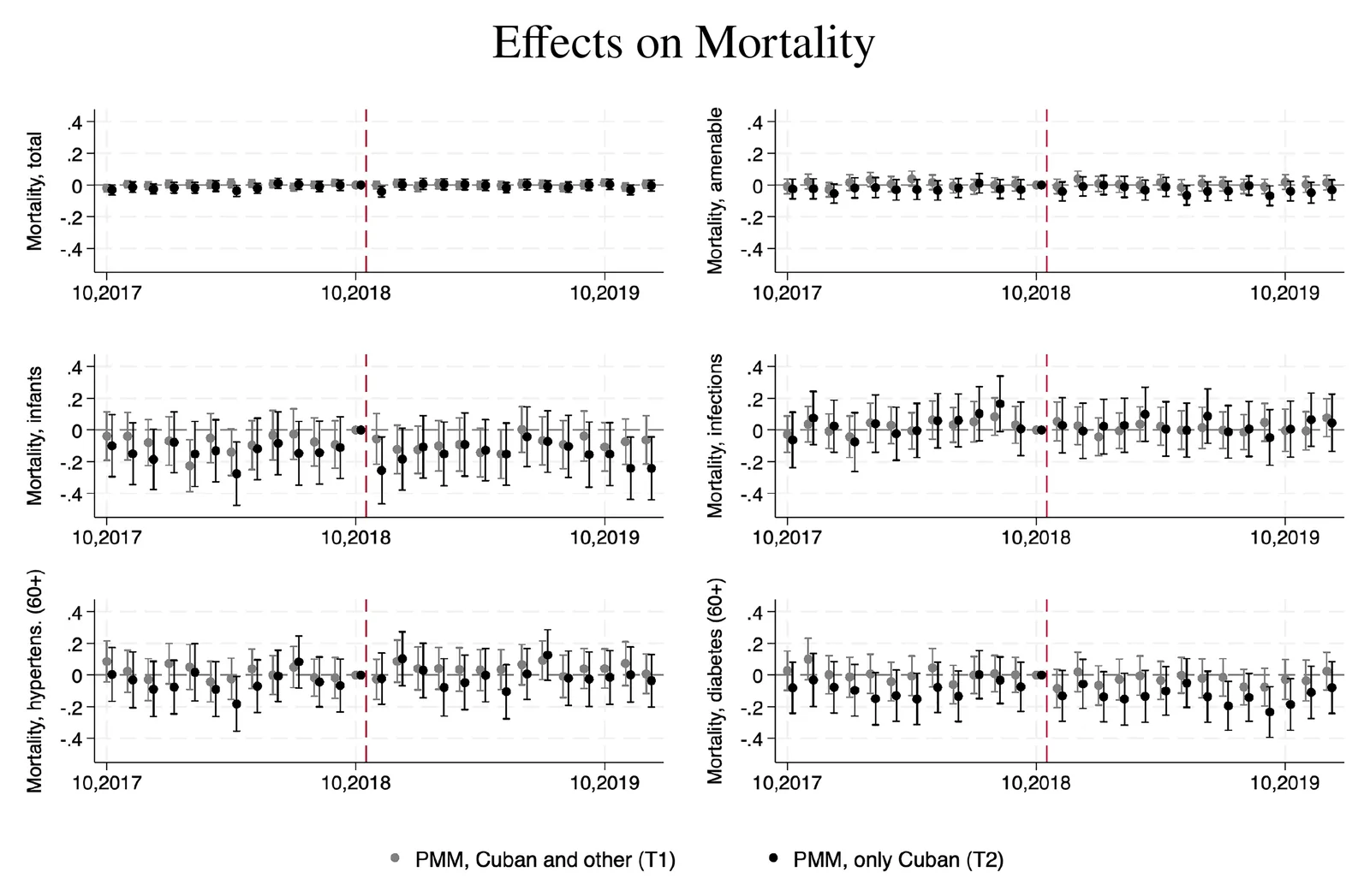
The graph shows event studies for the number of hospital mortality in six different categories, following equation 1. Where indicated, the number of hospital mortality only includes individuals aged 60 and above. Regressions include municipality and state-by-time (month) fixed effects. Dependent variable adjusted by population size. The series in grey presents treatment group one, the series in black presents treatment group two. Whiskers around the point estimates present 95 percent confidence intervals, where standard errors are clustered at the municipality level. The dashed vertical lines present the pre-treatment month October 2018. Joint p-value of pre-treatment coefficients in treatment group one (two): Total hospital mortality: 0.25 (0.17). Amenable: 0.30 (0.89). Infections: 0.66 (0.20). Infants: 0.33 (0.58). Hypertension (60+): 0.64 (0.29). Diabetes (60+): 0.74 (0.63). Original data from SIM/Datasus.

We now investigate effects on health outcomes. Figure 5 presents effects on total hospital admissions as well as by specific causes of admission. Figure 6 reports analogous results for mortality. We distinguish between total outcomes, outcomes amenable to primary care, and those specifically related to infections, infant health, and chronic conditions among the population aged 60 and above.^14^ We observe that estimates are close to zero and mostly insignificant, even for outcomes that are expected to be more sensitive to short-term changes in access to healthcare, such as infant hospitalizations or hospital admissions of causes amenable to primary care (Carrillo & Feres, 2019).^15^

For hospitalizations, the precision of the estimates is sufficient to rule out changes as large as those observed after the introduction of PMM. Based on the upper limit of the 95% confidence intervals, we can reject increases in total hospitalizations exceeding 5.1%, and all but one estimate exclude effect sizes of the magnitude reported by Mattos and Mazetto (2019). For hospitalizations amenable to primary care, we can reject increases exceeding 5.8%, thereby excluding effect sizes of the magnitude reported by Fontes et al. (2018).^16^

Mortality outcomes are estimated with more uncertainty, as expected, but results show a similar pattern. Effect estimates are centered near zero, with no systematic deviations across treatment groups or time. We can rule out increases in total mortality larger than 4.2% (2.3 deaths per 100,000 residents per month in treatment group two) and increases in amenable mortality larger than 6.7% (1.1 deaths per 100,000 residents per month in treatment group two). For rare mortality outcomes at the monthly level, such as infant mortality, the confidence intervals are wider, allowing us to rule out only relatively large effects, e.g., increases up to 15.9% in treatment group one. Yet, these correspond to very small absolute changes (0.26 deaths per 100,000 residents per month in treatment group two), and the point estimates again show no systematic evidence of deterioration.^17^

While effects on health outcomes may take a period of time to materialize (Hone et al., 2020; Özçelik et al., 2020), we observe that a sharp and non-trivial reduction in primary healthcare utilization does not translate into any systematic changes in health outcomes even throughout the end of the following year after the shock. To place these results in context, we compare our findings with the existing evidence on the introduction of PMM. The contractions in primary care utilization that we document mirror the increases observed after PMM’s rollout. Mattos and Mazetto (2019) report increases of 6–30% in primary care utilization, and Carrillo and Feres (2019) find increases of 5–8% in doctor visits. Our estimated declines during the turnover period lie within this range, indicating that the withdrawal produced a shock to service availability that is broadly symmetric to the initial gains. By contrast, our null effects on hospitalizations and mortality stand alongside more mixed evidence. While some papers find reductions in total hospitalizations (Fontes et al., 2018; Mattos & Mazetto, 2019) and amenable mortality Hone et al. (2020), others report more muted impacts, including for infant mortality Carrillo and Feres (2019). The absence of systematic deterioration in our setting suggests that contractions may not generate adverse effects of a magnitude comparable to improvements documented during program expansion. Taken together, these comparisons therefore point to a degree of asymmetry between expansions and contractions in doctor supply. We further discuss this result in Section 6. In particular, we examine whether and how demand and local health systems responded to the shock, potentially protecting and leaving health outcomes unaffected.

### 5.4 Alternative Specifications and Robustness Checks

In this section, we further discuss the robustness and the interpretations of our main results. We present robustness checks for our main results on primary care utilization (consultations, procedures and consultations by condition type) and on health outcomes (hospitalizations and mortality). We first test the stability of our estimates to the inclusion of time-varying controls. In our main specifications, we employ municipality and state-time fixed effects. Given the high frequency data within a relatively short period of analysis, and conditional upon municipality fixed effects, we expect the observed variation in the supply of doctors to be exogenous to municipality fixed characteristics as well as to slow-moving municipality trends. To test for this, in Section C.1 we replicate all our results now from specifications that include annually available socioeconomic control variables at the municipality level, such as the population share of Bolsa Familia coverage (the main social welfare program in Brazil), Bolsa Familia expenses per capita, GDP per capita, value added by public sector employment (in R$ 1,000), the share of the population above 65, and the sex ratio. In Figures C.1 to C.4, we observe that results remain stable in comparison to our main results in Figures 3 to 6.

Second, in Section C.2 we test whether our results are robust to alternative definitions of the control group. We do not observe any substantial changes when (i) adding non-PMM municipalities to our control group (reported in Figures C.5 to C.8) or when (ii) excluding PMM municipalities that employed no PMM doctors in October 2018 from our control group (Figures C.9 to C.12).

Third, our main results were based on equation (1), where treatment is defined as categorical variables indicating different groups of municipalities, more *versus* less exposed to the Cuban exit. We now present results from an alternative specification, based on equation (2), where exposure is defined as the share of Cuban PMM doctors among all local primary care doctors at baseline interacted with time fixed effects. Section C.3 replicates our main results. We observe similar patterns. Overall, pre-trends oscillate around zero up to October 2018, when we observe a sharp decline in service utilization (Figures C.13 and C.14). Again, we do not observe any systematic effects on hospital admissions and mortality (Figures C.15 and C.16). In general, results remain qualitatively similar in comparison to Figures 3 to 6.

Finally, as mentioned in Section 3, not all primary care consultations contain information on specific conditions. As a result, Figure 4 plots coefficients only for a subset of all consultations that occurred during our sample period. To ensure that these results are not driven by outliers with more/less information on specific conditions, we re-estimate effects on consultation categories, each time omitting one municipality from the estimation. For simplicity, we re-code the categorical treatment variable into a dummy variable that takes the value of 1 if a municipality has been affected by the Cuban exit and zero otherwise. Figure C.17 plots the differences between the estimated coefficients from the leave-one-out analysis and the coefficients from the full model for all 4,163 iterations. The closer to the horizontal line at *y* = 0, the less the point estimates from the leave-one-out analysis diverge from the point estimates in the full model. Gradually omitting municipalities from the analysis leaves all estimated coefficients from Figure 4 virtually unchanged. The largest deviation between coefficients from the full model and coefficients from the leave-one-out analysis is smaller than 0.01.

## 6 Impact Pathways

### 6.1 Conceptual Discussion

The Cuban withdrawal from PMM led to a large-scale exit of more than 8,000 primary care doctors, affecting over 2,800 municipalities. The doctor exit led to a reduction in the production of primary care services at aggregate levels, but hospitalizations and mortality remained unaffected. Although we are not able to precisely separate demand and supply factors, we can explore different intermediate outcomes to help explain that overall pattern and also characterize health system adaptation through changes in healthcare inputs and outputs.

Based on the existing literature, we can identify potential channels through which the negative shock in the supply of doctors may affect healthcare utilization and health outcomes. These channels include changes within and beyond the affected providers, which in the Brazilian case refer to public primary care centers. Within affected primary healthcare centers, doctor exit can lead to changes in the workload, tasks and responsibilities assumed by other primary healthcare professionals (Carrillo & Feres, 2019). The exit can also lead to disruptions in the patient-doctor relationship and to changes in patient exposure to doctors’ quality and practice styles once new doctors are hired (Fadlon & Van Parys, 2020; Staiger, 2022). These factors can result in changes in the profile of treatment and service delivery (Reddy et al., 2015).

Beyond the affected primary care centers, both demand and supply-side factors can mediate effects on service utilization and health outcomes. On the one hand, variation in the network or the quality of providers can lead to demand diversion towards alternative health facilities. As a response to service disruption in primary care units, patients may seek care at other public facilities or at fee-for-service providers (Bhalotra et al., 2020; Sabety et al., 2021; Simonsen et al., 2021). Moreover, this mobility may occur within or across localities. On the other hand, health systems may respond to the negative shock in the supply of doctors and to the potential demand diversion by restructuring the supply of facilities and available services.

Guided by these potential pathways, we now discuss different intermediate outcomes to better understand responses to the shock. We start by examining responses at the micro level, looking at adaption within primary care centers and changes in access to different types of treatment and to prescription drugs. We then move on to examine resource reallocation and healthcare fragmentation within municipalities, and finally potential spillover effects to neighboring municipalities or towards private care.

### 6.2 What Changed within Primary Care Services?

#### 6.2.1 Doctors’ Profile, Workload and Practices

We start by investigating the profile of doctors who replaced the Cubans.^18^ We first investigate how affected municipalities managed to attract new primary healthcare doctors and to fill vacancies in PMM. Qualitative evidence suggests that the newly recruited doctors, both PMM and non-PMM, had often little work experience and stayed only for a short time in their new jobs (Poder360, 2019). In contrast, the vast majority of Cuban doctors were experienced general practitioners with at least five years of practice, typically specialized in primary care. Moreover, they were widely perceived as highly accessible, well integrated into their communities, and consistently assigned to the same municipalities for several years (Comes, Trindade, Shimizu, et al., 2016; Rech et al., 2018). These characteristics likely facilitated the accumulation of local knowledge and strong patient–doctor relationships (Sobrinho, 2018). Descriptive patterns corroborate these qualitative accounts. Appendix Figure D.1 documents sharp increases in the share of inexperienced doctors immediately after the Cuban withdrawal. The proportion of PMM novices rose to roughly 15% in treatment group one and 41% in treatment group two. A similar pattern emerges among non-PMM primary care doctors, where the share of inexperienced professionals in treatment group two peaked at 21% in December 2018. These descriptive trends suggest that many of the posts vacated by Cuban doctors were filled by newly entering or inexperienced physicians.

To complement these descriptive patterns and examine the timing more formally, we construct monthly measures of physicians appearing in the CNES data for the first time (those with no prior record of practicing in Brazil) and estimate event-study models analogous to those used for our main analyses. The event-study estimates reveal large and immediate increases in the entry of new doctors into affected municipalities following the Cuban withdrawal, confirming that vacancies were predominantly filled by novices rather than by experienced physicians relocating from elsewhere (see upper plot of Figure D.2). Replacing experienced with inexperienced primary care doctors might partly explain the persistent decrease in health care usage.^19^

Next, we examine the participation and tasks performed by healthcare workers other than doctors in public primary care centers. In the short-term, when vacancies had not been filled yet, non-doctors, such as nurses or community health workers, might have taken over doctoral duties. Carrillo and Feres (2019) show that the introduction of PMM led to doctors taking over tasks from nurses, resulting in null-effects on infant health outcomes. The opposite might have occurred as PMM doctors left the program. Our data allow us to examine the extensive and intensive margins of adjustment of the labor supply of these professionals, and to identify which task was carried out by which healthcare professional. Overall, we do not find evidence for a substitution of doctors by non-doctors. Figure D.3 shows that there were neither meaningful increases in the number of non-doctor primary healthcare workers in public primary healthcare facilities (upper plot), nor in the average weekly hours worked by non-doctors (lower plot). Importantly, in Figure D.4 we do not observe changes in the number of consultations, procedures, and home visits carried out by non-doctors. The results therefore do not indicate a substitution effect or an increased workload for these workers. If anything, the exit of Cuban doctors might have led to a complementary decrease in procedures performed by non-doctors in the short term.

In sum, we do not observe an improvement in the experience profile of the new doctors that replaced the Cuban professionals. We neither observe an increase in the supply of other healthcare professionals, nor task substitution across different healthcare professionals. In that sense, while some of these factors may help explain changes in service utilization, the replacement of professionals of different quality, the reallocation of practices among professionals, and changes in the supply of other health professionals are unlikely explanations for the lack of effects of the doctors’ exit on health outcomes.

#### 6.2.2 Health Service Delivery

We now assess the profile of the services delivered at primary care centers by examining how doctor exit affected different types of consultations and access to medication. First, we can broadly distinguish between two types of consultation: scheduled and spontaneous ones. Scheduled consultations are further subdivided into one-time consultations and consultations which are part of ongoing care. Spontaneous consultations are divided into walk-ins and urgent care. We estimate effects for each type of consultation.

Figure D.5 shows the results. Urgent care remains stable through the first months after exit, even though it is the consultation type with the highest doctoral share (70% in October 2018). However, urgent care requires prioritization by the health team even when severely understaffed and cannot be postponed. For spontaneous walk-ins and scheduled one-time care, we see a strong short-term drop of around 12% and 11% (around 272 and 336 per 100,000) in treatment group one, and 12% and 18% (around 384 and 616 per 100,000) in treatment group two during the first months in the aftermath of the shock, respectively. For consultations focused on health conditions requiring scheduled ongoing care, we observe initial decreases of 8% (around 183 consultations per 100,000) in treatment group 1 and 14% (around 392 per 100,000) in treatment group 2. These effects stabilize at around 5% (around 114 per 100,000) and 10% (around 280 per 100,000) fewer scheduled ongoing consultations throughout 2019. These results suggest a restructuring of service delivery towards a prioritization of urgent and curative care over ongoing care for chronic diseases. This is consistent with evidence indicating that the latter can be delayed without immediate adverse health effects (Shurtz et al., 2022).

If primary care centers accommodate the negative doctor supply shock by de-prioritising ongoing care and care for chronic conditions, how does this affect patients that require related essential services, such as access to prescription drugs? Many essential prescription drugs in Brazil can be purchased at highly subsidized prices (Américo & Rocha, 2020). As access to subsidized drugs in Brazil is conditional on a medical prescription, the decrease in healthcare utilization for chronic diseases might translate into a decrease in related medication utilization. When examining trends in medication for diabetes and hypertension, we see a short but pronounced decrease in medication purchased. However, this is limited to shortly after the exit of the Cuban PMM doctors. In December 2018, the value of medication sold for diabetes and for hypertension (Figure D.6) decreased both by roughly 5% (around R$ 1,523 and R$ 4,471, respectively) in treatment group two. The reduction in medication sales quickly disappears and does not persist in the longer run. Plausibly, patients in need of prescription medication for chronic diseases were therefore able to secure access to medications despite the reductions in primary care consultations. While patients are supposed to renew their prescription every month, doctors can write prescriptions covering up to 180 days (Américo & Rocha, 2020). Patients and/or doctors may have responded to the shock by guaranteeing access to medication even in a setting where access to consultations was restricted. Such adaptation would be in line with a prioritization of services concerning the most immediate needs, thus consistent with a pronounced but transitory drop in drug sales.

Finally, the null effects on hospitalizations and mortality could also reflect a reduction in unnecessary or low-margin consultations arising from moral hazard in public healthcare systems (Einav & Finkelstein, 2018). Before the Cuban exit, patients may have overused primary care services for chronic conditions, potentially generating consultations with limited clinical value. After the exit, patients or providers may have curtailed these low-benefit visits without adverse consequences for health.

Such moral hazard, however, is unlikely to explain our results. Primary care doctors in SUS have no financial incentives to provide unnecessary services. They follow standardized clinical guidelines and receive fixed salaries, independent of service volume. Moreover, Brazil faces a large and growing burden of chronic conditions, and evidence points to substantial unmet demand for routine monitoring and follow-up (Schmidt et al., 2011). Recent national healthcare performance indicators reinforce this interpretation. For 2022–2024, SISAB published indicators measuring whether patients with hypertension or diabetes received at least one consultation and the recommended basic follow-up tests in the past semester. Benchmark compliance is set at 50%, yet actual values are markedly lower. For hypertension, compliance averages 35% in control PMM municipalities, with 31% in treatment group one, and 34% in treatment group two. For diabetes, the figures are 30%, 27%, and 29%, respectively. If low-value or excessive consultations were widespread, we would expect substantially higher scores.

While we cannot rule out the possibility that a small subset of patients engaged in low-value care, the institutional features of Brazil’s primary care system and the descriptive evidence above suggest that moral hazard is unlikely to be the mechanism driving the observed reductions in chronic-care consultations. We therefore view this explanation as a less plausible driver of the null effects on hospitalizations and mortality.

In sum, the results suggest the possibility of demand adaptation and of restructuring of health service delivery towards urgent and curative needs as well as other protective services, buffering patients against more extreme adverse outcomes such as hospitalizations and deaths.

### 6.3 What Changed Beyond Primary Care Services?

We now examine whether the Cuban exit had impacts beyond the changes in the profile of professionals and services delivered at primary care centers. We first assess impacts on service use from other healthcare providers. We then examine private insurance markets and spillover effects on neighboring municipalities.

#### 6.3.1 Other Healthcare Services and Providers

With a reduction in the availability of primary care consultations, patients might seek care in other sources of health services. The existing studies on small-scale events from developed contexts provide evidence that doctor exit or turnover can lead to patients substituting primary care by directly seeking special, hospital, or emergency care (Agha et al., 2019; Sabety et al., 2021). Patients could also opt to increasingly use private healthcare by resorting to out-of-pocket spending and the purchase of private insurance plans (Silva et al., 2022). To investigate whether patients bypass the primary care sector to seek ambulatory care at other facilities, we turn to data on ambulatory care visits beyond primary care centers, distinguishing between specialist, hospital, and emergency room (ER) visits, as well as by complexity of treatment (basic, medium, high). Primary care needs mostly fall under the basic complexity category.

While we do not find significant changes for the use of specialists or hospitals for basic procedures (see Figure D.7), we do find a suggestive increase in the general use of emergency care facilities, particularly in municipalities where all PMM doctors were Cuban. The upper plot of Figure D.8 shows that gradually more ER facilities in treatment group two start delivering basic ambulatory care services, reaching significant increases of around 3.5%-4% from July 2019 onward. In the lower plot, although point estimates are less precise, we also observe that, from January 2019 onward, emergency care of basic complexity increased by more than 20% in treatment group 2, only occasionally dipping back to pre-treatment levels. Patients therefore seem to partly substitute treatment at primary care centers with treatment at facilities providing emergency care, while municipalities start providing more low-complexity services in these facilities. It is important to note that the diversion persists over time. Although ERs are very accessible to the general population and cannot deny services to patients, they are explicitly not set up to take care of ongoing care or deferrable treatments (Carret et al., 2009). These upward trends in the use of ER facilities thus suggest increased fragmentation and misallocation of resources within municipalities, with preventive care being reduced and basic services being diverted towards facilities that should handle more urgent cases and emergencies.

#### 6.3.2 Private Insurance and Private Services

Patients might substitute publicly provided care with private healthcare services when primary care becomes less available. In the context of the PMM, however, the scope for such substitution is structurally limited. The program primarily served low-income and underserved municipalities, especially in areas where reliance on Brazil’s public health system (SUS) is close to universal. Administrative data from CNES and national survey data show that private service availability and private insurance coverage in these areas are substantially below the national average.^20^

Consistent with this, we do not observe any systematic changes in private insurance enrollment following the Cuban withdrawal (see Figure D.9). Although we cannot directly measure out-of-pocket private expenditures, the socioeconomic profile of the affected municipalities reinforces the expectation that substitution toward privately paid services is minimal. Because SUS provides free care at the point of use, low-SES patients have long relied on public services when seeking medical attention. In this sense, we expect that patients facing reduced access to PMM primary care would mainly bypass local clinics in favor of other public facilities, rather than switch into private care. Offsetting effects through private healthcare utilization are therefore unlikely.^21^

#### 6.3.3 Geographical Spillovers

Finally, patients from affected municipalities might seek care in neighboring municipalities that have been unaffected or less affected by the exit. This type of spillover is nevertheless unlikely in our setting. Primary healthcare units are explicitly set up to cover a specific catchment area and its resident population. Still, spillovers might be active to some extent.

We test for spillover effects between neighboring municipalities by accounting for neighbors’ average treatment status. First, for each municipality *i*, we calculate the average share of Cuban PMM doctors among all primary care doctors of *i*’s neighboring municipalities in October 2018. We then follow equation 2, where we include both the interactions of municipality *i*’s baseline share of Cuban doctors with time dummies, and the interaction of neighbors’ average baseline share of Cuban doctors with time dummies. If patients from affected municipalities seek primary care in neighboring municipalities, we would expect a positive relationship between the average treatment intensity of municipality *i*’s neighbors and municipality *i*’s primary care consultations or procedures after the Cuban exit. The upper plot in Figure D.10 reports the coefficients from the interaction between neighbors’ average share of Cuban doctors and the time dummies, for consultations per 100,000 residents as the outcome variable. We do not find any evidence of spillover effects.

Second, for all municipalities that were not directly affected by the Cuban exit (both non-PMM and PMM municipalities without Cuban doctors), we regress primary care consultations on an indicator for whether the municipality has at least one treated neighbor. Again, if diversion occurs, primary care activity should rise in municipalities bordering treated ones after the shock. Results reported in the lower plot of Figure D.10 do not support the presence of meaningful spillovers. Point estimates are very close to zero, and confidence intervals are narrow enough to rule out modest increases. These results reinforce the view that the missing consultations in affected municipalities were not absorbed by neighboring primary care units.

### 6.4 Costs and Fiscal Implications

Given data constraints, our analysis can offer only a coarse assessment of how the Cuban withdrawal affected healthcare costs. Two categories of expenditures are partially observable in administrative sources, personnel costs and variable treatment costs.

PMM doctors received a fixed federal stipend of R$ 10,000 per month. In contrast, non-PMM primary care physicians earned on average R$ 13,200 per month in 2018, although wages vary considerably across municipalities and are not systematically recorded (G1, 2018b). Prior to the withdrawal, treatment group one had an average of four Cuban PMM doctors per municipality, while treatment group two had two. By April 2019, most vacancies had been filled by new PMM doctors and additional non-PMM hires. The data indicate that municipalities in both treatment groups hired roughly one additional non-PMM doctor, on average, following the Cuban exit. Assuming one-for-one replacement, substituting a PMM doctor with a non-PMM physician raises personnel costs by at least R$ 3,200 per doctor per month, which may be considered a lower bound given market pressures to restore primary care services quickly. Although overall doctor numbers changed little in the medium term, personnel spending therefore increased. Treatment group one municipalities spent approximately R$ 700 more per month despite having slightly fewer doctors, while treatment group two spent roughly R$ 3,200 more per month while maintaining doctor availability constant.

We use SIA data to examine federally reimbursed ambulatory care expenditures. These represent only variable procedure costs and exclude fixed or municipal co-funding, therefore providing a lower-bound indicator of cost shifts. Event-study estimates show that in treatment group two, ambulatory care spending in facilities offering emergency services increased noticeably during the post-withdrawal period, by roughly 9.1% in March 2019 and 13.2% in December 2019 (see Figure E.1). A simple pre/post comparison indicates an average increase of 5.5% in these facilities. Considering only federal reimbursements, this corresponds to approximately R$ 1,700 per municipality per month.

Taken together, personnel and treatment costs increased by approximately R$ 700 per month in treatment group one and R$ 4,900 per month in treatment group two. Aggregating across affected municipalities yields an estimated lower-bound increase of roughly R$ 8.6 million per month (about US$ 2.2 million) in nationwide expenditures attributable to the Cuban withdrawal. However, these values should be interpreted strictly as lower-bound estimates, given the absence of data on fixed costs and municipal co-financing. Nonetheless, they provide a first-order indication that the Cuban exit increased the fiscal burden on the Brazilian healthcare system. In addition to these fiscal costs, the replacement of Cuban physicians with less experienced doctors may also entail foregone benefits. As documented in Section 5, the shift toward novice professionals coincided with a deterioration in the management of chronic conditions and a persistent reduction in routine primary care consultations. While these consequences are harder to quantify in monetary terms, they suggest that the Cuban withdrawal may have generated not only higher expenditures, but also a decline in the effectiveness and continuity of primary care delivery.

## 7 Final Comments

In this paper, we investigated the effects of a large-scale exit of doctors in Brazil, where over 8,000 doctors from more than 2,800 municipalities left the country. We found a meaningful decrease in healthcare utilization, but no effect on hospitalizations and mortality during our sample period. These results are robust to multiple checks and sensitivity analyses.

The results suggest that patients and local healthcare systems were able to adapt within a few months, and generally proved flexible when under distress. Adaptation nevertheless came at the cost of de-prioritizing ongoing care and care for chronic conditions. This may be an issue over the longer-run as chronic conditions require prevention, early detection and ongoing care. Existing challenges, such as under-diagnosis and insufficient treatment of chronic conditions, may be exacerbated by doctor exit as it implies a loss of location- and position-specific knowledge (Manne-Goehler et al., 2019; Mauer et al., 2022). We also found increasing ER use for low-complexity treatments, especially where all PMM doctors were Cuban. This pattern is typically not efficient, does not facilitate long-term patient-doctor relationships, and undermines patient education (Carret et al., 2009; Gill, 1999).

Many countries struggle to train, recruit, and appropriately distribute health professionals across their regions. Whilst the impacts of a large-scale exit of doctors in Brazil suggest local healthcare services in vulnerable areas are resilient and can adapt to protect population health outcomes in the short-term, health services in these areas remain under-resourced and the health of local populations lags behind other areas of the country. Addressing these challenges may require prioritizing policies that target the structural factors negatively affecting healthcare access and quality in under-served regions.

## Supplementary Material

Supplementary Materials

## Footnotes

1 Such as physician retirement or death (Fadlon & Van Parys, 2020; Sabety, 2023; Sabety et al., 2021; Schwab, 2023; Zhang, 2022), practice closures (Bischof & Kaiser, 2021; Simonsen et al., 2021), and changes in managed care networks (Staiger, 2022).

2 Media articles and institutional reactions provide support to the view that the Cuban withdrawal came as a surprise and needed an urgent response. For instance, the Brazilian Senate lamented the “abrupt” Cuban exit (Agencia Senado, 2018), the National Confederation of Municipalities, a political association representing municipalities’ needs on the federal level, stated their “extreme” worry about the Cuban exit (G1, 2018a), while Gilberto Occhi, then Brazil’s Minister of Health, said that the Cuban exit led to a situation of “urgency” (Folha de S.Paulo, 2018).

3 Robustness checks show that results remain stable upon including these municipalities into our control group.

4 We exclude the 34 special indigenous health districts from our analysis, as they can not be adequately captured by municipal borders.

5 We were able to match around 81% of PMM doctors to their corresponding CNES information.

6 Drugs for diabetes and hypertension are fully subsidized and free for patients, while prices for other medications did not change during our sample period.

7 Brazil’s administrative health information systems are widely regarded as high-quality and stable over time. Vital statistics, in particular, have consistently been classified as ‘high quality’ in recent assessments preceding our study period, with evidence of continued improvements over time (França et al., 2020). Importantly, because the reporting of hospital admissions and deaths is mostly carried out by administrative bodies that were not affected by the withdrawal of primary-care physicians, it is unlikely that our estimates are affected by any deterioration in data quality.

8 Bolsa Familia is a conditional cash transfer program in Brazil that targets poor households. In 2018, the program covered over 43 million individuals (SENARC, 2025).

9 Doctors working in multiple healthcare facilities in the same municipality are not double-counted.

10 Disaggregating consultations by conditions, however, comes at the cost of decreasing data quality due to missing values. On average, around 40% of all primary care consultations have no information about the related condition and the responsible professional (Table A.1).

11 For each event-study specification, we report in the figure notes the p-value from a joint Wald test of the null that all pre-treatment coefficients equal zero. Because these tests span a relatively large number of coefficients, they can reject even when the underlying coefficients are small, lack direction, or reflect isolated fluctuations rather than systematic pre-existing trends. In several cases, the coefficients in the months immediately preceding the Cuban exit, the periods most relevant for assessing parallel trends, show no meaningful movement. For this reason, we interpret the parallel-trends assumption primarily through the visual inspection of event-study estimates, with the joint tests serving as a complementary diagnostic.

12 Cuban doctors left Brazil from mid-November 2018 onward. Thus, December 2018 was the first month in which vacancies were reported. One reason why the estimated decline in treatment group two is slightly short of 100% is that the Brazilian government might have managed to fill few of the resulting vacancies in the second half of November 2018.

13 Throughout the analysis, we use group-specific means from Table A.4 to interpret effect sizes in absolute numbers.

14 Causes amenable to primary care are defined by the Brazilian Ministry of Health. These include, for example, conditions that are preventable through immunization, prenatal and birth-related conditions, and different chronic conditions such as asthma, hypertension, and diabetes.

15 Similarly, Figure B.4 presents event-study estimates of total hospitalizations and total mortality among individuals aged 60 and above, and shows no systematic movements following the Cuban exit, consistent with the null effects documented for other health outcomes.

16 For rarer hospitalization causes, such as hypertension or diabetes among individuals aged 60 or older, the estimates are naturally less precise. Here, the confidence intervals allow for larger potential effects (27%-44%). However, it is important to note that these are outcomes with low baseline incidence and limited variation over time, which inherently produce wider confidence intervals. Despite this lower statistical precision, point estimates do not display any consistent directional pattern that would suggest underlying adverse effects.

17 We also estimate all health-outcome specifications using quarterly aggregates with the goal of reducing noise. The resulting patterns are qualitatively similar to the monthly analyses and do not reveal consistent evidence of changes in hospitalizations or mortality. Because monthly data capture more detailed adjustment dynamics in the weeks following the Cuban withdrawal, we retain them as our main specification. Quarterly results are available upon request.

18 Doctors’ profiles and related practice styles can impact healthcare utilization and health outcomes for multiple reasons. First, doctors’ individual and shared work experience is important for the quality of services that they deliver (Y. Chen, 2021). Second, healthcare professionals’ performance and practice styles have a strong influence on their patients’ healthcare utilization (Fadlon & Van Parys, 2020; Simonsen et al., 2021). And third, it implies a loss of team and unit-specific human capital built up over various years (Bartel et al., 2014).

19 To assess whether part of the replacement reflected reallocation from outside the PMM program, we also track physicians previously employed in non-eligible municipalities. Event-study estimates indicate some relocation to affected PMM municipalities immediately after the Cuban withdrawal (roughly 0.9 additional doctors per 100,000 residents in treatment group one and 2.7 in treatment group two in December 2018), but these flows are considerably smaller than the increases observed among newly entering doctors (see lower plot of Figure D.2). Together, the patterns suggest that migration from non-eligible municipalities played a secondary role relative to new doctor entry, consistent with limited spillover or broader general-equilibrium effects.

20 Private insurance coverage is estimated at 7% in rural areas *versus* 32% in urban areas (Souza Júnior et al., 2021).

21 To further assess this possibility, we examine heterogeneity in health outcomes by the baseline presence of private hospitals, using CNES records as a proxy for local private-sector capacity. We observe no systematic differences between municipalities with and without private providers at baseline (results upon request).
