# Supplementary material for: What Happens to Population Health When the Doctors Leave? Evidence from the Exit of Cuban Doctors in Brazil
Source: J Health Econ. Author manuscript; Available in PMC 2026 Oct 16. (PMC7619565; doi:10.1016/j.jhealeco.2025.103099)
Supplement: Supplementary file 1 [file EMS220008-supplement-Supplementary_Material.pdf]

Online Appendix

What Happens to Population Health When the Doctors Leave?
Evidence from the Exit of Cuban Doctors in Brazil

By Stefan Sliwa Ruiz, Malte Becker, Thomas Hone, and Rudi Rocha

November 2025

A Additional Descriptive Statistics

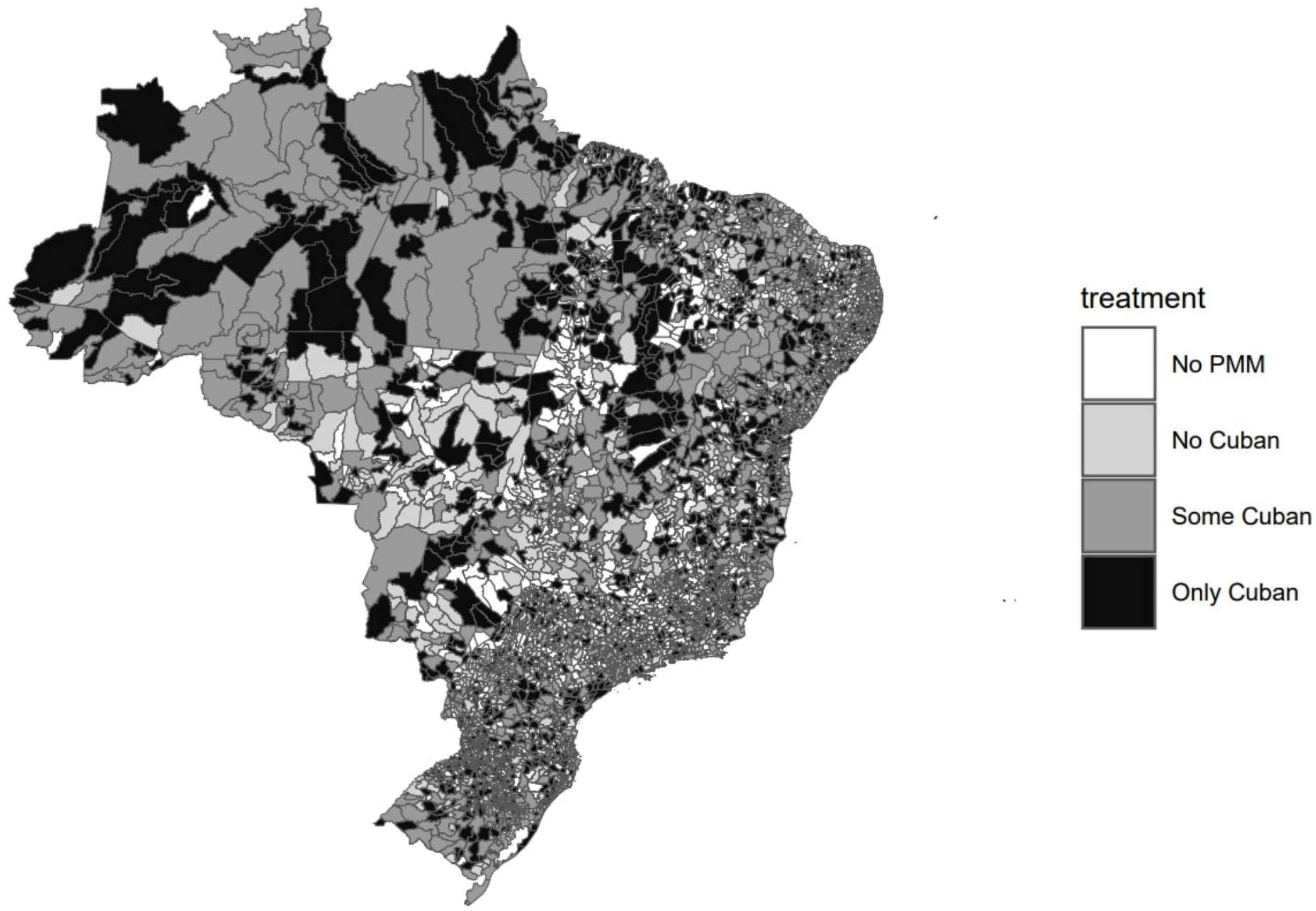

Figure A.1: PMM allocation of Brazilian municipalities in October 2018 (last month before the Cuban exit). White municipalities did not participate in PMM and are excluded from the analysis. Light grey municipalities had no Cuban PMM doctor in October 2018, grey municipalities had some Cuban PMM doctors and black municipalities had only Cuban PMM doctors. Original data from the Brazilian Ministry of Health.

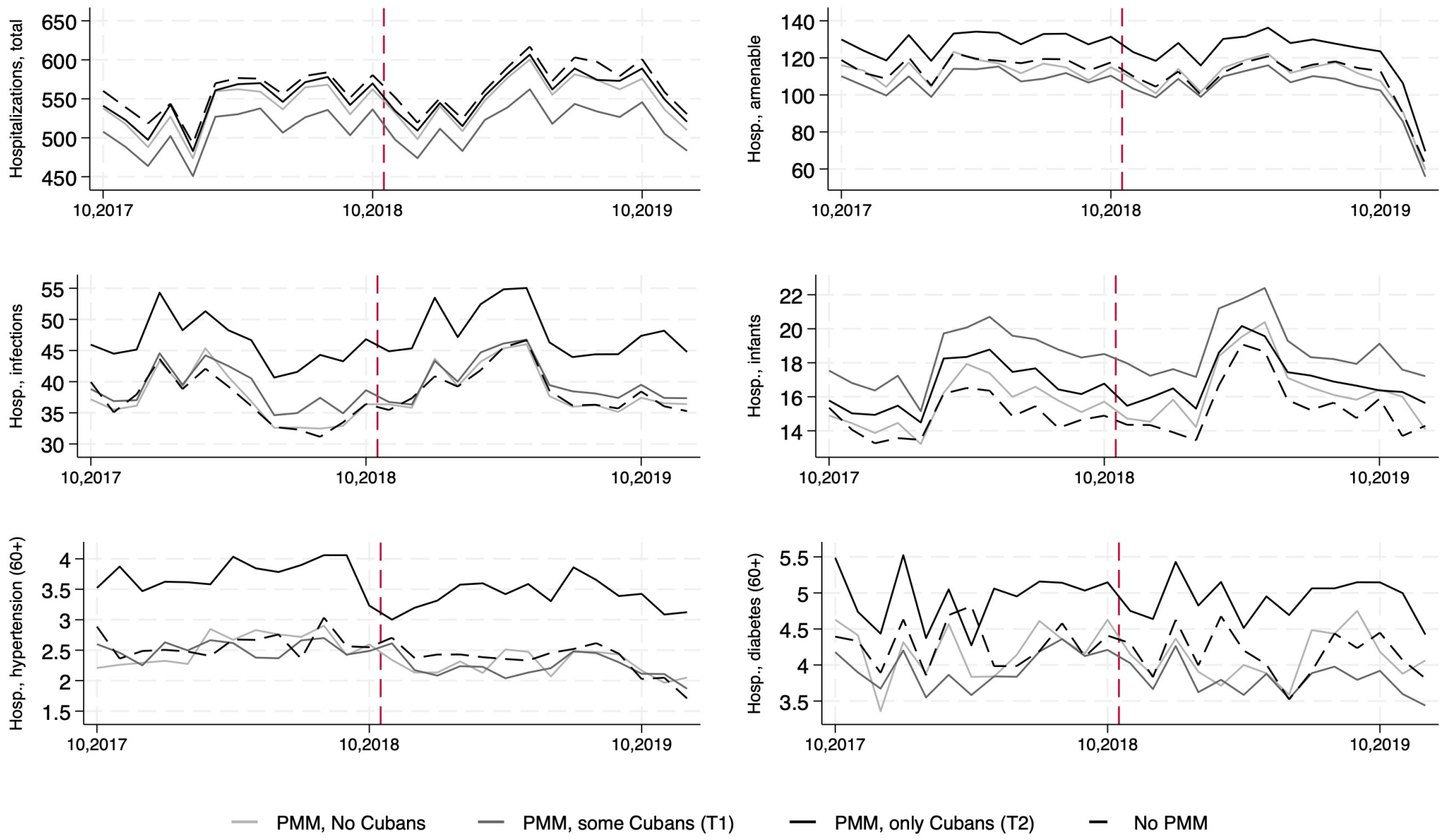

Figure A.2: The graph shows trends in the group means of the number of hospitalizations in six different categories, per 100,000 residents. Where indicated, the number of hospitalizations only includes individuals aged 60 and above. The series in dark grey presents treatment group one, the series in black presents treatment group two. The dashed vertical lines present the pre-treatment month October 2018.

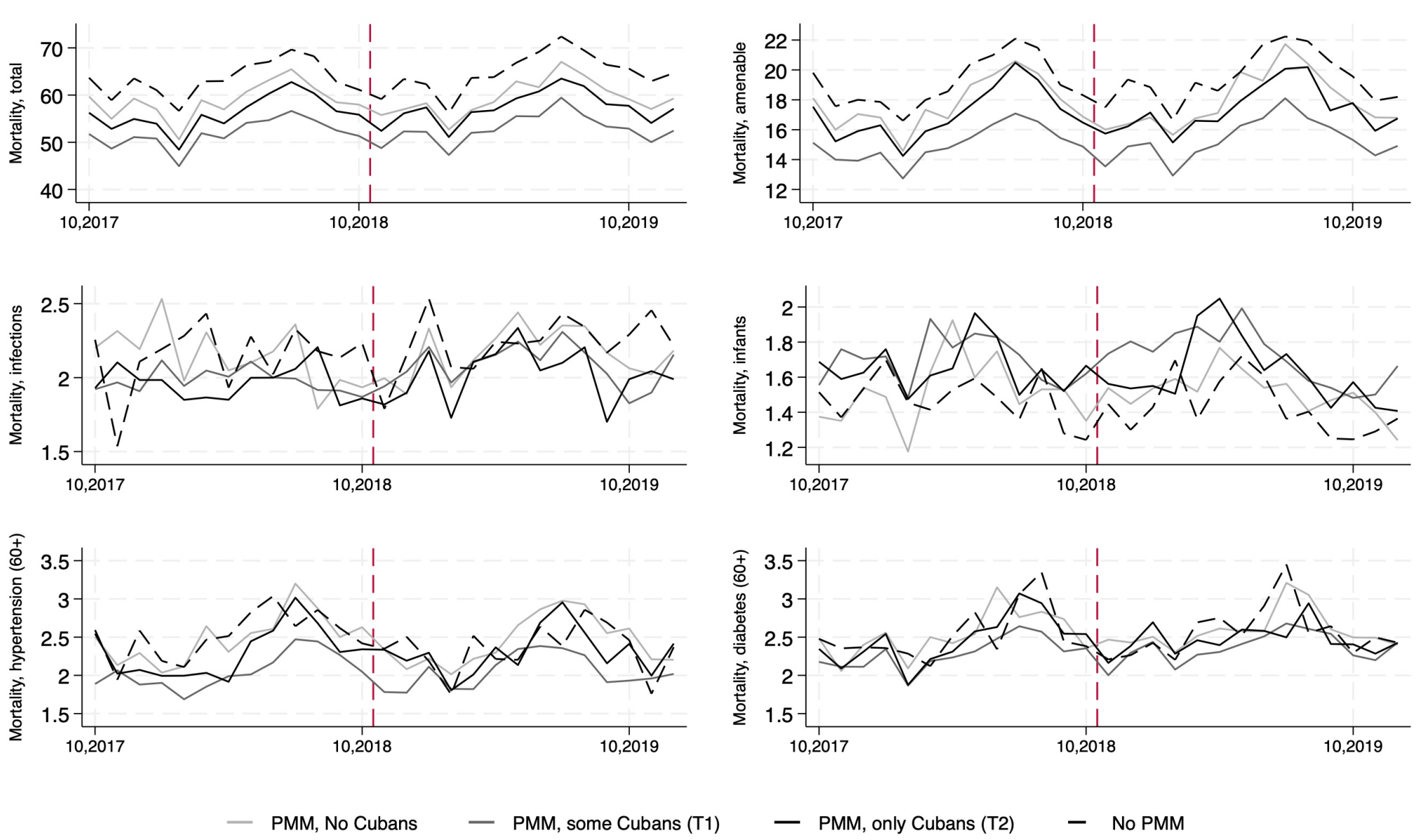

Figure A.3: The graph shows trends in the group means of the number of hospital mortality in six different categories, per 100,000 residents. Where indicated, the number of hospital mortality only includes individuals aged 60 and above. The series in dark grey presents treatment group one, the series in black presents treatment group two. The dashed vertical lines present the pre-treatment month October 2018.

Table A.1: Primary Care Consultations: Conditions and Consultation Types by Responsible Professionals, PMM Eligible Municipalities

	Mean per 100k inhab.	Share doctors	Share non-doctors	Share no information
**Consultations**	14,527	.64	.36	0
	By condition			
**Related to chron. cond.**	4,196	.61	.34	.06
Asthma	68	.87	.13	0
COPD	43	.8	.2	0
Diabetes	601	.7	.29	.01
Hypertension	1,738	.69	.25	.06
Mental health	1,149	.66	.32	.02
Breast cancer screening	205	.45	.54	.01
Cervical cancer screening	333	.32	.67	.01
Cardiovascular screening	61	.68	.32	0
**Related to risk factors for chron. cond.**	1,547	.48	.5	.02
Malnutrition	74	.74	.26	0
Obesity	224	.57	.43	0
Smoking	65	.68	.32	0
Alcohol	32	.73	.27	0
Other drugs	16	.64	.36	0
Rehabilitation	1,136	.31	.67	.02
**Related to maternal health**	2,817	.45	.51	.04
Prenatal care	638	.51	.48	.01
Pediatrics	1,215	.45	.52	.03
Postpartum care (first 42 days)	48	.37	.63	0
Sexual and reproductive health	915	.39	.58	.03
**Related to infectious diseases**	60	.57	.43	0
STDs	34	.61	.39	0
Dengue	5	.77	.23	0
Hansen's disease	12	.53	.47	0
Tuberculosis	10	.48	.52	0
**Without classified conditions**	5,969			
	By type of consultation			
Scheduled singular care	4,962	.65	.35	0

Continued on next page

Table A.1 – Continued from previous page

	Mean per 100k inhab.	Share doctors	Share non-doctors	Share no information
Scheduled ongoing care	2,606	.41	.59	0
Non-scheduled walk-ins	6,483	.67	.33	0
Non-scheduled initial hearing	1,053	.15	.85	0
Non-scheduled urgent care	259	.7	.3	0
Without classified type	0			

Notes: Average primary care outcomes in rates per 100,000 inhabitants in PMM-eligible municipalities for October 2018 (the pre-treatment month). Numbers within columns do not always sum up as data becomes less precise with disaggregation. Original data from SISAB.

Table A.2: Definitions of the Type of Consultations according to SISAB

Type of consultation	Definition according to SISAB
Scheduled continuous consultations	These are consultations that constitute individual programmatic actions, directed to life cycles, diseases and priority diseases, which require continuous monitoring. As an example, the care given to pregnant women, children, the elderly, people with chronic diseases (hypertension, diabetes) etc.
Scheduled consultations	It comes from spontaneous demand or by direct appointment at the reception, of a non-urgent nature, not attended on the same day of the demand, but scheduled for another day. For example, cases of skin lesions, without signs of inflammation or infection; nonspecific complaints of fatigue; tiredness; chronic headache; change or beginning of contraceptive contraceptive medication, etc.
Initial reception/orientation	Refers to reception done by a senior professional at the moment the user arrives at the health service, reporting complaints or signs of symptoms. When the patient arrives at the health service, reporting complaints or signs and perceived symptoms It does not include flow orientations within the UBS.
Consultation on the same day	A consultation that takes place on the same day that the user seeks service, of a non-urgent nature. As an example, cases with symptoms of great pain or those that cannot wait for an appointment on another day, such as low back pain, sore throat, urinary symptoms, etc. Another characteristic for same-day care may be related to the social or psychological vulnerability of the patient.
Urgent consultations	Care provided to the patient when there is a possibility of worsening of the condition or risk of life and that determines the need for immediate relief of physical and/or psychological suffering, recovery of the health condition, stabilization/life support and/or referral to another part of the network when necessary. For example, cases of chest pain, neurological symptoms and/or symptoms and/or neurological signs, hypertensive emergency, etc.

Table A.3: Treatment Groups by Region

	Center-West	North	North-East	South	South-East
PMM, no Cubans (C)	162 (45.1%)	41 (10.9%)	403 (28.1%)	308 (33.3%)	406 (38.1%)
PMM, some Cubans (T1)	88 (24.5%)	162 (43.0%)	491 (34.2%)	217 (23.4%)	302 (28.3%)
PMM, only Cubans (T2)	109 (30.4%)	174 (46.1%)	541 (37.7%)	401 (43.3%)	358 (33.6%)

Notes: The number and share of municipalities by treatment group and macro-region. Original data from the Brazilian Institute of Geography and Statistics (IBGE).

Table A.4: Municipality Averages of Main Variables in October 2018

	Pooled		PMM, by Treatment Groups			T-test	
	All	PMM	No Cubans (C)	Some Cubans (T1)	Only Cubans (T2)	T1-C	T2-C
**Primary health care professionals**							
Primary care doctors (per 100k)	41.89	40.05	42.17	33.92	43.15	-8.25***	0.98
Non doctors (per 100k)	612.80	605.98	606.50	596.06	613.45	-10.44	6.95
PMM doctors (per 100k)	12.43	16.61	11.24	18.44	19.62	7.20***	8.38***
Cuban PMM doctors (per 100k)	7.84	10.47	0.00	9.95	19.62	9.95***	19.62***
Share of Cubans among PMM doctors	0.43	0.59	0.00	0.52	1.00	0.52***	1.00***
Share of Cubans among all PC doctors	0.14	0.18	0.00	0.21	0.31	0.21***	0.31***
Primary care doctors, non-PMM (per 100k)	31.77	25.30	33.25	16.48	25.71	-16.77***	-7.53***
Av. weekly hrs., primary care doctors	35.67	36.11	35.66	36.02	36.57	0.36	0.91***
Avg. weekly hrs., non-doctors	37.57	37.59	37.65	37.24	37.82	-0.41***	0.16**
Total hours, non-doctors (per 100k)	22,946.97	22,704.28	22,774.73	22,110.93	23,118.35	-663.80**	343.62
PC doctors, new entries (per 100k)	0.84	0.70	1.04	0.47	0.61	-0.57***	-0.44***
PC doctors from unaffected mun. (per 100k)	2.53	3.39	4.38	1.64	3.95	-2.74***	-0.43
**Primary care services**							
Consultations (per 100k)	15,734.29	14,526.52	15,259.93	11,675.53	16,189.99	-3,584.40***	930.06**
Chronic conditions	4,331.27	4,196.36	4,241.59	3,835.15	4,448.37	-406.43***	206.78
Related risk factors	1,703.41	1,547.08	1,746.73	1,112.12	1,731.06	-634.60***	-15.66
Maternal health	2,864.08	2,816.75	2,765.37	2,740.38	2,920.19	-25.00	154.82*
Infections	58.04	59.83	50.44	67.01	61.82	16.57***	11.38
Consultation type, scheduled	3,352.06	3,292.17	3,376.53	3,054.52	3,419.66	-322.00***	43.13
Consultation type, scheduled cont.	2,784.02	2,633.15	2,798.41	2,285.66	2,798.41	-512.76***	-0.00
Consultation type, walk-in	2,995.22	2,804.20	2,854.80	2,265.08	3,204.06	-589.72***	349.26***
Consultation type, urgent	128.37	106.36	98.85	80.47	142.87	-18.38	44.02**
Share consultations by doctors	0.63	0.64	0.63	0.65	0.64	0.02**	0.01
Procedures (per 100k)	16,230.96	14,306.57	15,052.05	10,258.36	16,915.20	-4,793.70***	1,863.15***
Share procedures by doctors	0.03	0.03	0.03	0.03	0.04	-0.00	0.01***
Home visits (per 100k)	26,294.86	25,235.27	26,500.27	21,131.60	27,455.19	-5,368.67***	954.92
Share home visits by doctors	0	0	0	0	0	0	0
Hypertension care score (2022-24)	34.30	33.59	35.35	30.73	34.29	-4.62***	-1.05***
Diabetes care score (2022-24)	29.30	28.71	30.31	26.52	29.10	-3.79***	-1.21***

Continued on next page

Table A.4 – continued from previous page

	Pooled		PMM, by Treatment Groups			T-test	
	All	PMM	No Cubans (C)	Some Cubans (T1)	Only Cubans (T2)	T1-C	T2-C
**Hospitalizations**							
Total (per 100k)	563.10	557.27	562.23	536.46	569.70	-25.78***	7.47
Amenable (per 100k)	119.25	119.83	114.94	110.38	131.42	-4.56	16.48***
Non-amenable (per 100k)	443.86	437.44	447.29	426.08	438.28	-21.21***	-9.02
Chronic conditions (per 100k)	185.57	181.07	188.05	162.99	189.64	-25.06***	1.58
Infections (per 100k)	39.87	41.03	36.39	38.63	46.81	2.24	10.43***
Infants (per 100k)	16.44	16.96	15.71	18.51	16.77	2.80***	1.06
Total 60+ (per 100k)	164.04	156.28	164.87	135.62	165.58	-29.25***	0.71
Diabetes 60+ (per 100k)	4.63	4.70	4.63	4.21	5.15	-0.42	0.52
Hypertension 60+ (per 100k)	2.74	2.81	2.60	2.49	3.23	-0.11	0.64*
**Mortality**							
Total (per 100k)	56.59	55.12	57.99	51.35	55.86	-6.65***	-2.14*
Amenable (per 100k)	16.64	16.10	16.88	14.88	16.48	-2.00***	-0.40
Non-amenable (per 100k)	39.94	39.01	41.12	36.47	39.38	-4.65***	-1.74*
Chronic conditions (per 100k)	36.77	36.25	37.97	33.90	36.67	-4.07***	-1.30
Infections (per 100k)	1.97	1.89	1.93	1.87	1.86	-0.07	-0.08
Infants (per 100k)	1.48	1.55	1.35	1.62	1.67	0.27**	0.31*
Total 60+ (per 100k)	37.87	36.72	38.55	34.17	37.23	-4.38***	-1.32
Diabetes 60+ (per 100k)	2.41	2.42	2.35	2.35	2.54	0.00	0.19
Hypertension 60+ (per 100k)	2.36	2.34	2.63	2.05	2.34	-0.58***	-0.29
**Medication** (in 1,000 R$)							
Subs. hypertension med. (per 100k)	65.29	62.39	69.79	47.37	69.42	-22.42***	-0.337
Subs. diabetes med. (per 100k)	33.28	31.41	34.98	28.82	30.46	-6.16***	-4.52***
**Private healthcare**							
Privately insured (per 100k)	8,355.51	8,208.20	8,238.38	10,416.64	6,424.68	2,178.26***	-1,813.70***
**Ambulatory care outside of UBS**							
Basic complexity facilities, ER (per 100k)	9.73	8.69	10.62	4.95	10.88	-5.67***	0.25
Ambul. care costs, ER (1k R$, per 100k)	251.67	258.82	255.26	318.15	197.40	62.89***	-57.86***
Basic complexity ER procedures (per 100k)	606.65	430.28	662.23	263.07	514.75	-399.16***	-147.48

Continued on next page

Table A.4 – continued from previous page

	Pooled		PMM, by Treatment Groups			T-test	
	All	PMM	No Cubans (C)	Some Cubans (T1)	Only Cubans (T2)	T1-C	T2-C
Basic complexity specialist procedures (100k)	146.39	148.19	159.74	141.43	144.75	-18.31	-14.99
Basic complexity hospital procedures (100k)	161.80	151.76	174.40	113.60	179.35	-60.80**	4.95

Notes: Municipality averages in rates per 100,000 inhabitants for October 2018 (the pre-treatment month). Data on private insurance coverage for September 2018 (the last available pre-treatment period). Data on hypertension and diabetes care scores for the years 2022 to 2024. Where specified, variables only include observations for individuals aged 60 years and above. Column 1 includes all Brazilian municipalities (excluding special indigenous districts). Column 2 includes all municipalities that participated in PMM. Columns 3 to 5 distinguish PMM participating municipalities based on the presence of Cuban PMM doctors in October 2018. Control municipalities had no Cuban PMM doctor in October 2018. Treatment Group One (T1) had some Cuban and some non-Cuban PMM doctors in October 2018. Treatment Group Two (T2) had only Cuban PMM doctors in October 2018. Numbers within columns do not always sum up as data becomes less precise with disaggregation. Data on doctor supply originally from the Ministry of Health. Data on primary care services originally from SISAB. Data on mortality and hospitalizations originally from SIM/Datasus and SIH/Datasus. Data on medication originally from the Ministry of Health. Data on private insurance coverage originally from ANS. Data on ambulatory care in secondary and tertiary care centers originally from SIA and CNES.

Table A.5: Socio-demographic Control Variables on the Municipality Level

				Pairwise t-test	
	Control	Treat 1	Treat 2	T1-C	T2-C
N. families receiving Bolsa Família (per 100k)	9,096	9,731	10,322	635***	1,226***
Bolsa Família expenses per capita (R$)	247	275	302	28***	55***
GDP per capita (R$)	23,565	22,988	21,469	-577	-2,096***
Share of public sector value added	0.33	0.32	0.36	-0.01***	0.03***
Share of population over 65 (%)	0.11	0.09	0.10	-0.02***	-0.00***
Sex ratio in the municipality	1.03	1.00	1.03	-0.03***	0.00
Number of observations	3,963	3,780	4,749		

Notes: Municipality averages in rates per 100,000 inhabitants by treatment group, pooled over the years 2017 to 2019. Original data from the Brazilian Institute of Geography and Statistics (IBGE).

B Additional Results

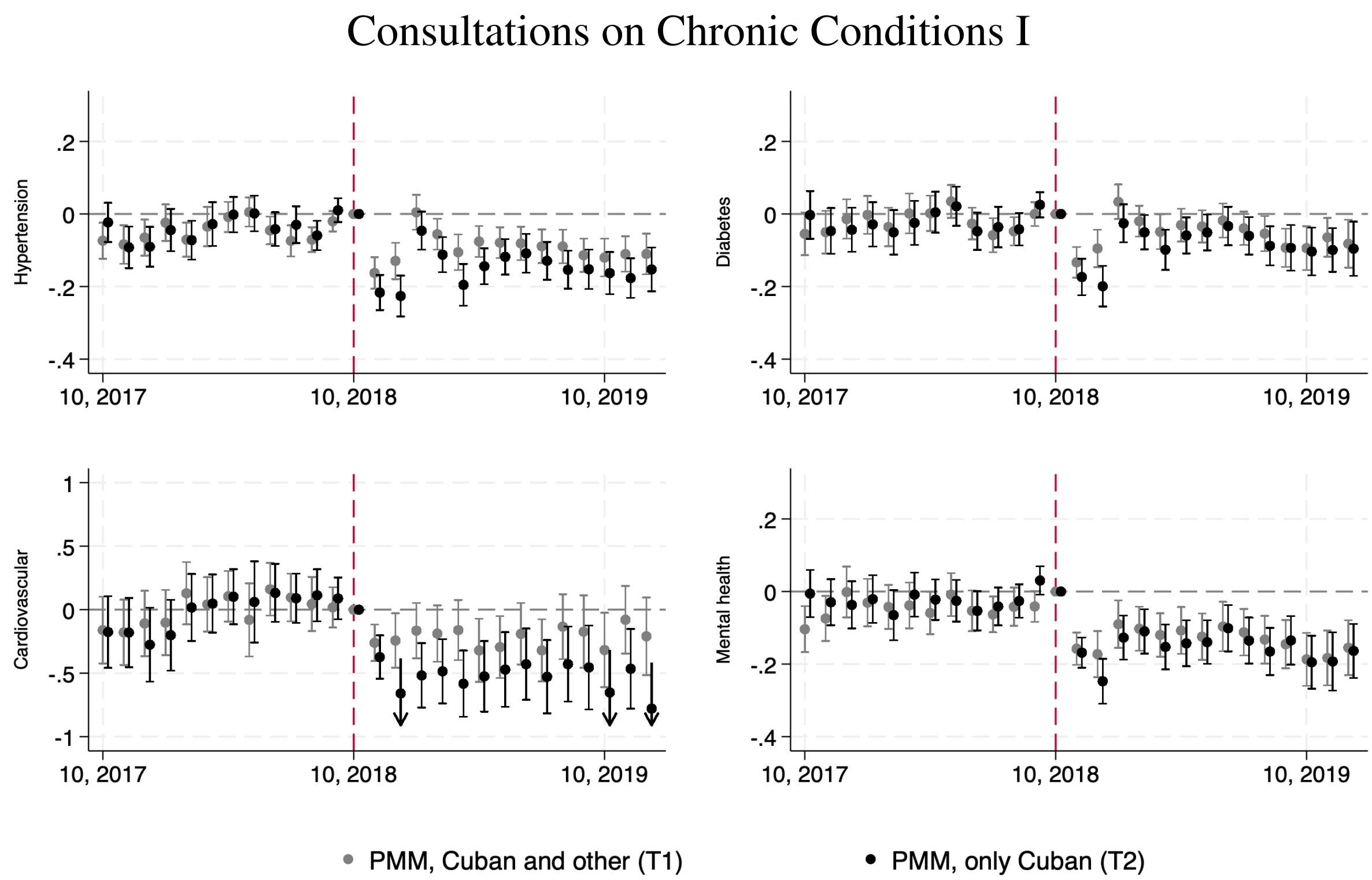

Figure B.1: The graph shows event studies for the number of primary care consultations on hypertension, diabetes, cardiovascular diseases, and mental health, following equation 1. Regressions include municipality and state-by-time (month) fixed effects. Dependent variable measured per 100,000 inhabitants. The series in grey presents treatment group one, the series in black presents treatment group two. Whiskers around the point estimates present 95 percent confidence intervals, where standard errors are clustered at the municipality level. The dashed vertical lines present the pre-treatment month October 2018. Joint p-value of pre-treatment coefficients in treatment group one (two): Hypertension: 0.00 (0.00). Diabetes: 0.00 (0.00). Cardiovascular diseases: 0.08 (0.21). Mental health: 0.00 (0.16). Original data from SISAB.

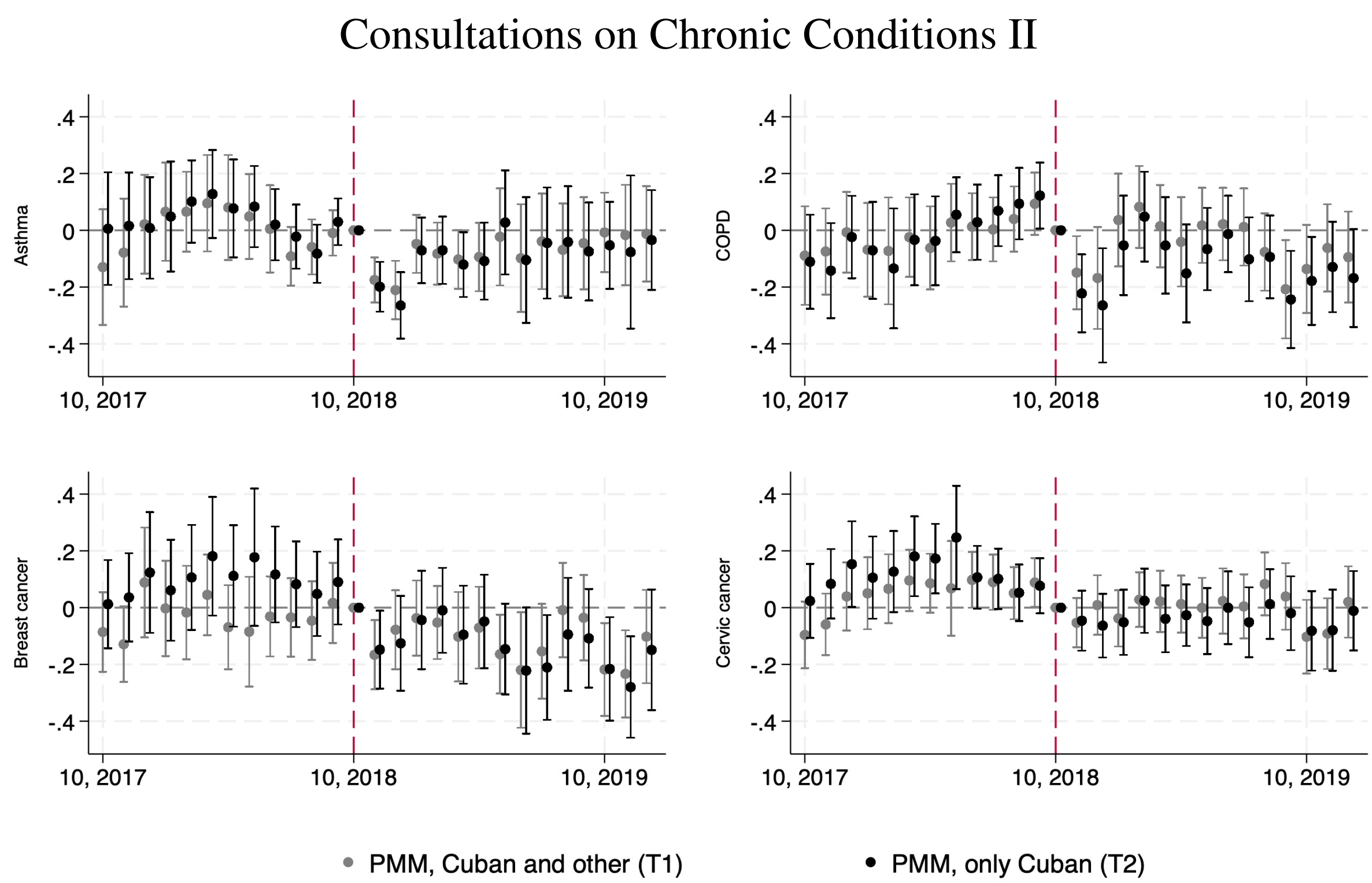

Figure B.2: The graph shows event studies for the number of primary care consultations on asthma, COPD, and breast and cervical cancer screenings, following equation 1. Regressions include municipality and state-by-time (month) fixed effects. Dependent variable measured per 100,000 inhabitants. The series in grey presents treatment group one, the series in black presents treatment group two. Whiskers around the point estimates present 95 percent confidence intervals, where standard errors are clustered at the municipality level. The dashed vertical lines present the pre-treatment month October 2018. Joint p-value of pre-treatment coefficients in treatment group one (two): Asthma: 0.06 (0.31). COPD: 0.48 (0.13). Breast cancer: 0.22 (0.93). Cervical cancer: 0.02 (0.30). Original data from SISAB.

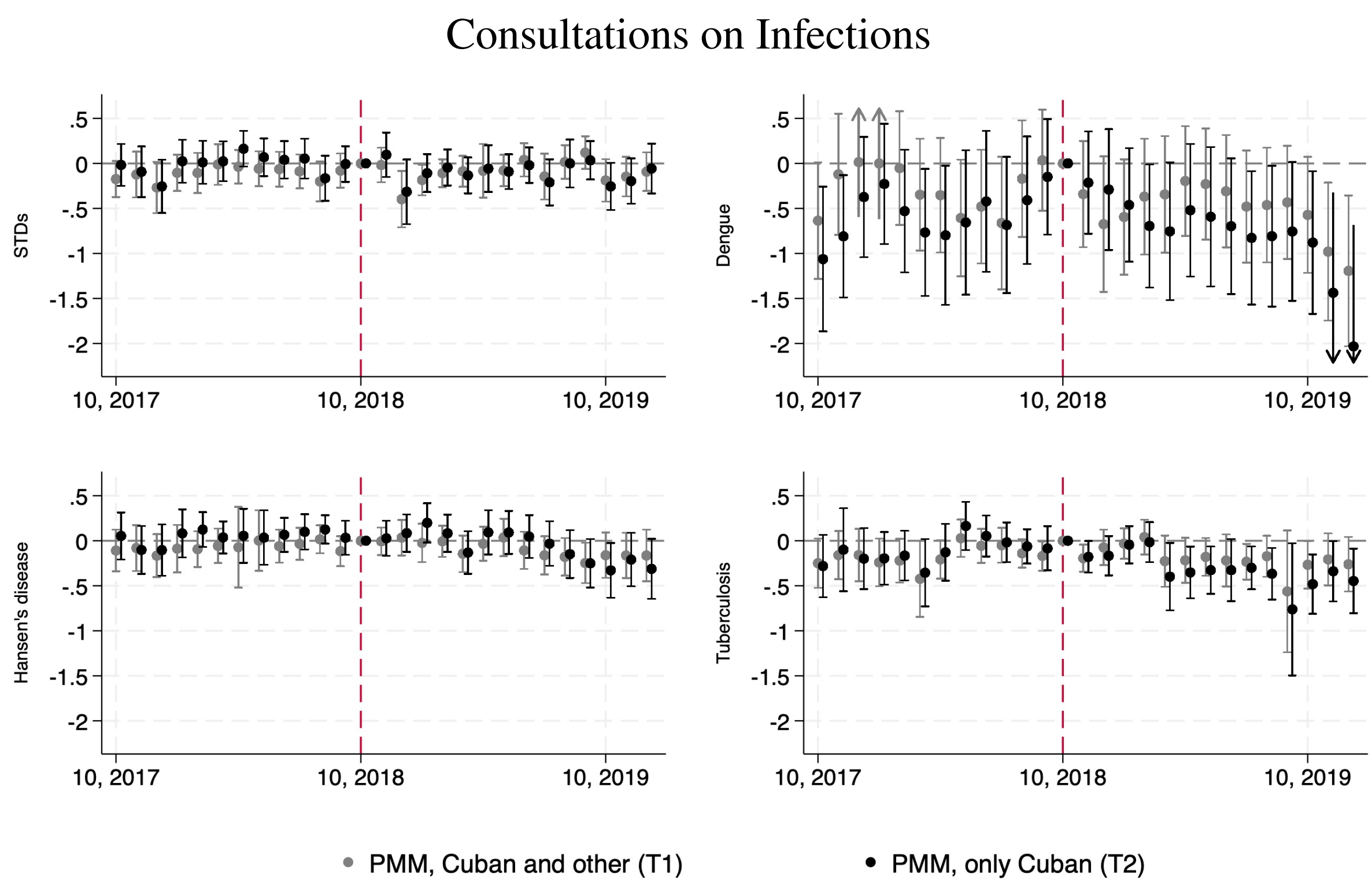

Figure B.3: The graph shows event studies for the number of primary care consultations on sexually transmitted diseases, dengue, Hansen's disease, and tuberculosis, following equation 1. Regressions include municipality and state-by-time (month) fixed effects. Dependent variable measured per 100,000 inhabitants. The series in grey presents treatment group one, the series in black presents treatment group two. Whiskers around the point estimates present 95 percent confidence intervals, where standard errors are clustered at the municipality level. The dashed vertical lines present the pre-treatment month October 2018. Joint p-value of pre-treatment coefficients in treatment group one (two): STDs: 0.65 (0.20). Dengue: 0.02 (0.02). Hansen's disease: 0.97 (0.56). Tuberculosis: 0.23 (0.44). Original data from SISAB.

Effects on Total Hospitalization and Total Mortality for Individuals Aged 60 and Above

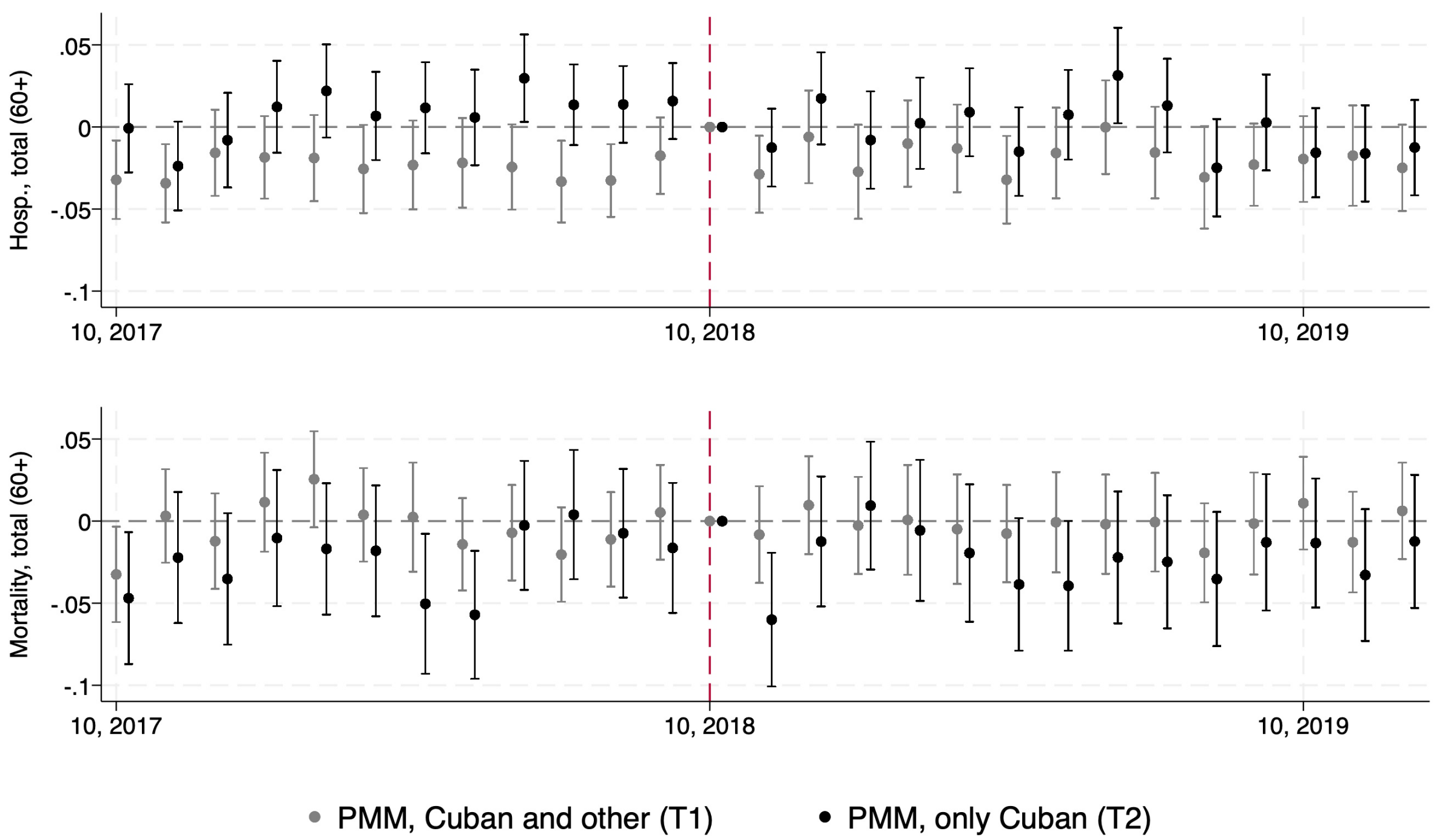

Figure B.4: The graph shows event studies for total hospitalizations among individuals aged 60 and above (upper panel) and total hospital mortality among individuals aged 60 and above (lower panel). Regressions follow equation (1) and include municipality and state-by-time (month) fixed effects. Dependent variable adjusted by population size. The series in grey presents treatment group one, the series in black presents treatment group two. Whiskers around the point estimates present 95 percent confidence intervals, where standard errors are clustered at the municipality level. The dashed vertical lines present the pre-treatment month October 2018. Joint p-value of pre-treatment coefficients in treatment group one (two): Total hospitalizations (60+): 0.18 (0.06). Total hospital mortality (60+): 0.02 (0.04). Original data from SIH/Datasus and SIM/Datasus.

C Robustness

C.1 Time-Varying Controls

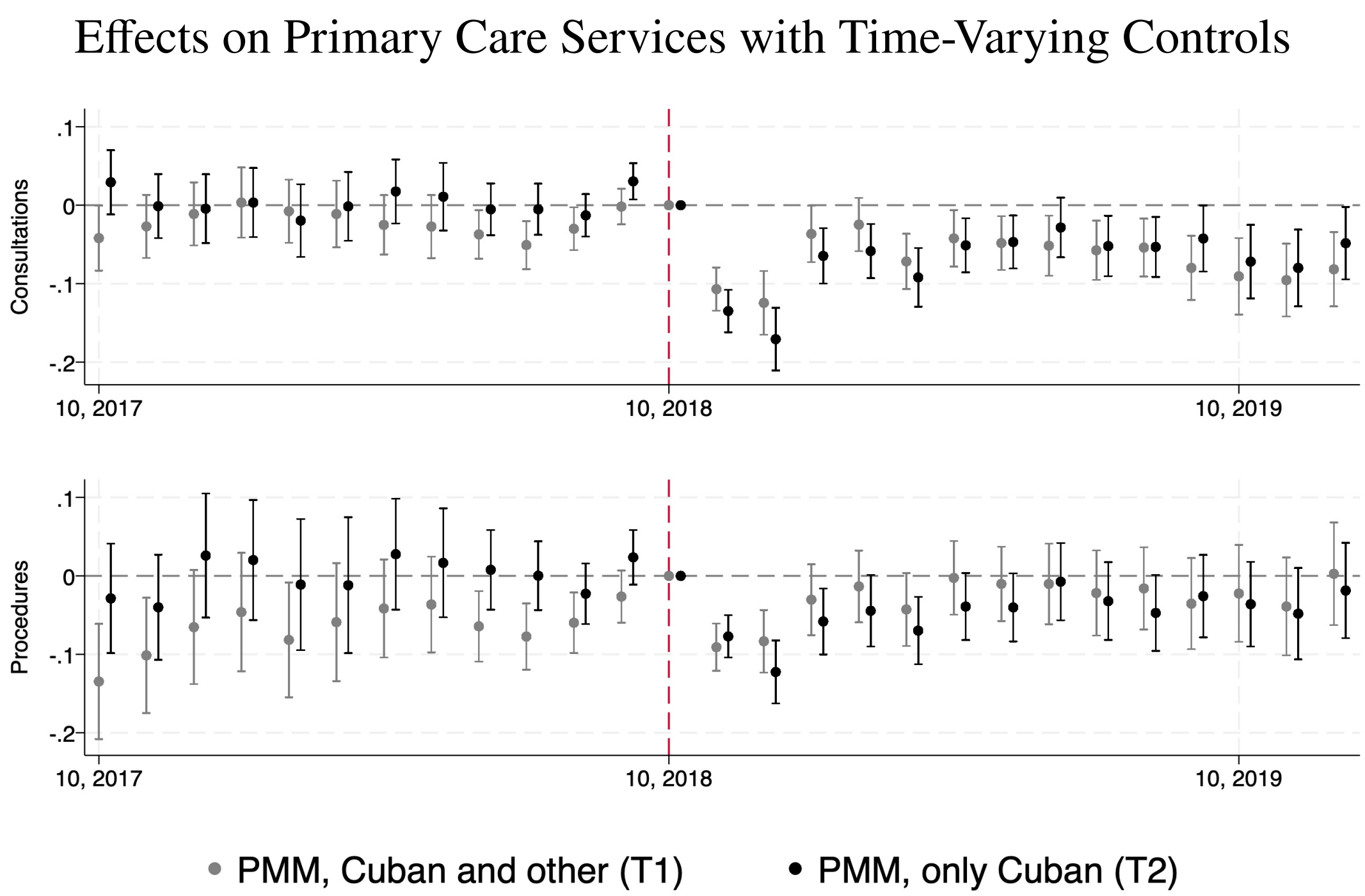

Figure C.1: The graph shows event studies for the number of consultations (upper panel) and procedures (lower panel) performed in primary health care facilities, following equation 1. Regressions include municipality and state-by-time (month) fixed effects, together with annual municipality-level controls (population share of Bolsa Familia coverage, Bolsa Familia expenses per capita, GDP per capita, value added by public sector employment (in R$ 1,000), the share of the population above 65, and the sex ratio). Dependent variable measured per 100,000 inhabitants. Standard errors are clustered at the municipality level. Whiskers around the point estimates present 95 percent confidence intervals, where standard errors are clustered at the municipality level. The dashed vertical lines present the pre-treatment month October 2018. Joint p-value of pre-treatment coefficients in treatment group one (two): Consultations: 0.04 (0.01). Procedures: 0.00 (0.03). Original data from SISAB.

Effects on Primary Care Consultations with Time-Varying Controls

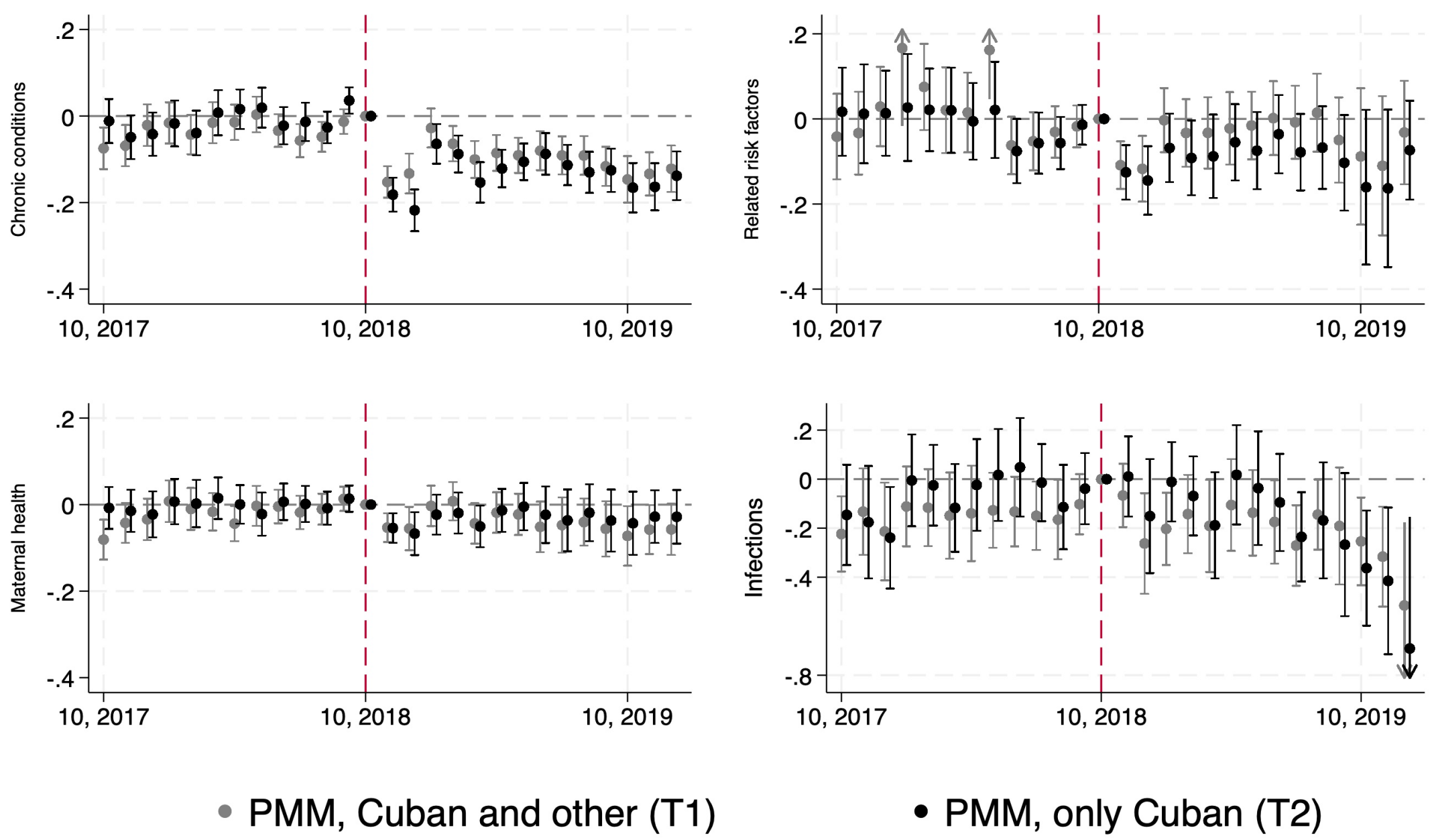

Figure C.2: The graph shows event studies for the number of consultations in four different categories, performed in primary health care facilities, following equation 1. Regressions include municipality and state-by-time (month) fixed effects, together with annual municipality-level controls (population share of Bolsa Familia coverage, Bolsa Familia expenses per capita, GDP per capita, value added by public sector employment (in R$ 1,000), the share of the population above 65, and the sex ratio). Dependent variable measured per 100,000 inhabitants. Standard errors are clustered at the municipality level. Whiskers around the point estimates present 95 percent confidence intervals, where standard errors are clustered at the municipality level. The dashed vertical lines present the pre-treatment month October 2018. Joint p-value of pre-treatment coefficients in treatment group one (two): Chronic conditions: 0.00 (0.00). Related risk factors: 0.00 (0.51). Maternal health: 0.00 (0.81). Infections: 0.37 (0.28). Original data from SISAB.

Effects on Hospitalizations with Time-Varying Controls

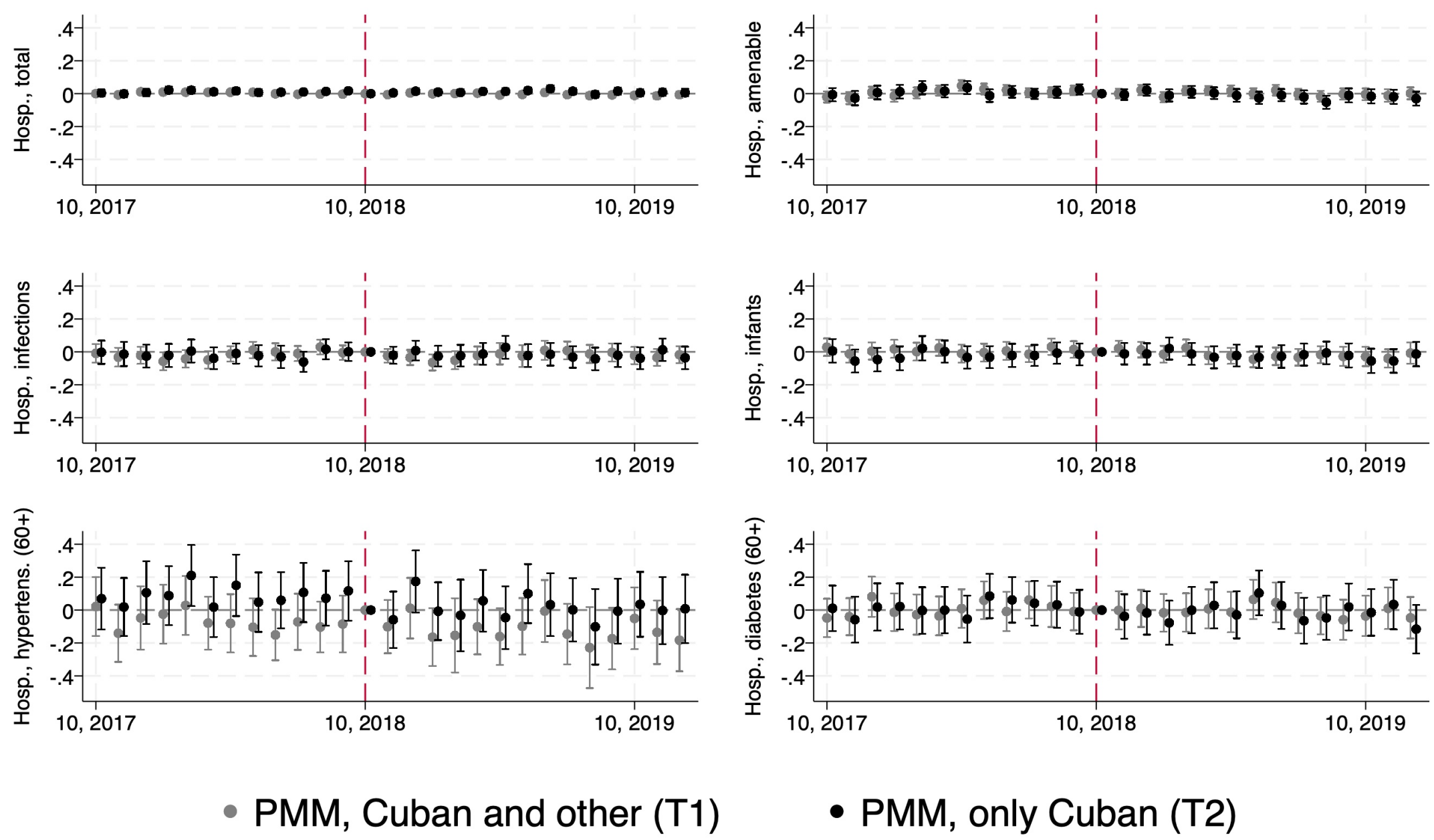

Figure C.3: The graph shows event studies for the number of hospitalizations in six different categories, following equation 1. Regressions include municipality and state-by-time (month) fixed effects, together with annual municipality-level controls (population share of Bolsa Familia coverage, Bolsa Familia expenses per capita, GDP per capita, value added by public sector employment (in R$ 1,000), the share of the population above 65, and the sex ratio). Dependent variable adjusted by population size. Standard errors are clustered at the municipality level. Whiskers around the point estimates present 95 percent confidence intervals, where standard errors are clustered at the municipality level. The dashed vertical lines present the pre-treatment month October 2018. Joint p-value of pre-treatment coefficients in treatment group one (two): Total hospitalizations: 0.28 (0.18). Amenable: 0.01 (0.05). Infections: 0.26 (0.42). Infants: 0.56 (0.63). Hypertension (60+): 0.19 (0.64). Diabetes (60+): 0.36 (0.75). Original data from SIH/Datasus.

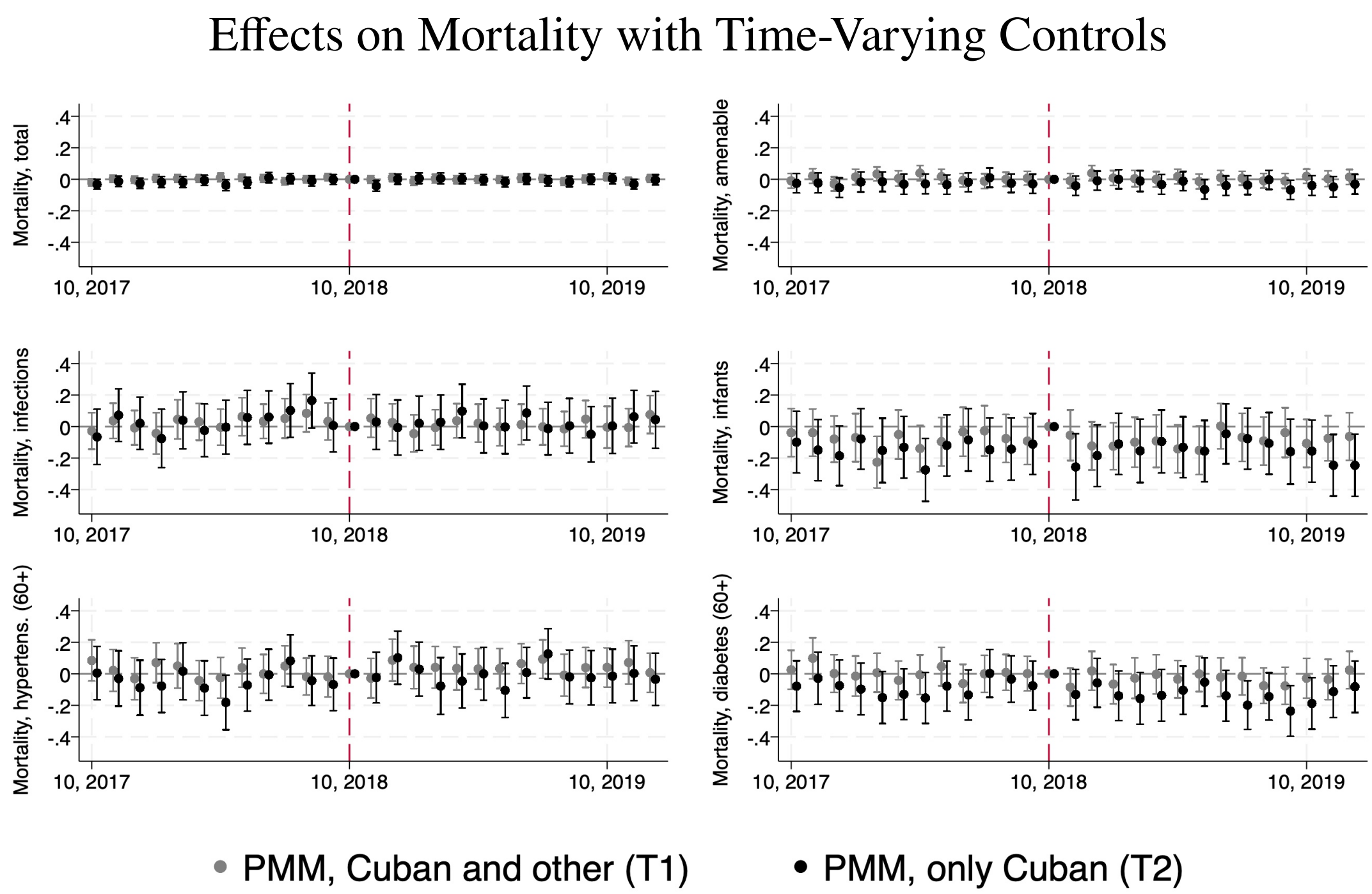

Figure C.4: The graph shows event studies for the number of hospital mortality in six different categories, following equation 1. Regressions include municipality and state-by-time (month) fixed effects, together with annual municipality-level controls (population share of Bolsa Familia coverage, Bolsa Familia expenses per capita, GDP per capita, value added by public sector employment (in R$ 1,000), the share of the population above 65, and the sex ratio). Dependent variable adjusted by population size. Standard errors are clustered at the municipality level. Whiskers around the point estimates present 95 percent confidence intervals, where standard errors are clustered at the municipality level. The dashed vertical lines present the pre-treatment month October 2018. Joint p-value of pre-treatment coefficients in treatment group one (two): Total hospital mortality: 0.25 (0.18). Amenable: 0.30 (0.90). Infections: 0.66 (0.20). Infants: 0.32 (0.58). Hypertension (60+): 0.64 (0.29). Diabetes (60+): 0.75 (0.63). Original data from SIM/Datasus.

C.2 Alternative Definition of the Control Group

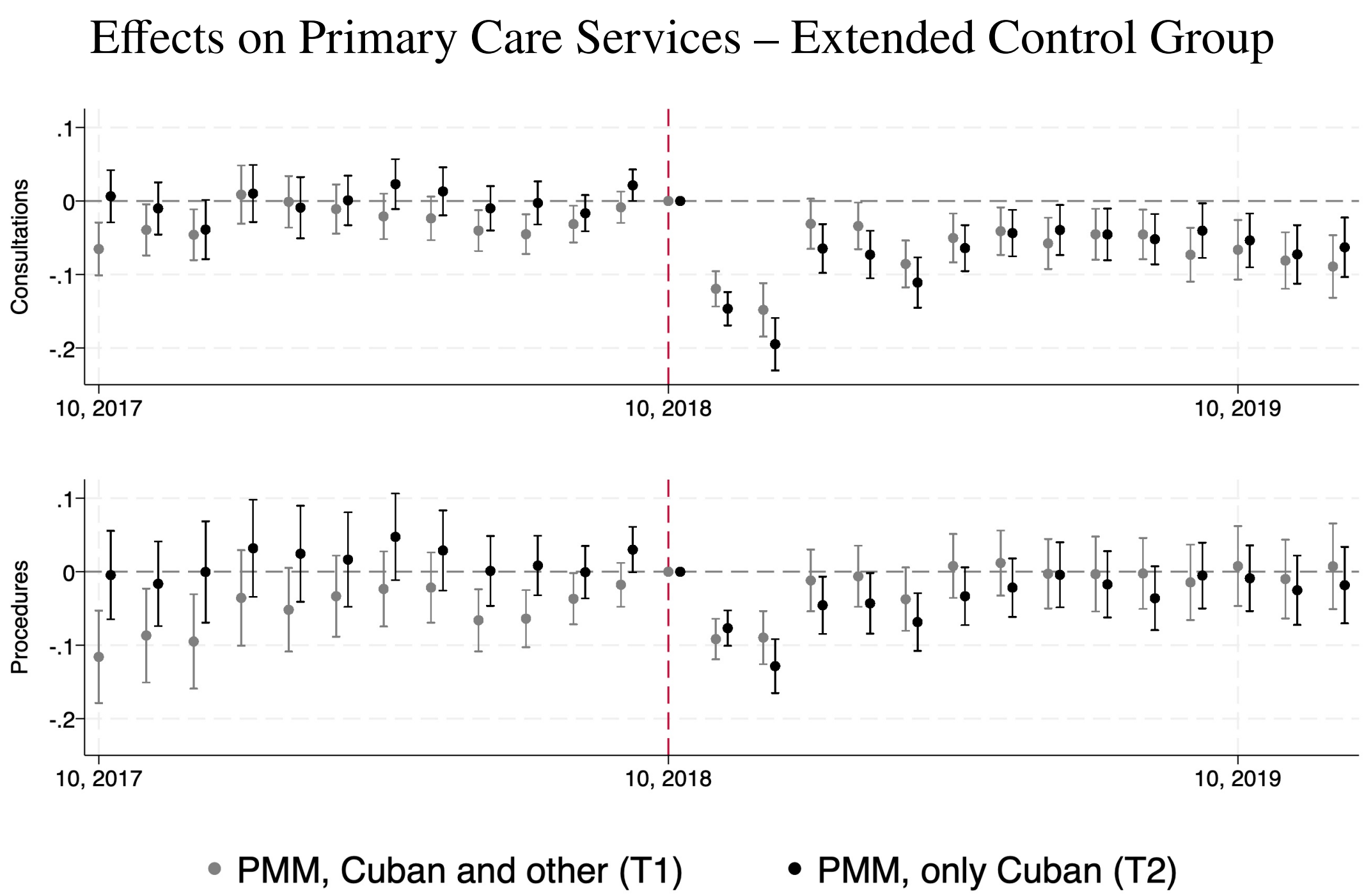

Figure C.5: The graph shows event studies for the number of consultations (upper panel) and procedures (lower panel) performed in primary health care facilities, following equation 1 but including non-PMM municipalities in the control group. Regressions include municipality and state-by-time (month) fixed effects. Dependent variable measured per 100,000 inhabitants. Standard errors are clustered at the municipality level. Whiskers around the point estimates present 95 percent confidence intervals, where standard errors are clustered at the municipality level. The dashed vertical lines present the pre-treatment month October 2018. Joint p-value of pre-treatment coefficients in treatment group one (two): Consultations: 0.00 (0.00). Procedures: 0.00 (0.09). Original data from SISAB.

Effects on Primary Care Consultations – Extended Control Group

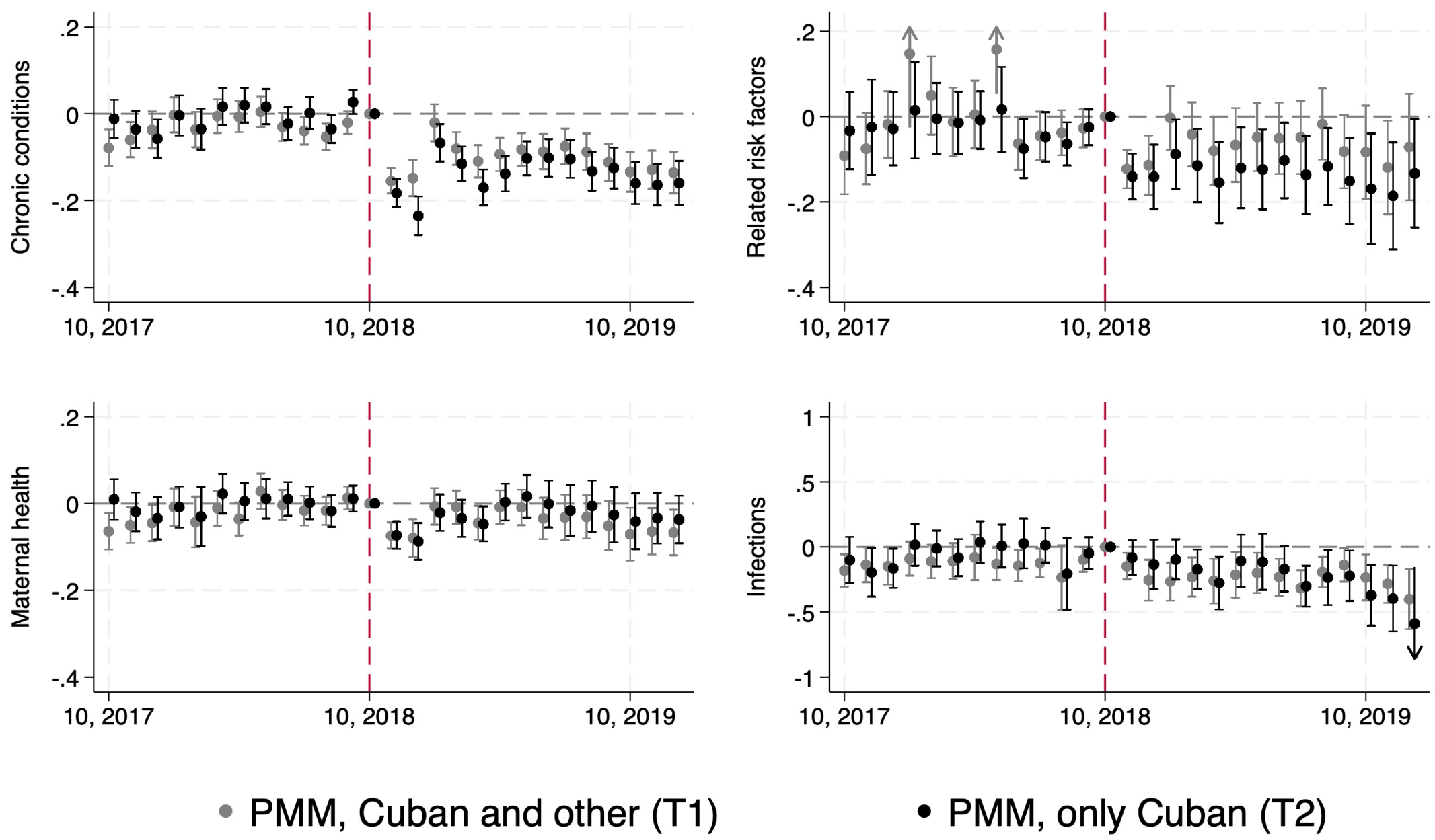

Figure C.6: The graph shows event studies for the number of consultations in four different categories, performed in primary health care facilities, following equation 1 but including non-PMM municipalities in the control group. Regressions include municipality and state-by-time (month) fixed effects. Dependent variable measured per 100,000 inhabitants. Standard errors are clustered at the municipality level. Whiskers around the point estimates present 95 percent confidence intervals, where standard errors are clustered at the municipality level. The dashed vertical lines present the pre-treatment month October 2018. Joint p-value of pre-treatment coefficients in treatment group one (two): Chronic conditions: 0.00 (0.00). Related risk factors: 0.00 (0.35). Maternal health: 0.00 (0.21). Infections: 0.41 (0.15). Original data from SISAB.

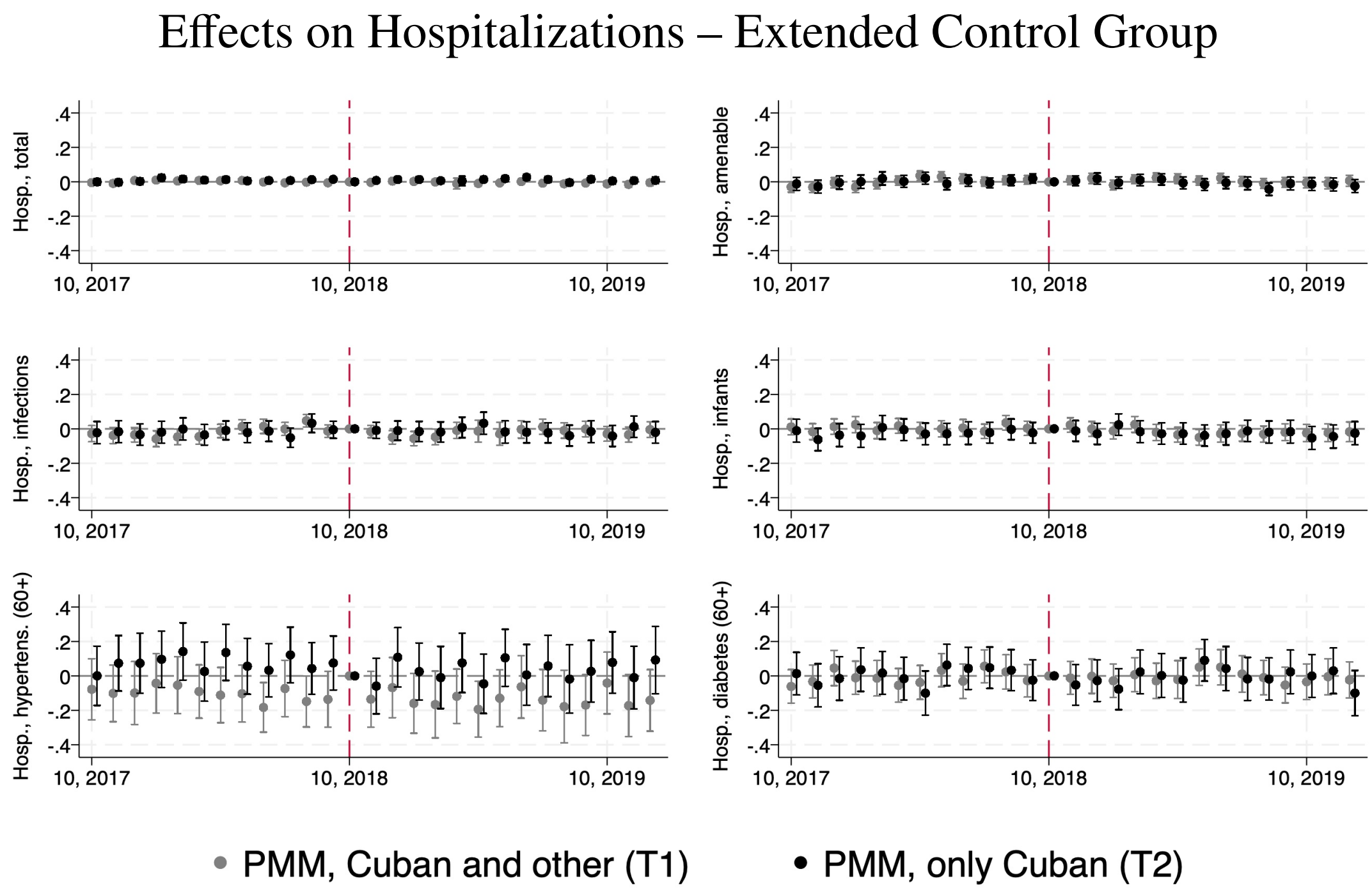

Figure C.7: The graph shows event studies for the number of hospitalizations in six different categories, following equation 1 but including non-PMM municipalities in the control group. Regressions include municipality and state-by-time (month) fixed effects. Dependent variable adjusted by population size. Standard errors are clustered at the municipality level. Whiskers around the point estimates present 95 percent confidence intervals, where standard errors are clustered at the municipality level. The dashed vertical lines present the pre-treatment month October 2018. Joint p-value of pre-treatment coefficients in treatment group one (two): Total hospitalizations: 0.06 (0.09). Amenable: 0.00 (0.16). Infections: 0.00 (0.23). Infants: 0.32 (0.78). Hypertension (60+): 0.32 (0.74). Diabetes (60+): 0.19 (0.40). Original data from SIH/Datasus.

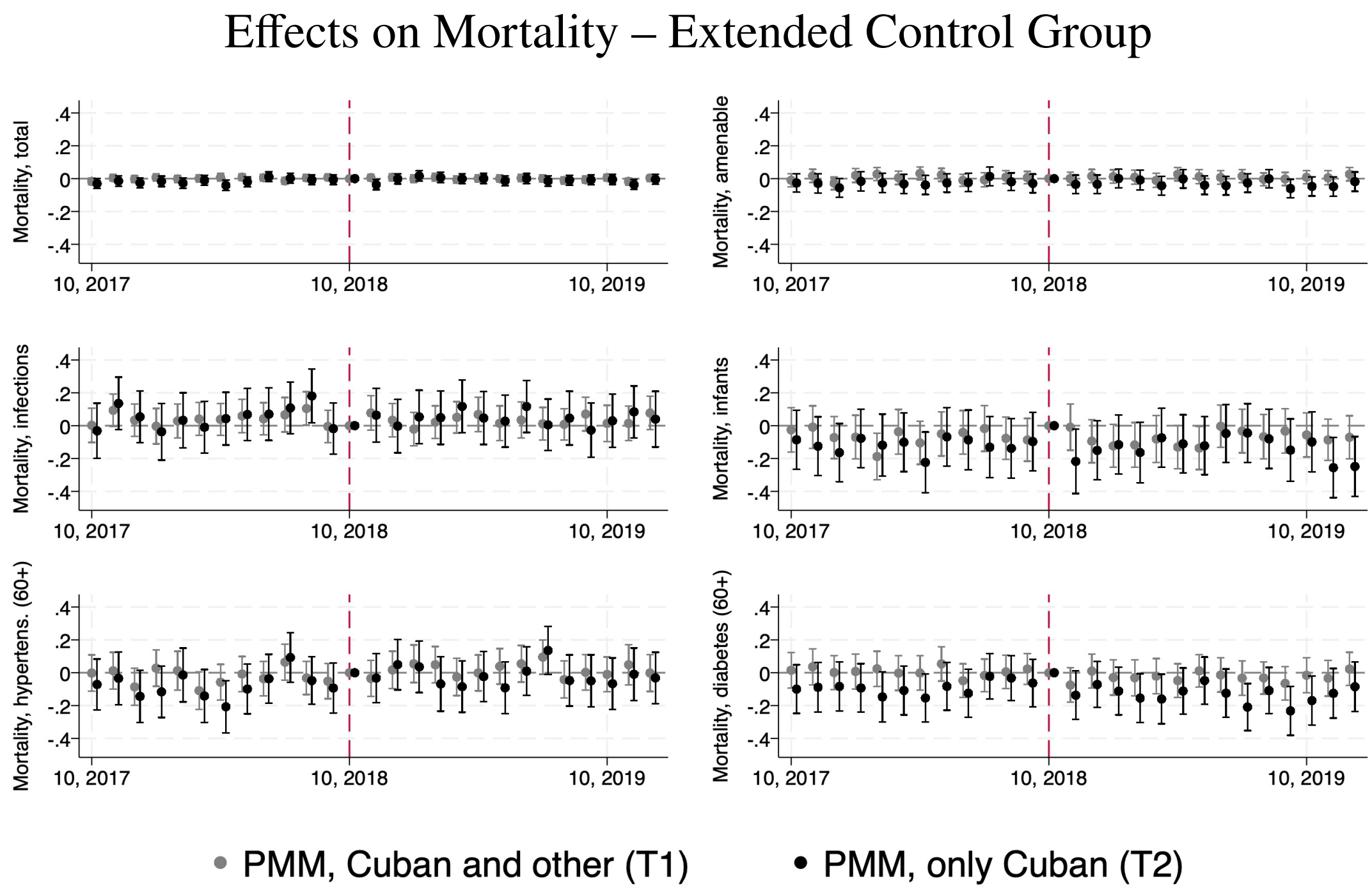

Figure C.8: The graph shows event studies for the number of hospital mortality in six different categories, following equation 1 but including non-PMM municipalities in the control group. Regressions include municipality and state-by-time (month) fixed effects. Dependent variable adjusted by population size. Standard errors are clustered at the municipality level. Whiskers around the point estimates present 95 percent confidence intervals, where standard errors are clustered at the municipality level. The dashed vertical lines present the pre-treatment month October 2018. Joint p-value of pre-treatment coefficients in treatment group one (two): Total hospital mortality: 0.34 (0.06). Amenable: 0.15 (0.72). Infections: 0.47 (0.11). Infants: 0.37 (0.74). Hypertension (60+): 0.21 (0.02). Diabetes (60+): 0.94 (0.73). Original data from SIM/Datasus.

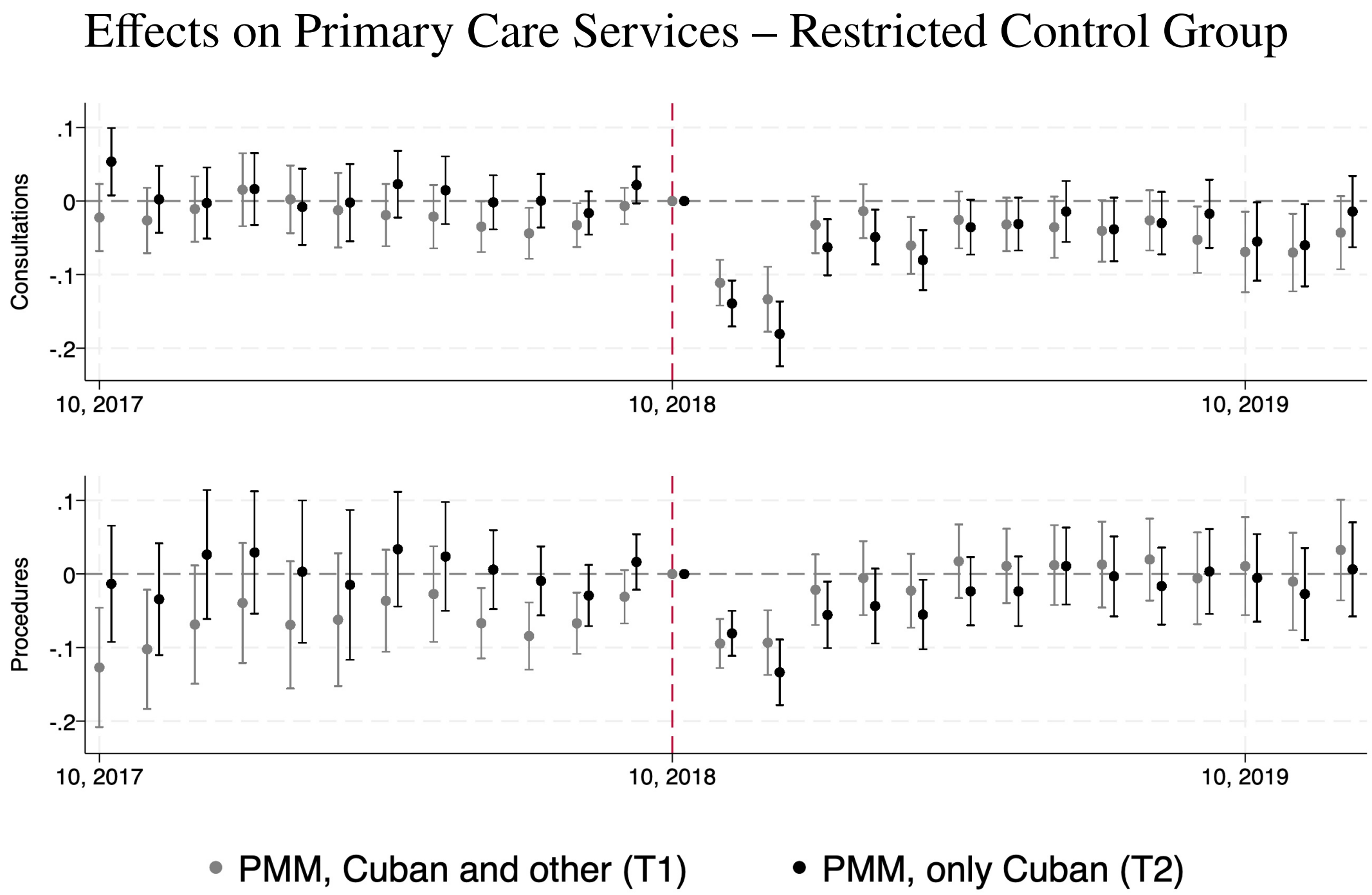

Figure C.9: The graph shows event studies for the number of consultations (upper panel) and procedures (lower panel) performed in primary health care facilities, following equation 1 but excluding municipalities which were formally part of PMM but did not employ any PMM doctor in October 2018 from the control group. Regressions include municipality and state-by-time (month) fixed effects. Dependent variable measured per 100,000 inhabitants. Standard errors are clustered at the municipality level. Whiskers around the point estimates present 95 percent confidence intervals, where standard errors are clustered at the municipality level. The dashed vertical lines present the pre-treatment month October 2018. Joint p-value of pre-treatment coefficients in treatment group one (two): Consultations: 0.27 (0.00). Procedures: 0.00 (0.04). Original data from SISAB.

Effects on Primary Care Consultations – Restricted Control Group

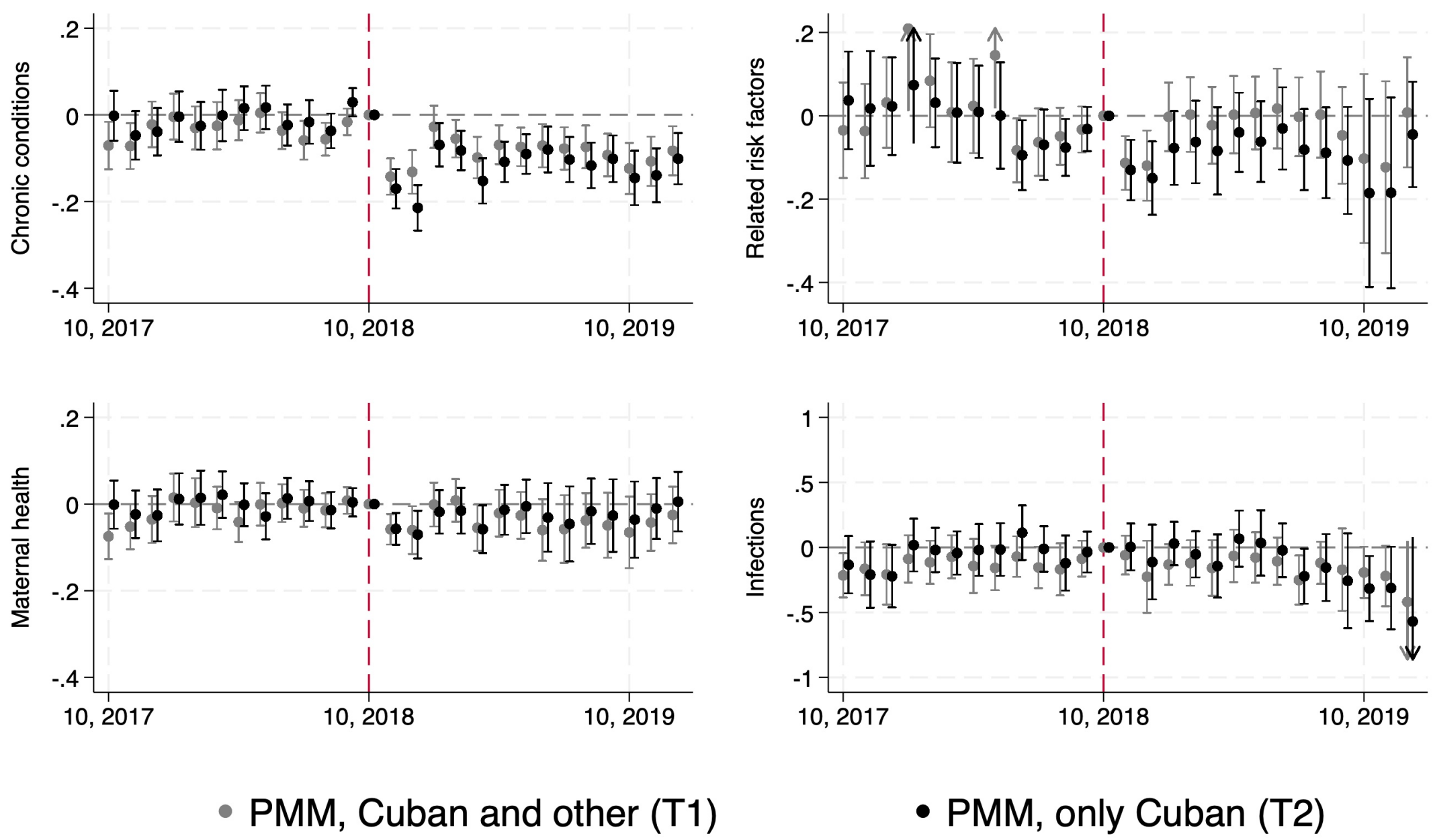

Figure C.10: The graph shows event studies for the number of consultations in four different categories, performed in primary health care facilities, following equation 1 but excluding municipalities which were formally part of PMM but did not employ any PMM doctor in October 2018 from the control group. Regressions include municipality and state-by-time (month) fixed effects. Dependent variable measured per 100,000 inhabitants. Standard errors are clustered at the municipality level. Whiskers around the point estimates present 95 percent confidence intervals, where standard errors are clustered at the municipality level. The dashed vertical lines present the pre-treatment month October 2018. Joint p-value of pre-treatment coefficients in treatment group one (two): Chronic conditions: 0.00 (0.00). Related risk factors: 0.00 (0.22). Maternal health: 0.01 (0.51). Infections: 0.45 (0.52). Original data from SISAB.

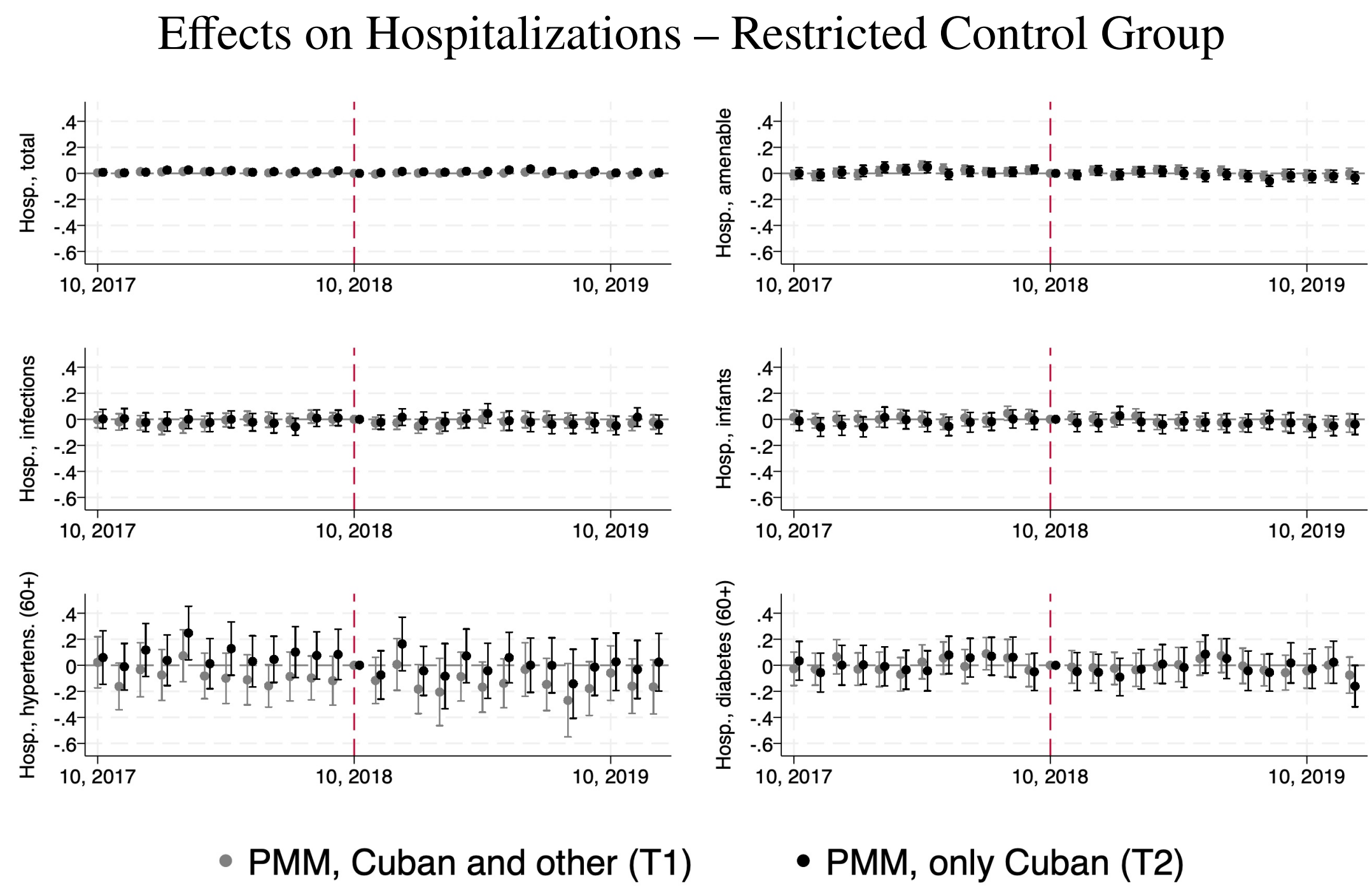

Figure C.11: The graph shows event studies for the number of hospitalizations in six different categories, following equation 1 but excluding municipalities which were formally part of PMM but did not employ any PMM doctor in October 2018 from the control group. Regressions include municipality and state-by-time (month) fixed effects. Dependent variable adjusted by population size. Standard errors are clustered at the municipality level. Whiskers around the point estimates present 95 percent confidence intervals, where standard errors are clustered at the municipality level. The dashed vertical lines present the pre-treatment month October 2018. Joint p-value of pre-treatment coefficients in treatment group one (two): Total hospitalizations: 0.46 (0.13). Amenable: 0.01 (0.06). Infections: 0.66 (0.74). Infants: 0.41 (0.48). Hypertension (60+): 0.11 (0.50). Diabetes (60+): 0.25 (0.60). Original data from SIH/Datasus.

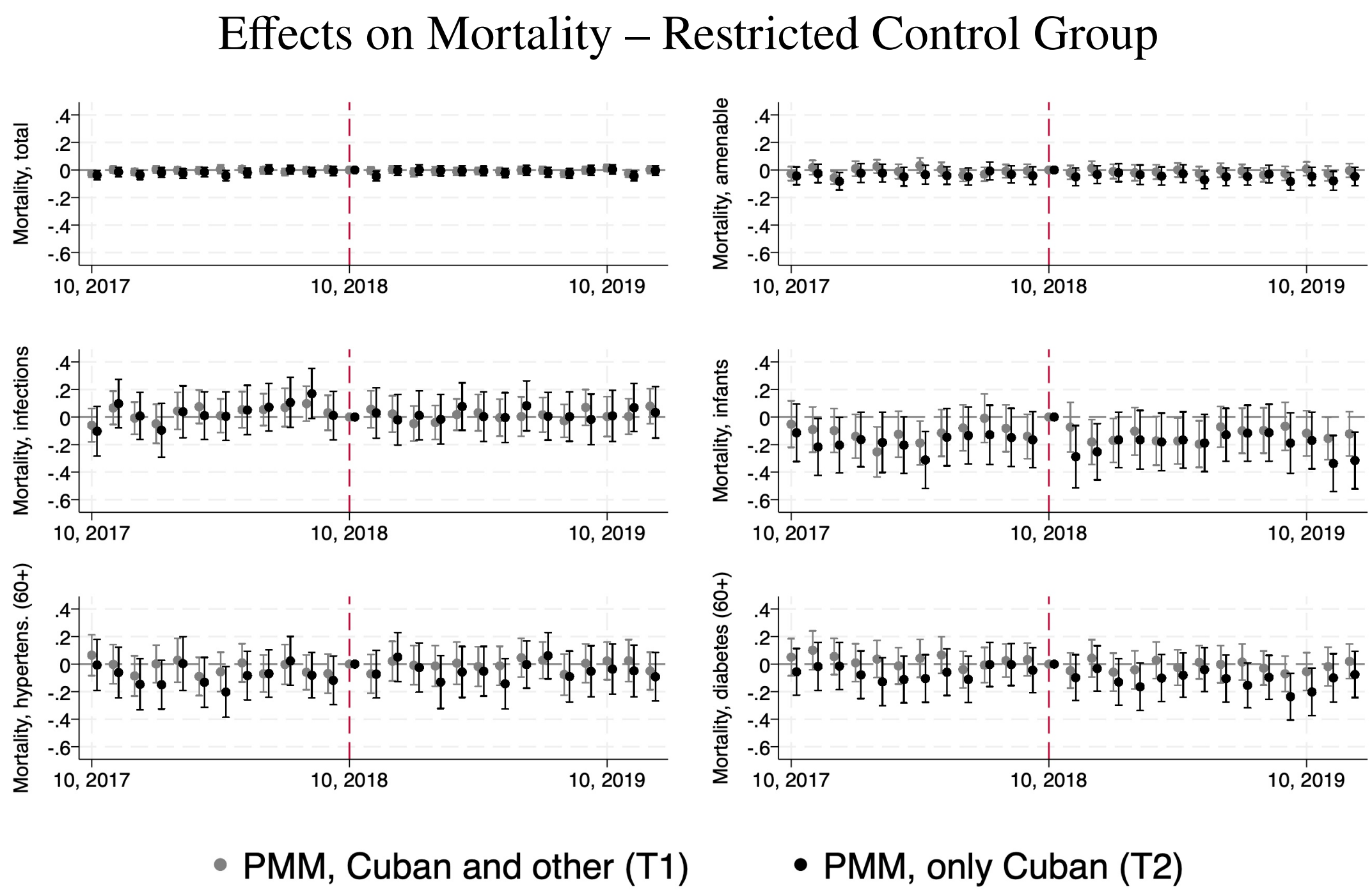

Figure C.12: The graph shows event studies for the number of hospital mortality in six different categories, following equation 1 but excluding municipalities which were formally part of PMM but did not employ any PMM doctor in October 2018 from the control group. Regressions include municipality and state-by-time (month) fixed effects. Dependent variable adjusted by population size. Standard errors are clustered at the municipality level. Whiskers around the point estimates present 95 percent confidence intervals, where standard errors are clustered at the municipality level. The dashed vertical lines present the pre-treatment month October 2018. Joint p-value of pre-treatment coefficients in treatment group one (two): Total hospital mortality: 0.37 (0.16). Amenable: 0.01 (0.57). Infections: 0.27 (0.11). Infants: 0.11 (0.47). Hypertension (60+): 0.46 (0.31). Diabetes (60+): 0.85 (0.90). Original data from SIM/Datasus.

C.3 Alternative Definitions of Treatment Variables and Specifications

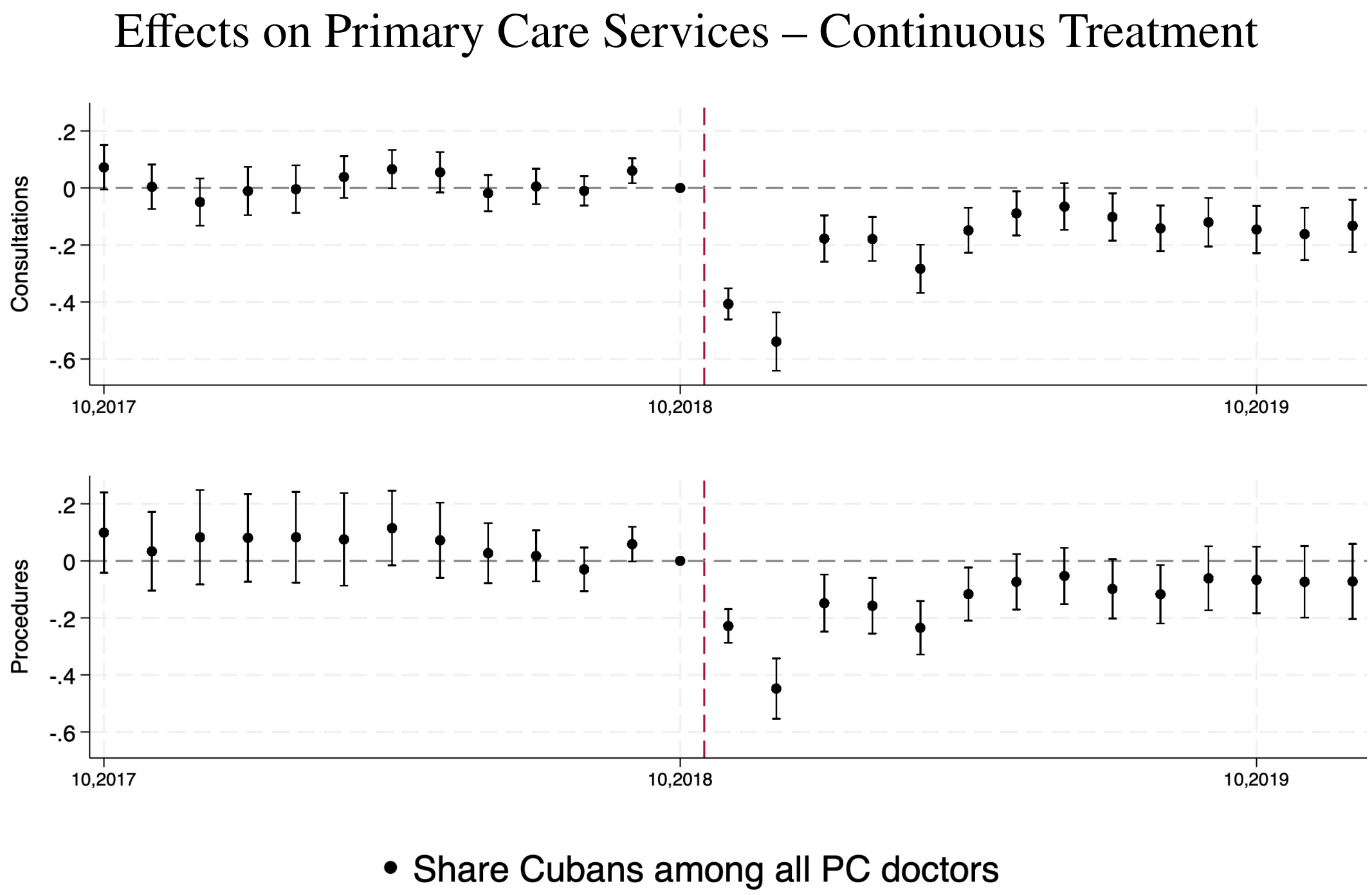

Figure C.13: The graph shows event studies for the number of consultations (upper panel) and procedures (lower panel) performed in primary health care facilities, following equation 2, where the treatment variable is defined as the share of Cubans among all municipal primary care doctors in October 2018 (PMM and non-PMM). Regressions include municipality and state-by-time (month) fixed effects. Dependent variable measured per 100,000 inhabitants. Whiskers around the point estimates present 95 percent confidence intervals, where standard errors are clustered at the municipality level. The dashed vertical lines present the pre-treatment month October 2018. Joint p-value of pre-treatment coefficients: Consultations: 0.00. Procedures: 0.10. Original data from SISAB.

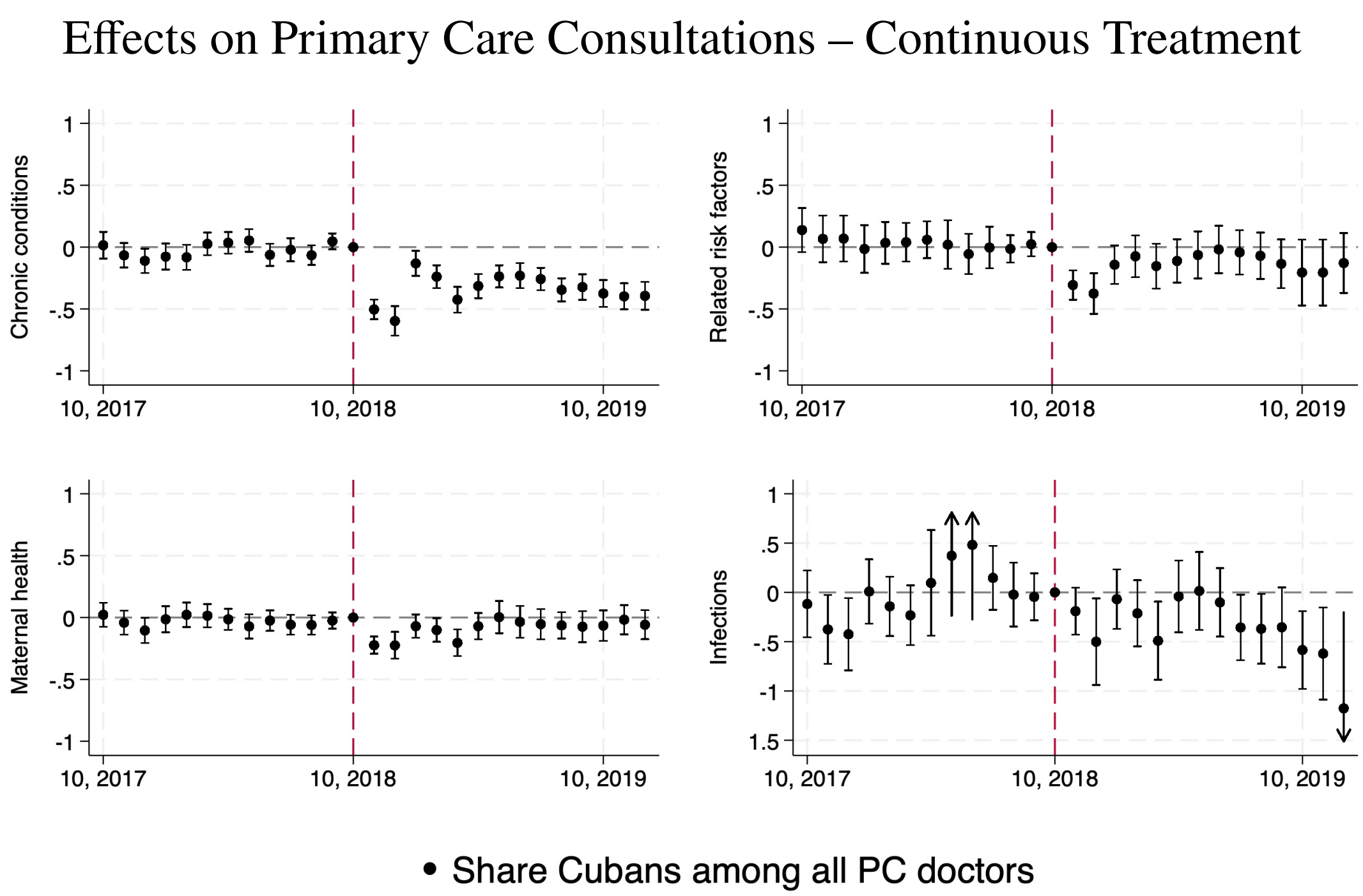

Figure C.14: The graph shows event studies for the number of consultations in four different primary care categories performed in primary health care facilities, following equation 2, where the treatment variable is defined as the share of Cubans among all municipal primary care doctors in October 2018 (PMM and non-PMM). Regressions include municipality and state-by-time (month) fixed effects. Dependent variable measured per 100,000 inhabitants. Whiskers around the point estimates present 95 percent confidence intervals, where standard errors are clustered at the municipality level. The dashed vertical lines present the pre-treatment month October 2018. Joint p-value of pre-treatment coefficients: 0.00. Related risk factors: 0.72. Maternal health: 0.12. Infections: 0.09. Original data from SISAB.

Effects on Hospitalizations – Continuous Treatment

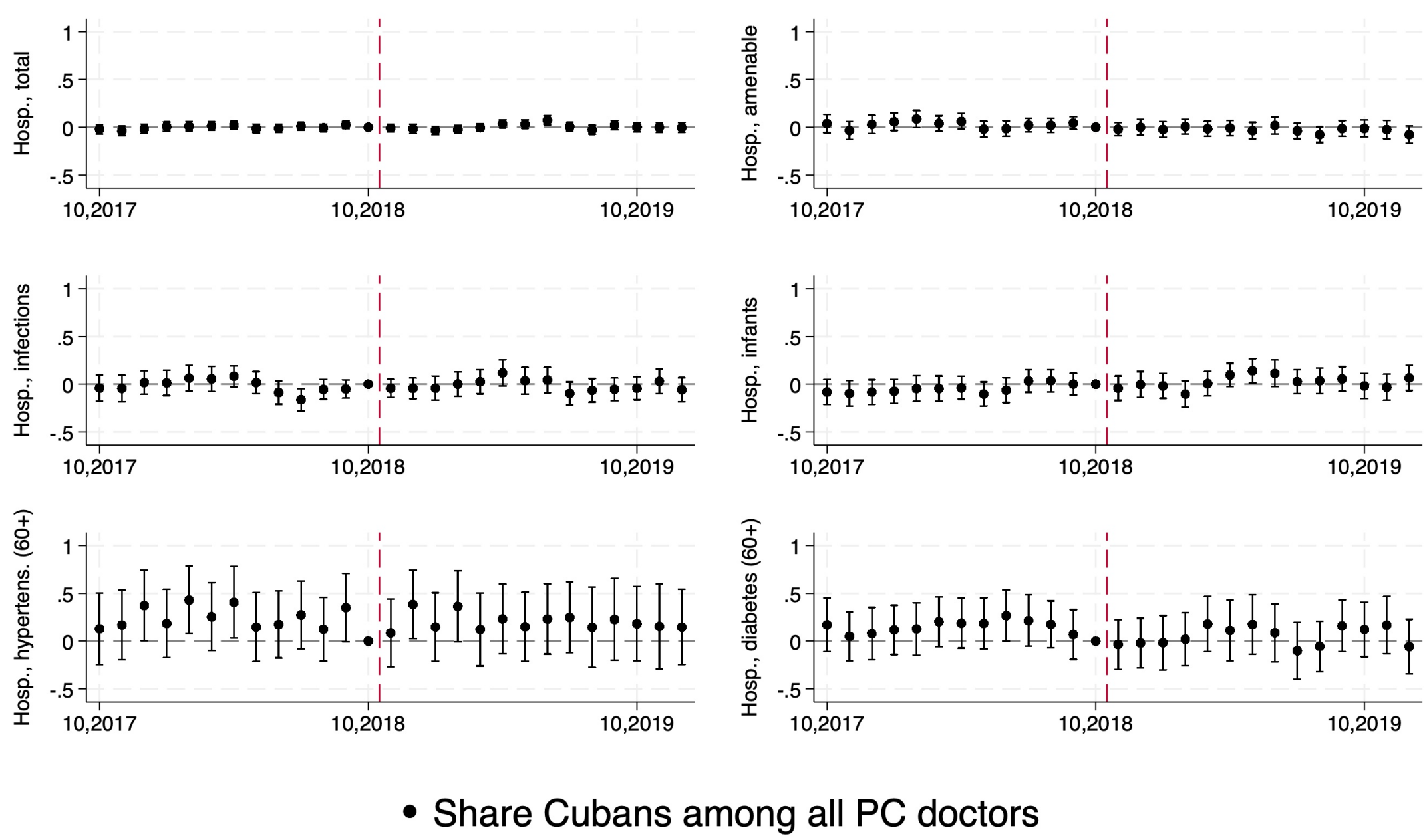

Figure C.15: The graph shows event studies for the number of hospitalizations in six different categories, following equation 2, where the treatment variable is defined as the share of Cubans among all municipal primary care doctors in October 2018 (PMM and non-PMM). Where indicated, the number of hospitalizations only includes individuals aged 60 or more. Regressions include municipality and state-by-time (month) fixed effects. Dependent variable adjusted by population size. The series in grey presents treatment group 1, the series in black presents treatment group 2. Whiskers around the point estimates present 95 percent confidence intervals, where standard errors are clustered at the municipality level. The dashed vertical lines present the pre-treatment month October 2018. Joint p-value of pre-treatment coefficients: Total hospitalizations: 0.05. Amenable: 0.03. Infections: 0.03. Infants: 0.59. Hypertension (60+): 0.28. Diabetes (60+): 0.79. Original data from SIH/Datasus.

Effects on Mortality – Continuous Treatment

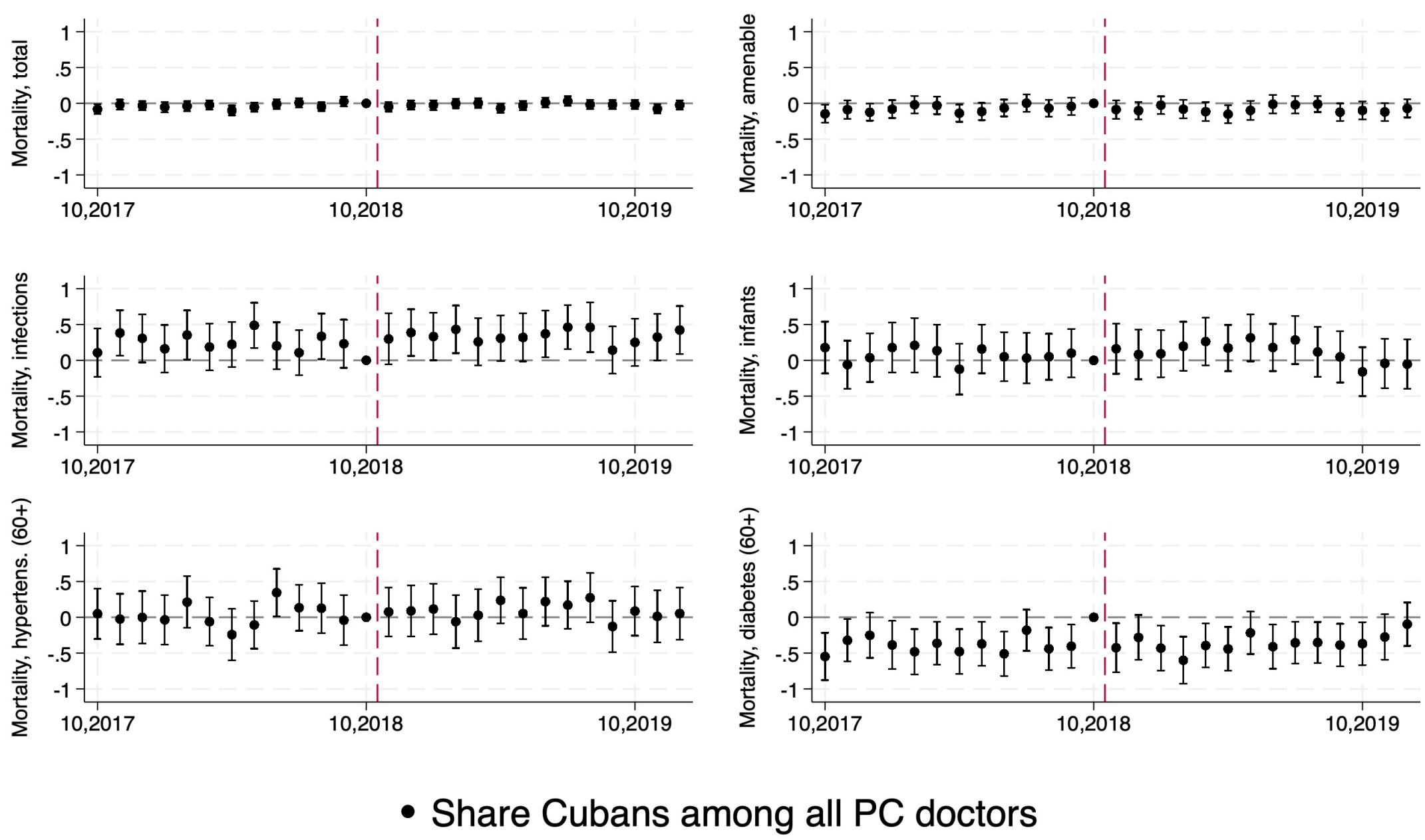

Figure C.16: The graph shows event studies for the number of hospital mortality in six different categories, following equation 2, where the treatment variable is defined as the share of Cubans among all municipal primary care doctors in October 2018 (PMM and non-PMM). Where indicated, the number of hospital mortality only includes individuals aged 60 or more. Regressions include municipality and state-by-time (month) fixed effects. Dependent variable adjusted by population size. The series in grey presents treatment group 1, the series in black presents treatment group 2. Whiskers around the point estimates present 95 percent confidence intervals, where standard errors are clustered at the municipality level. The dashed vertical lines present the pre-treatment month October 2018. Joint p-value of pre-treatment coefficients: Total hospital mortality: 0.01. Amenable: 0.26. Infections: 0.20. Infants: 0.73. Hypertension (60+): 0.17. Diabetes (60+): 0.06. Original data from SIM/Datasus.

C.4 Leave-One-Out Analysis

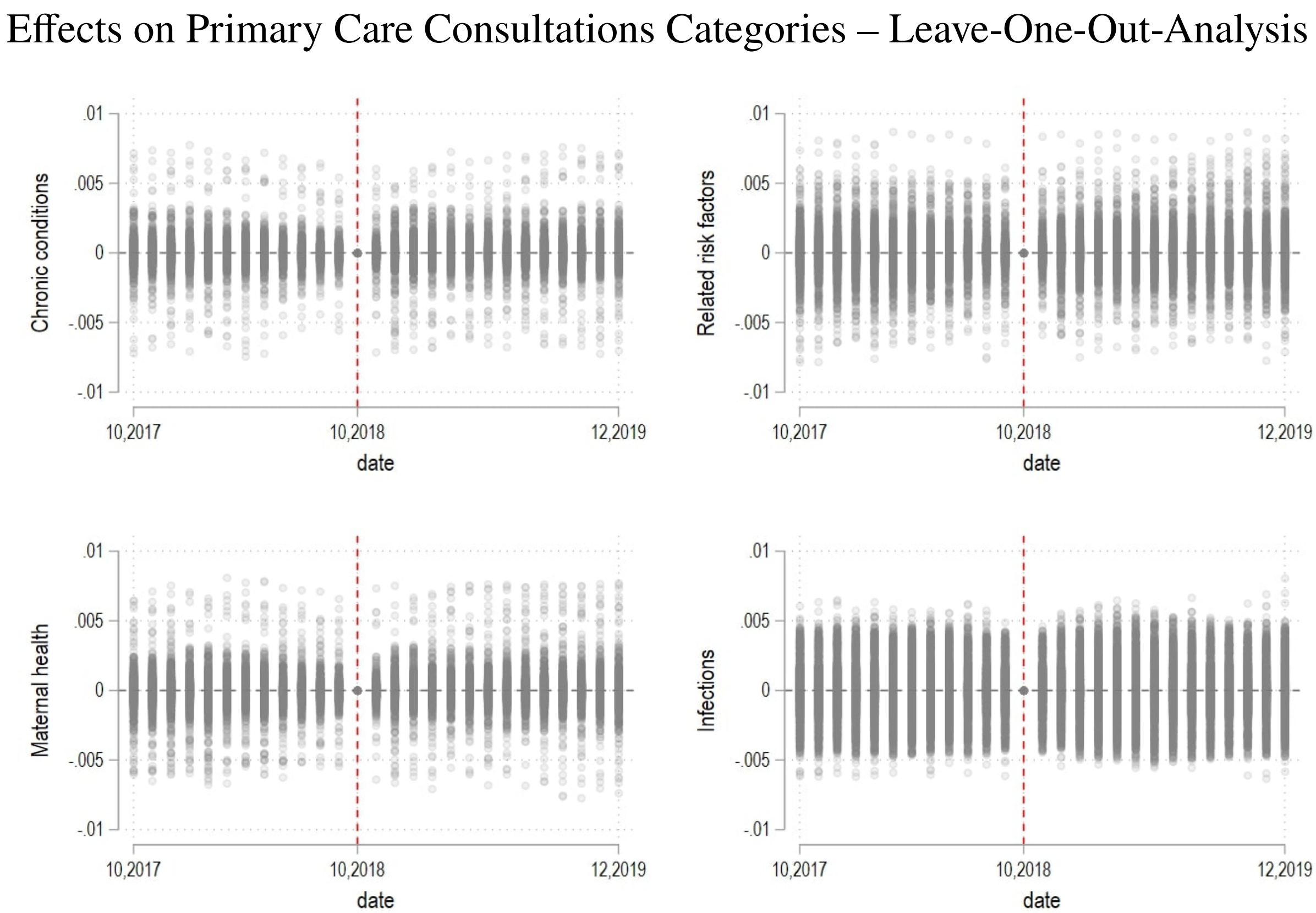

Figure C.17: The graph shows the differences between the estimated coefficients from the full event study and the leave-one-out analysis for the number of consultations performed in primary health care facilities in four different primary care categories. We follow equation 1, but use a binary treatment variable instead of a categorical one. Regressions include municipality and state-by-time (month) fixed effects. Dependent variable measured per 100,000 inhabitants. The dashed vertical lines present the pre-treatment month October 2018. Original data from SISAB.

D Discussion on Mechanisms and Health Systems Response: Additional Results

D.1 What Changed Within Primary Care Centers?

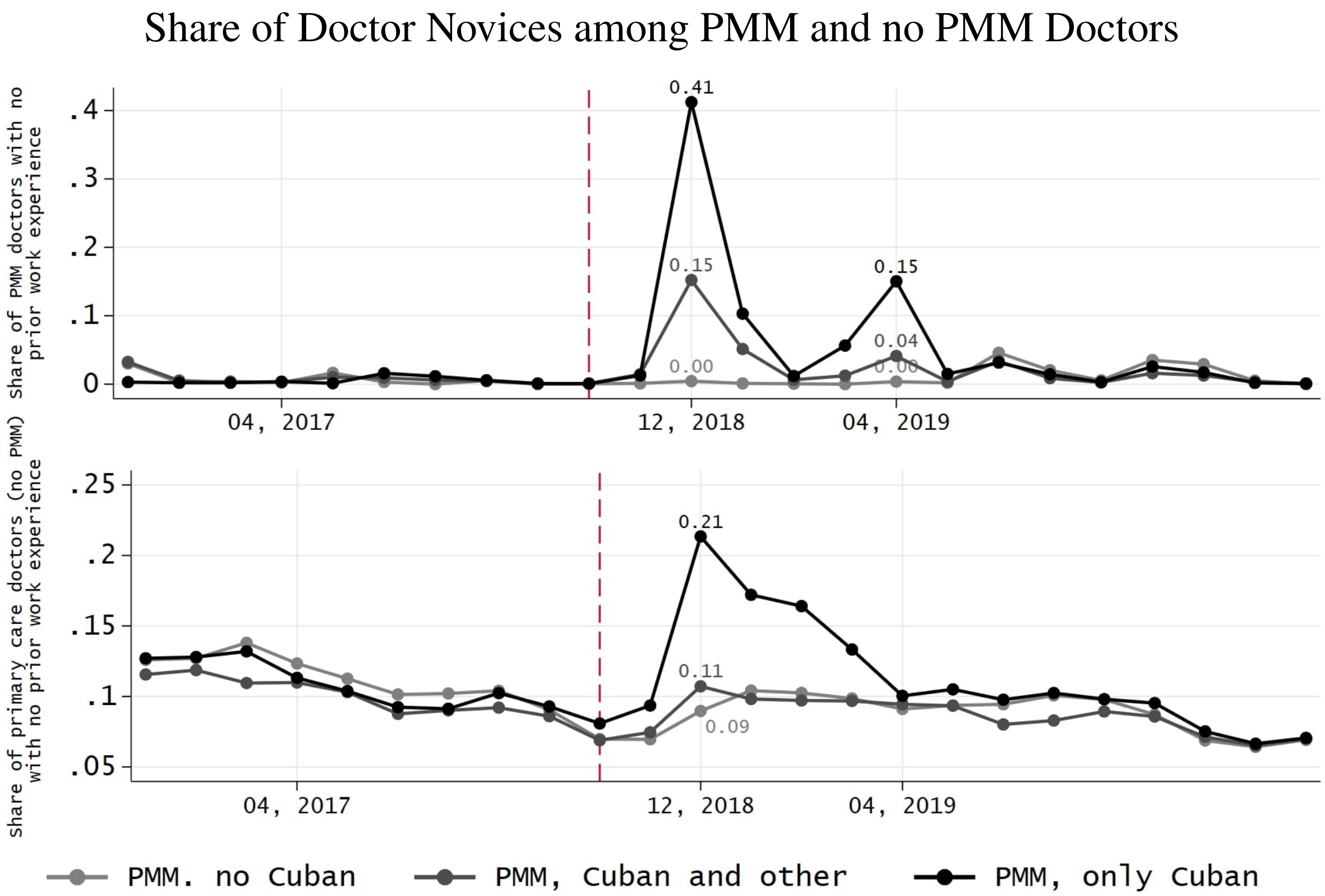

Figure D.1: The graph shows the share of PMM doctors that appear in CNES for the first time, calculated on the municipality-month level for all PMM-participating municipalities. The dashed vertical lines present the pre-treatment month October 2018. Original data from the Ministry of Health and CNES.

Effects on the Number of New Doctors and Doctors from non-PMM Municipalities

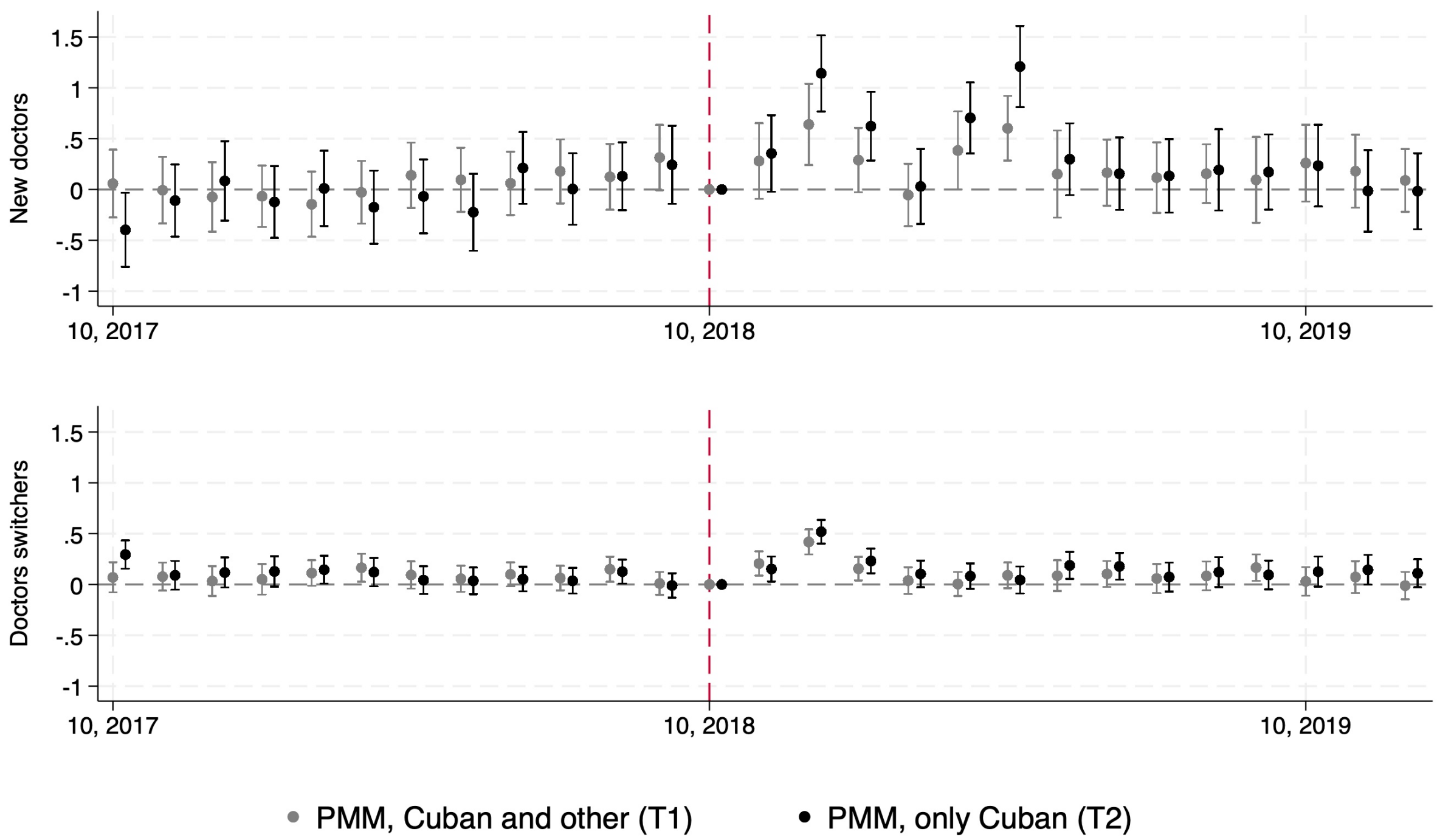

Figure D.2: The graph shows event studies for the number of primary care doctors that appear in CNES for the first time (upper panel) and the number of doctors switching from non-PMM to PMM municipalities (lower panel). These are PPML regressions that follow equation (1) and include municipality and state-by-time (month) fixed effects. Dependent variable adjusted by population size. The series in grey presents treatment group one, the series in black presents treatment group two. Whiskers around the point estimates present 95 percent confidence intervals, where standard errors are clustered at the municipality level. The dashed vertical lines present the pre-treatment month October 2018. Joint p-value of pre-treatment coefficients in treatment group one (two): New doctors: 0.14 (0.00). Switchers: 0.26 (0.00). Original data from CNES.

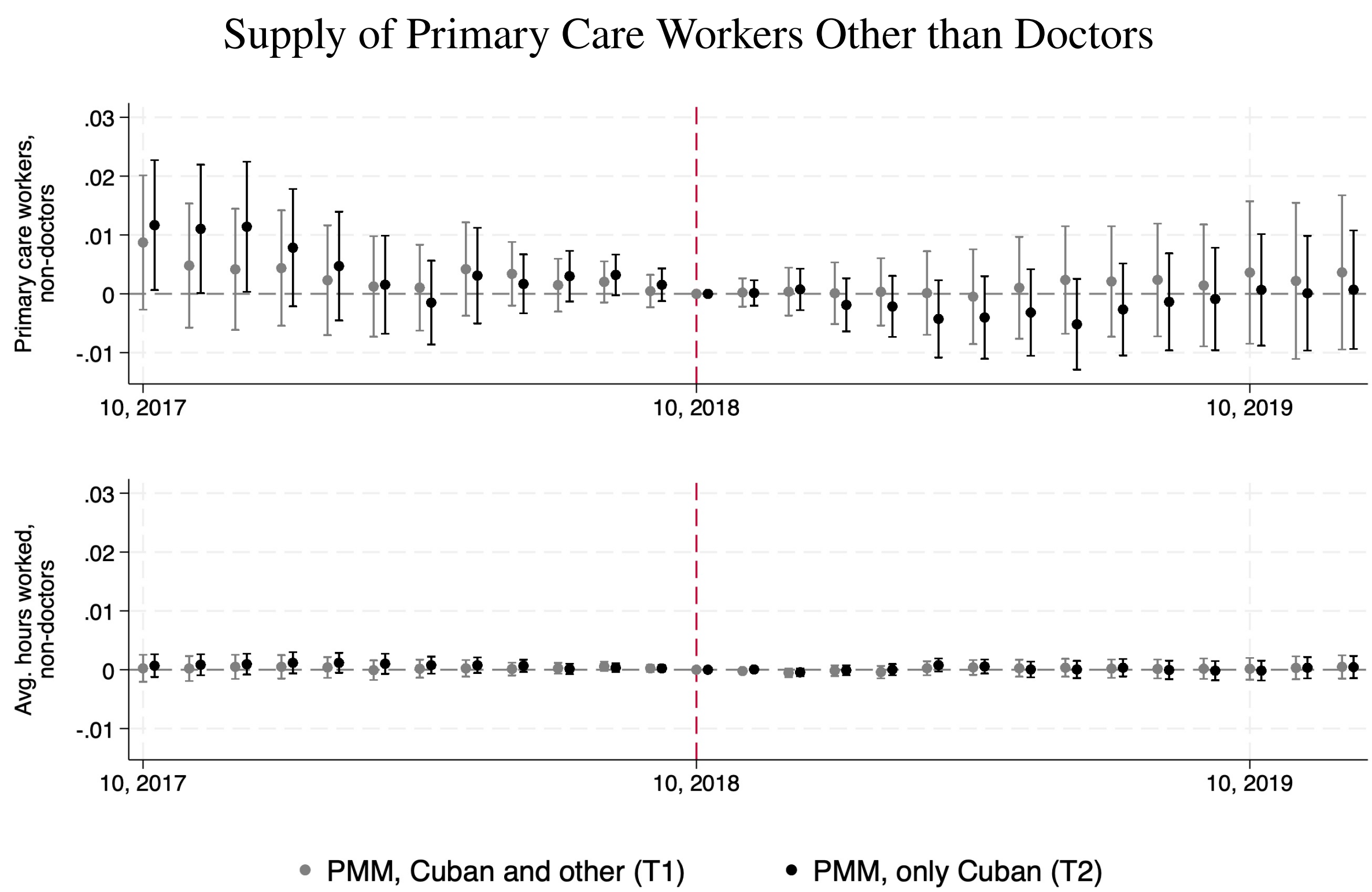

Figure D.3: The graph shows event studies for the number and average hours worked of non-doctors (nurses and community health workers) following equation 1. Regressions include municipality and state-by-time (month) fixed effects. Dependent variable adjusted by population size. Whiskers around the point estimates present 95 percent confidence intervals, where standard errors are clustered at the municipality level. The dashed vertical lines present the pre-treatment month October 2018. Joint p-value of pre-treatment coefficients in treatment group one (two): Primary care workers: 0.13 (0.04). Hours worked: 0.10 (0.98). Original data from CNES.

Primary Care Services Performed by Non-Doctors

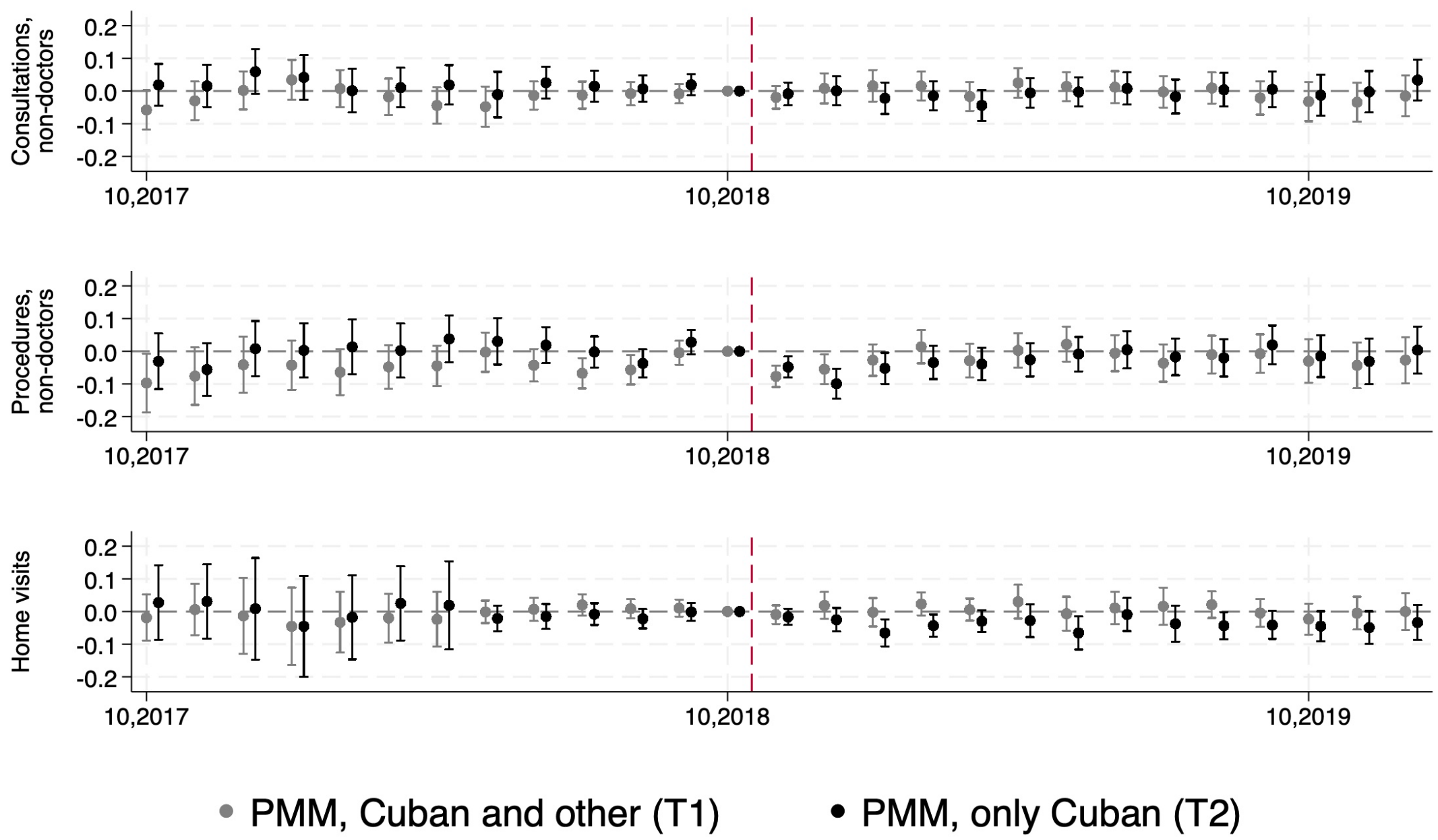

Figure D.4: The graph shows event studies for the number of consultations (upper panel), procedures (middle panel) and home visits (lower panel) performed in primary health care facilities by non-doctors, following equation 1. Regressions include municipality and state-by-time (month) fixed effects. Dependent variable measured per 100,000 inhabitants. Whiskers around the point estimates present 95 percent confidence intervals, where standard errors are clustered at the municipality level. The dashed vertical lines present the pre-treatment month October 2018. Joint p-value of pre-treatment coefficients in treatment group one (two): Consultations: 0.00 (0.19). Procedures: 0.00 (0.50). Home visits: 0.22 (0.48). Original data from SISAB.

D.2 Health Service Delivery

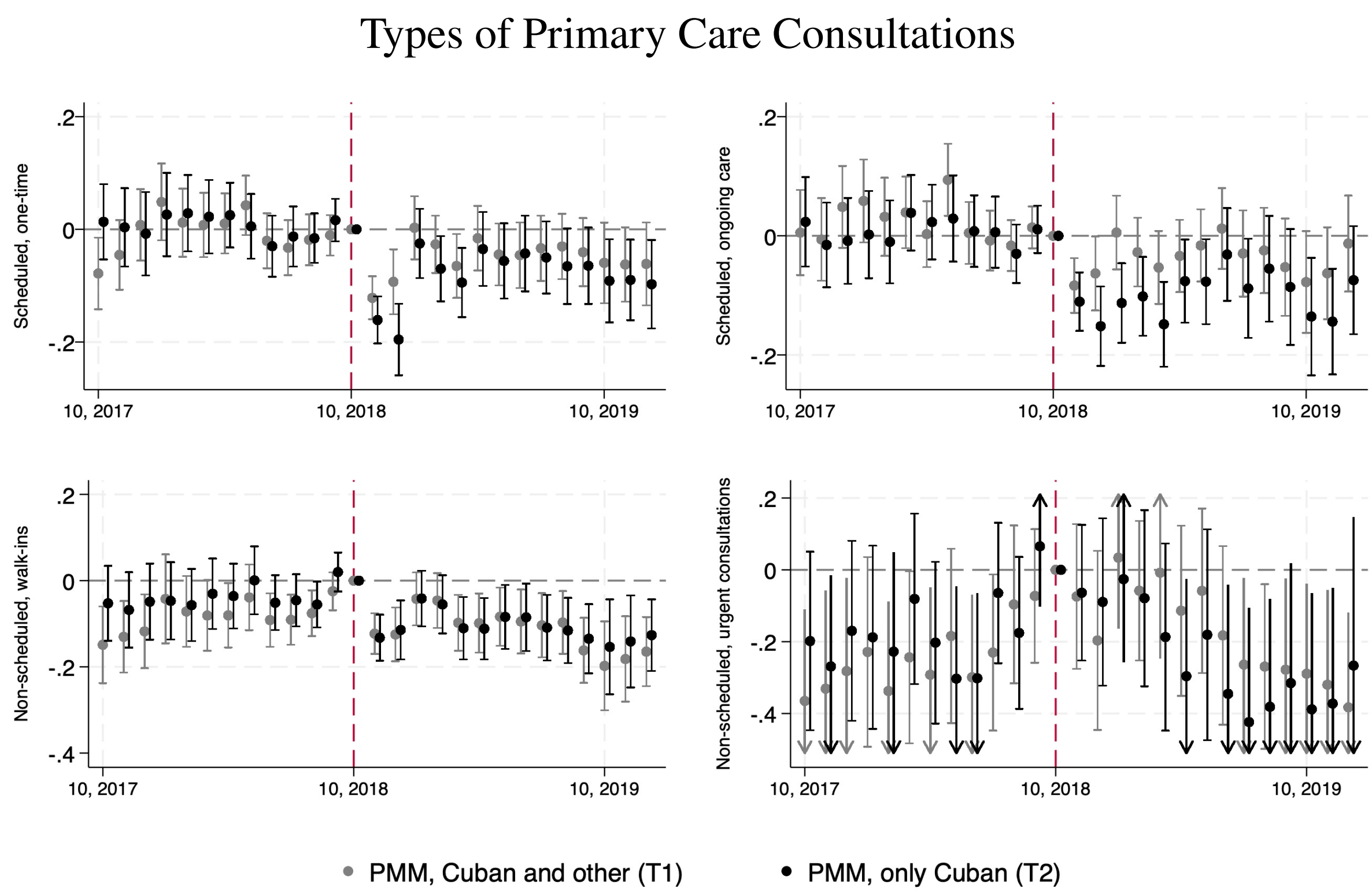

Figure D.5: The graph shows event studies for the number of consultations performed in primary health care facilities by the type of consultation, following equation 1. Regressions include municipality and state-by-time (month) fixed effects. Dependent variable measured per 100,000 inhabitants. Whiskers around the point estimates present 95 percent confidence intervals, where standard errors are clustered at the municipality level. The dashed vertical lines present the pre-treatment month October 2018. Joint p-value of pre-treatment coefficients in treatment group 1 (2): Scheduled one-time: 0.01 (0.44). Scheduled ongoing: 0.00 (0.35). Non-scheduled walk-ins: 0.09 (0.46). Non-scheduled urgent: 0.40 (0.09). Original data from SISAB.

Value of Subsidized Medication Sales on the Municipality Level

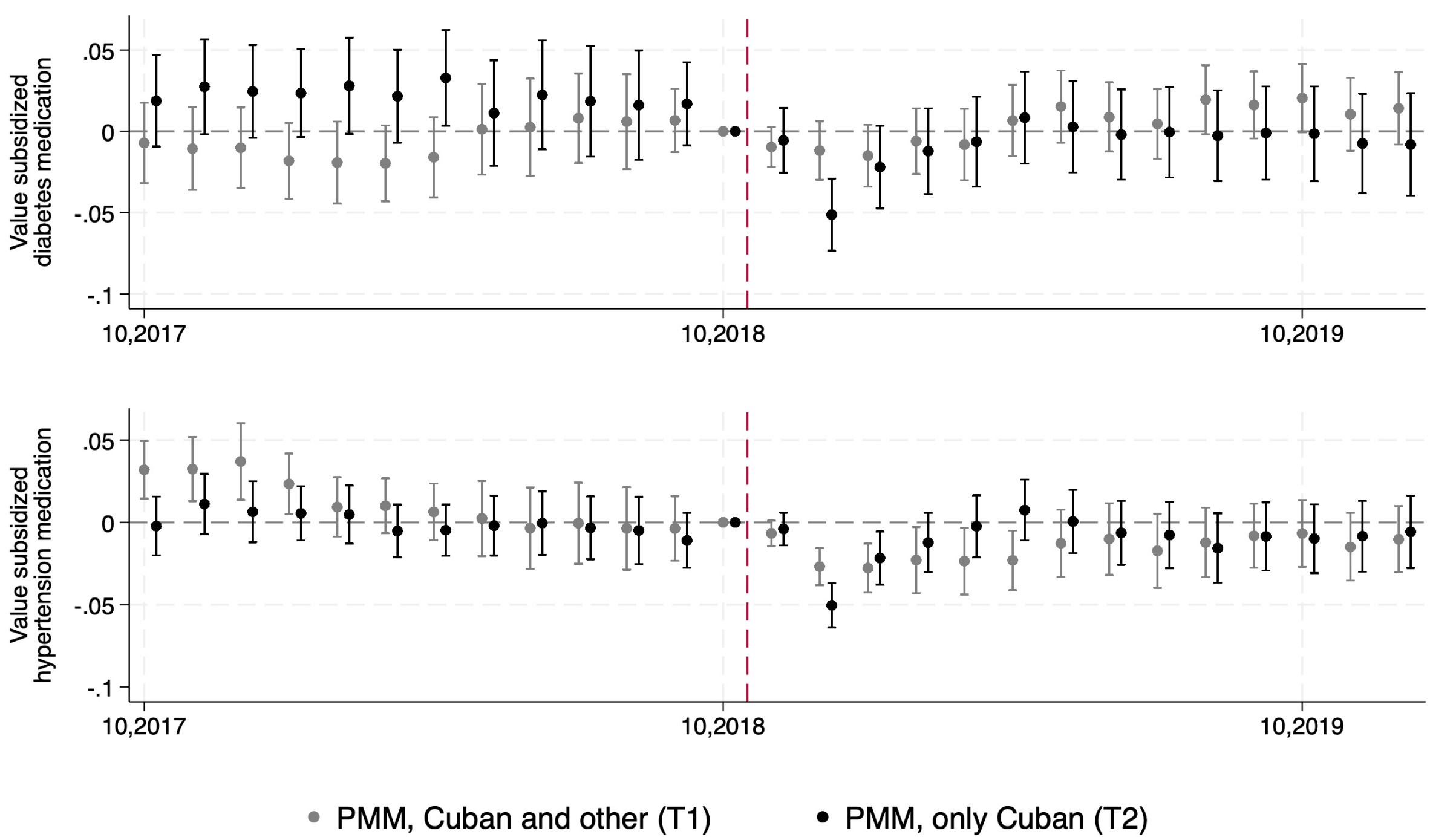

Figure D.6: The graph shows event studies for the value of prescription drugs for diabetes (upper panel) and hypertension (lower panel), distributed through *Aqui Tem Farmácia Popular*, following equation 1. Regressions include municipality and state-by-time (month) fixed effects. Dependent variable adjusted by population size. Whiskers around the point estimates present 95 percent confidence intervals, where standard errors are clustered at the municipality level. The dashed vertical lines present the pre-treatment month October 2018. Joint p-value of pre-treatment coefficients in treatment group 1 (2): Diabetes medication: 0.09 (0.07). Hypertension medication: 0.03 (0.01). Original data from the Ministry of Health.

D.3 What Changed Beyond Primary Care Centers?

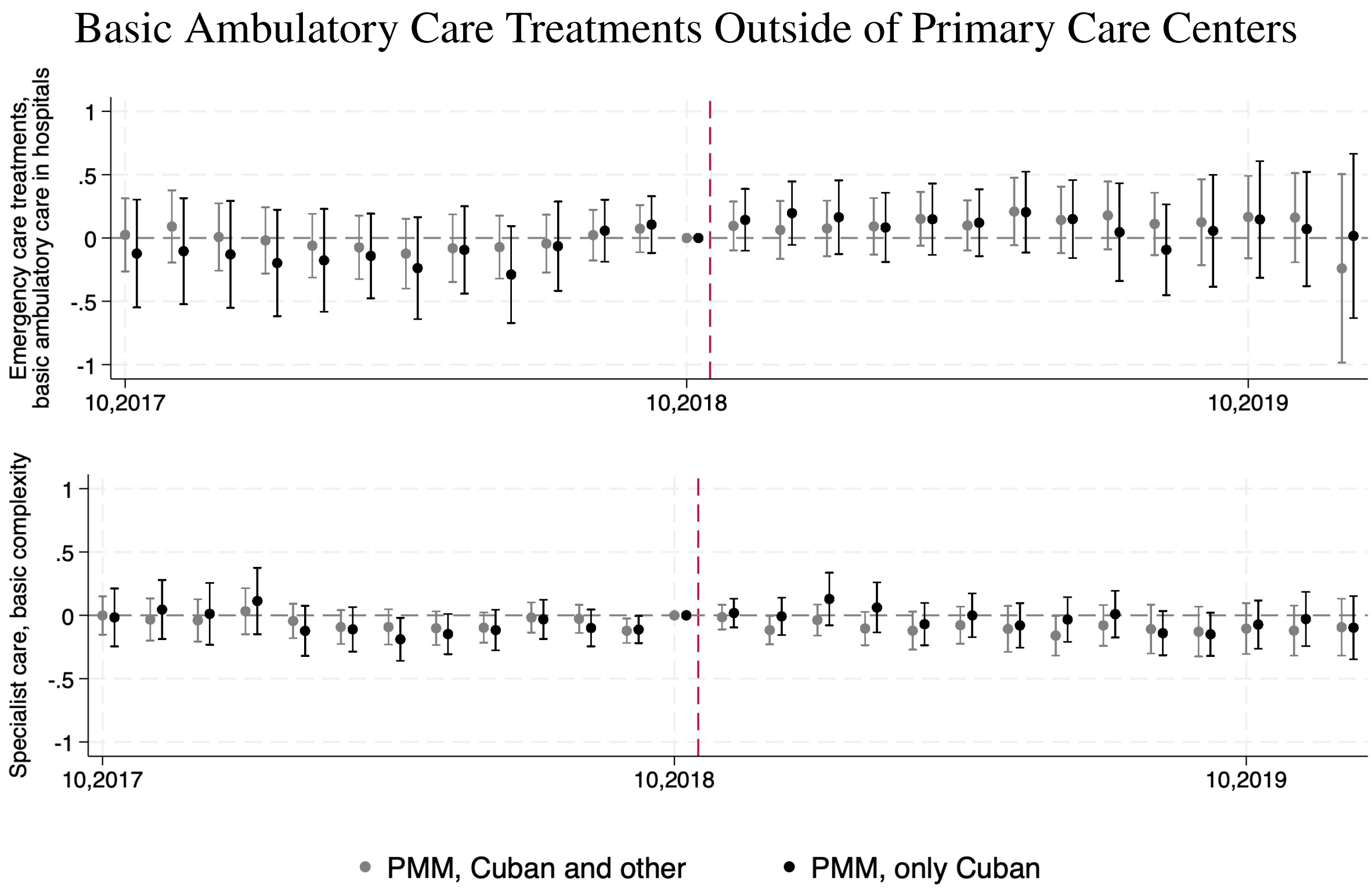

Figure D.7: The graph shows event studies for the number of basic ambulatory treatments at hospitals (upper panel) and specialists (bottom panel), following equation 1. Regressions include municipality and state-by-time (month) fixed effects. Dependent variable adjusted by population size. The series in grey presents treatment group 1, the series in black presents treatment group 2. Whiskers around the point estimates present 95 percent confidence intervals, where standard errors are clustered at the municipality level. The dashed vertical lines present the pre-treatment month October 2018. Joint p-value of pre-treatment coefficients in treatment group 1 (2): Hospital basic ambulatory care: 0.57 (0.03). Specialist basic care: 0.08 (0.19). Original data from the SIA/Datasus.

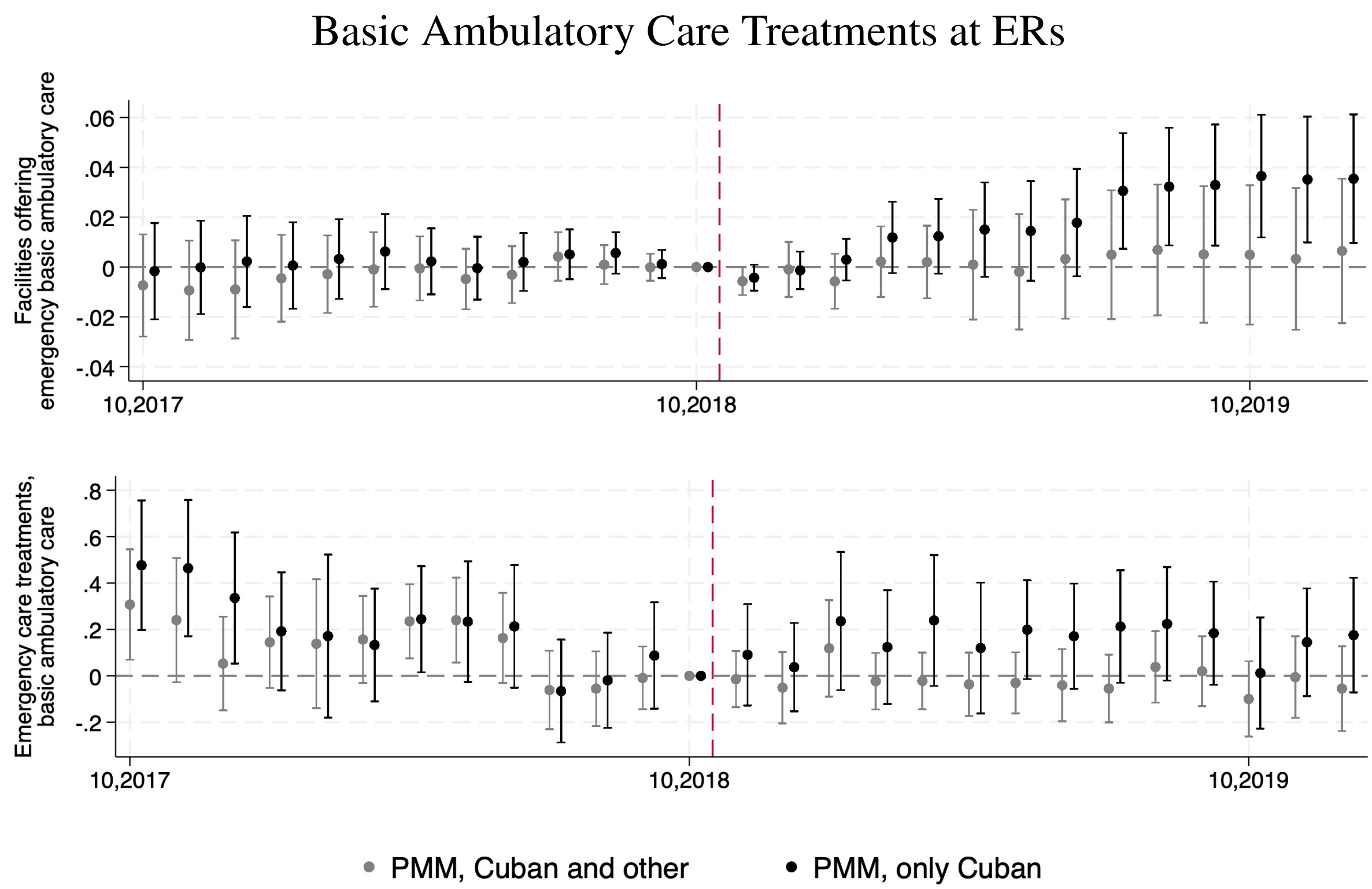

Figure D.8: The graph shows event studies for the number of emergency room treatments of basic ambulatory care (upper panel) and the number of ER facilities reported to have offered basic ambulatory care treatments (lower panel), following equation 1. Regressions include municipality and state-by-time (month) fixed effects. Dependent variable adjusted by population size. The series in grey presents treatment group 1, the series in black presents treatment group 2. Whiskers around the point estimates present 95 percent confidence intervals, where standard errors are clustered at the municipality level. The dashed vertical lines present the pre-treatment month October 2018. Joint p-value of pre-treatment coefficients in treatment group 1 (2): Facilities offering emergency basic care: 0.55 (0.76). Emergency care treatments basic care: 0.01 (0.00). Original data from SIA/Datasus and CNES.

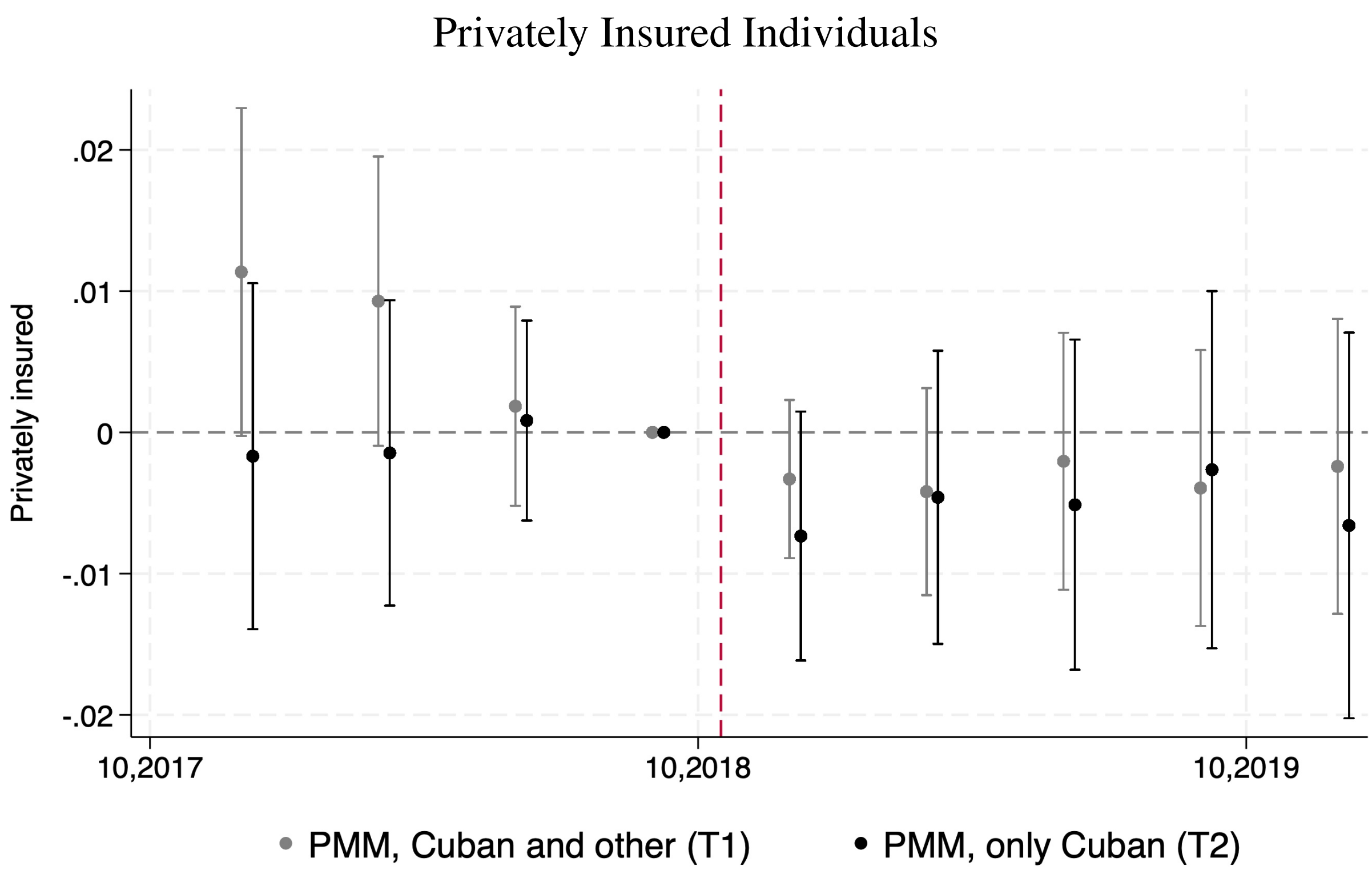

Figure D.9: The graph shows event studies for the number of privately insured people, following equation 1. The data is available every three months. Regressions include municipality and state-by-time (month) fixed effects. Dependent variable measured per 100,000 inhabitants. Whiskers around the point estimates present 95 percent confidence intervals, where standard errors are clustered at the municipality level. The dashed vertical lines present September 2018, the last month with data availability before the treatment. Joint p-value of pre-treatment coefficients in treatment group 1 (2): 0.01 (0.84) Original data from ANS.

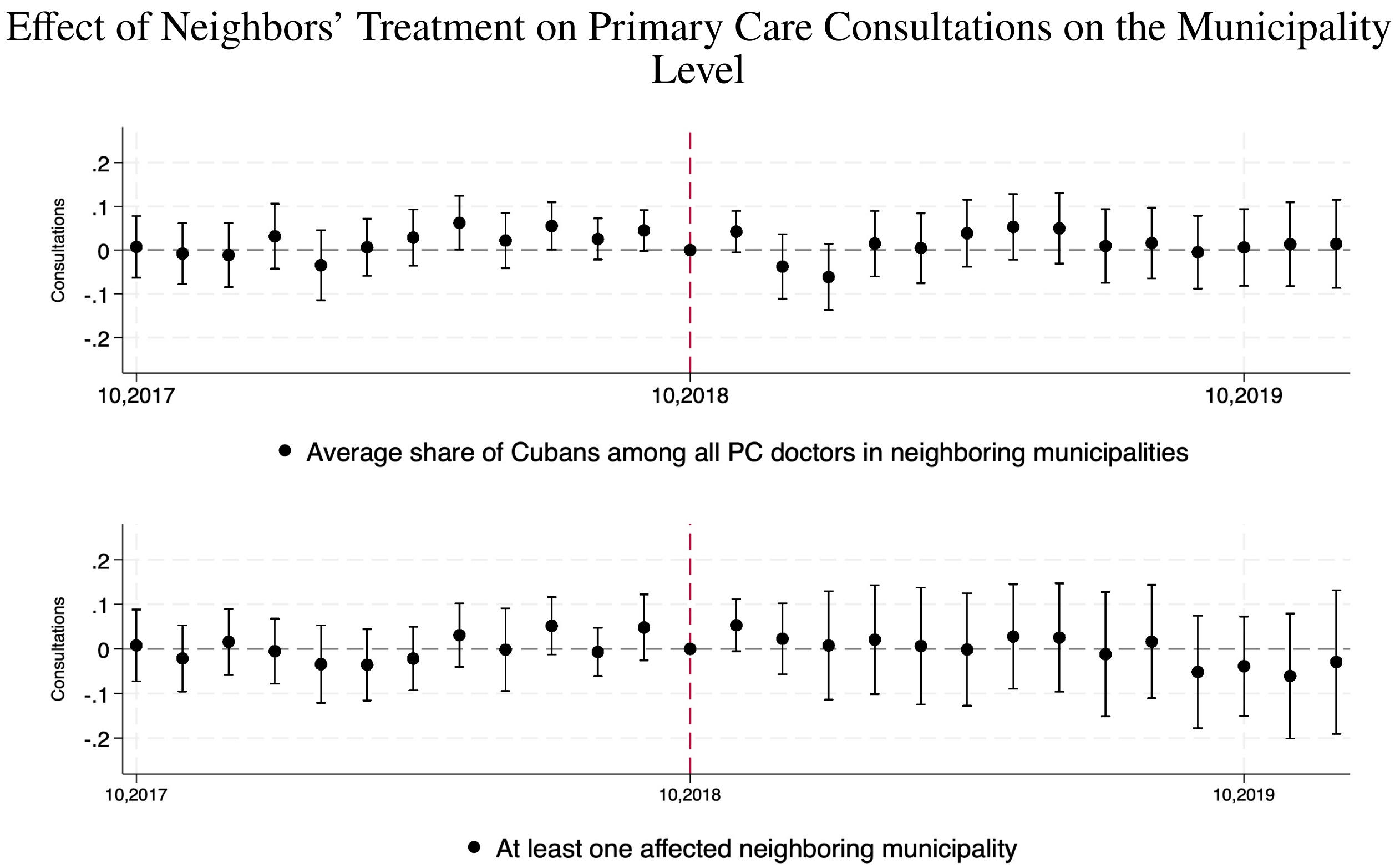

Figure D.10: The upper panel shows event studies for the effect of municipality *i*'s neighbors' average share of Cuban PMM doctors among all primary care doctors in October 2018 on municipality *i*'s primary care consultations, for all municipalities (PMM and non-PMM, N = 150,147). The lower panel shows event studies for the effect of having at least one treated neighboring municipality on municipality *i*'s primary care consultations in unaffected municipalities (non-PMM municipalities or control municipalities, N = 73,516). Neighbors' average treatment status is calculated using a row-standardized contiguity matrix. Regressions follow equation (2) and include municipality and state-by-time (month) fixed effects and control for municipality *i*'s own treatment. Dependent variable measured per 100,000 inhabitants. Whiskers around the point estimates present 95 percent confidence intervals, where standard errors are clustered at the municipality level. The dashed vertical lines present the pre-treatment month October 2018. Joint p-value of pre-treatment coefficients: Upper panel: 0.00. Lower panel: 0.02.

E Costs and Fiscal Implications

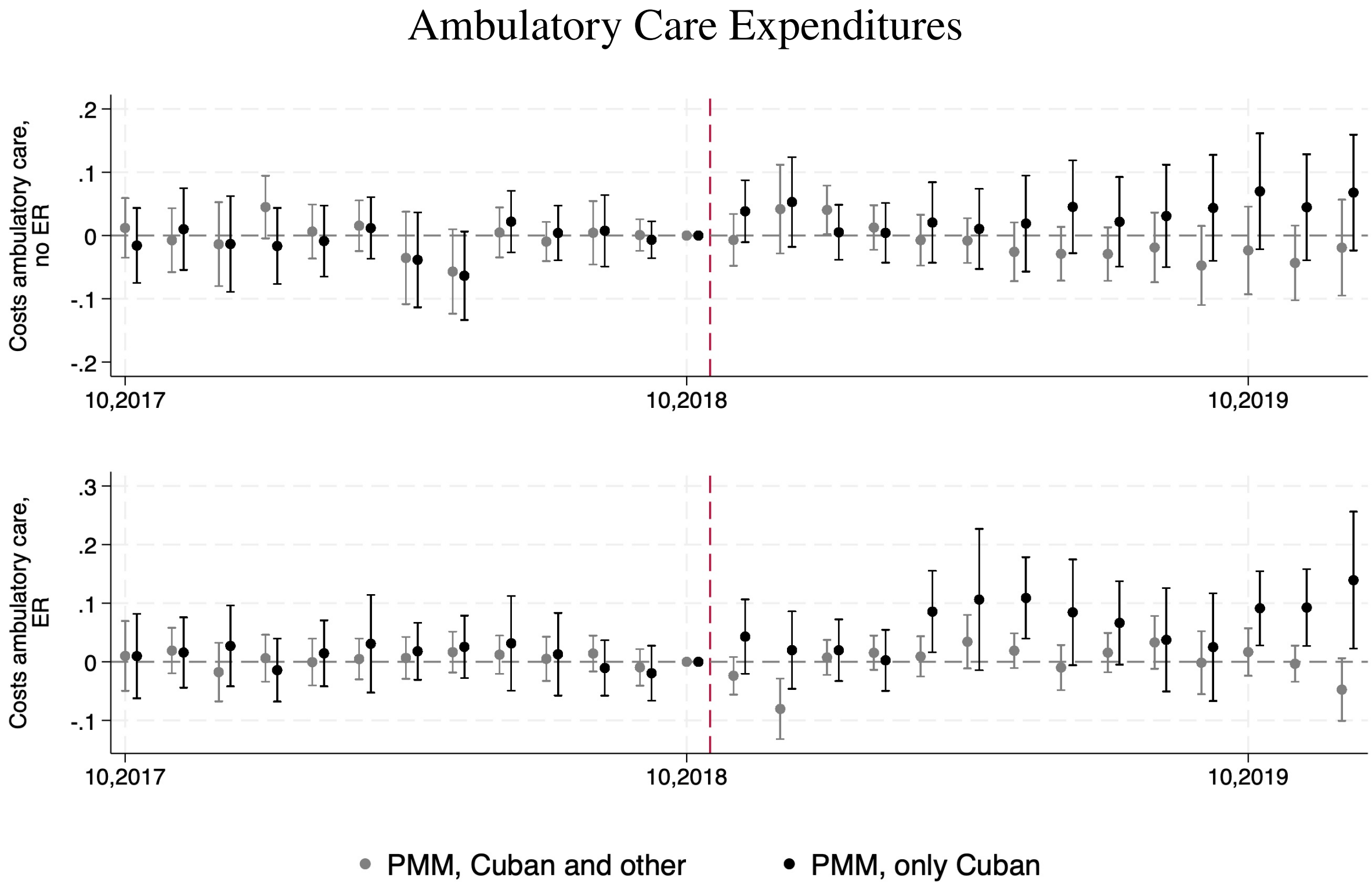

Figure E.1: The graph shows event studies for ambulatory care expenditures in facilities that do not offer emergency care (upper panel) and in facilities that offer emergency care (lower panel), following equation 1. Regressions include municipality and state-by-time (month) fixed effects. Outcomes are adjusted for population size. Whiskers around the point estimates present 95 percent confidence intervals, where standard errors are clustered at the municipality level. The dashed vertical lines present the pre-treatment month October 2018. Joint p-value of pre-treatment coefficients in treatment group one (two): Upper panel: 0.15 (0.31). Lower panel: 0.48 (0.47). Original data from SIA.
